# Changes to Serum Sample Tube and Processing Methodology Does Not Cause Inter-Individual Variation in Automated Whole Serum N-Glycan Profiling in Health and Disease

**DOI:** 10.1371/journal.pone.0123028

**Published:** 2015-04-01

**Authors:** Nicholas T. Ventham, Richard A. Gardner, Nicholas A. Kennedy, Archana Shubhakar, Rahul Kalla, Elaine R. Nimmo, IBD-BIOM Consortium, Daryl L. Fernandes, Jack Satsangi, Daniel I. R. Spencer

**Affiliations:** 1 Gastrointestinal Unit, Centre for Genetics and Molecular Medicine, University of Edinburgh, Western General Hospital, Edinburgh, EH4 2XU, United Kingdom; 2 Ludger Ltd, Culham Science Centre, Oxford, Oxfordshire, OX14 3EB, United Kingdom; Swiss Institute of Bioinformatics, SWITZERLAND

## Abstract

**Introduction:**

Serum N-glycans have been identified as putative biomarkers for numerous diseases. The impact of different serum sample tubes and processing methods on N-glycan analysis has received relatively little attention. This study aimed to determine the effect of different sample tubes and processing methods on the whole serum N-glycan profile in both health and disease. A secondary objective was to describe a robot automated N-glycan release, labeling and cleanup process for use in a biomarker discovery system.

**Methods:**

25 patients with active and quiescent inflammatory bowel disease and controls had three different serum sample tubes taken at the same draw. Two different processing methods were used for three types of tube (with and without gel-separation medium). Samples were randomised and processed in a blinded fashion. Whole serum N-glycan release, 2-aminobenzamide labeling and cleanup was automated using a Hamilton Microlab STARlet Liquid Handling robot. Samples were analysed using a hydrophilic interaction liquid chromatography/ethylene bridged hybrid(BEH) column on an ultra-high performance liquid chromatography instrument. Data were analysed quantitatively by pairwise correlation and hierarchical clustering using the area under each chromatogram peak. Qualitatively, a blinded assessor attempted to match chromatograms to each individual.

**Results:**

There was small intra-individual variation in serum N-glycan profiles from samples collected using different sample processing methods. Intra-individual correlation coefficients were between 0.99 and 1. Unsupervised hierarchical clustering and principal coordinate analyses accurately matched samples from the same individual. Qualitative analysis demonstrated good chromatogram overlay and a blinded assessor was able to accurately match individuals based on chromatogram profile, regardless of disease status.

**Conclusions:**

The three different serum sample tubes processed using the described methods cause minimal inter-individual variation in serum whole N-glycan profile when processed using an automated workstream. This has important implications for N-glycan biomarker discovery studies using different serum processing standard operating procedures.

## Introduction

Serum whole N-glycan profiles have been investigated as putative biomarkers in many complex immune disorders[1–6], including inflammatory bowel disease (IBD)[7,8] Both when seeking to identify biomarkers and to investigate underlying biological differences between health and disease, it is important to ensure observed changes are related to the disease and not a result of the sampling method. This is especially important for large multi-centre studies where standard operating procedures may be different amongst members of the study consortium.

The vast number of commercially available serum collection tubes were previously considered to be inert sample carriers with no potential to effect the measured analyte[9]. However several components of a serum collection tube can affect downstream assays; including the rubber stopper, tube wall material, surfactants, clot activators, and gel separators[9]. The separator gel acts a barrier to prevent contamination of the serum sample with cellular components, particularly erythrocytes. The presence of a gel separation medium is known to interfere with some but not all analytes, including hormones and drug levels [10,11]. Although data on the effect of gel on glycan analysis is lacking, gel can cause interference with the routine analytic techniques used to profile serum N-glycans including mass spectrometry and HPLC[12]. Additionally, the time taken to allow the sample to clot and conditions of pre-processing (temperature, centrifugation speed) may also effect analytical assays[13,14]. A study by Hsieh *et al* demonstrated diverse changes in the serum proteome using MALDI TOF mass spectrometry following changes in sample handling, prolonged clotting time (1 versus 24 hours) and storage temperature (4°C versus room temperature)[15]. The authors attributed changes to continued cellular metabolism, cellular lysis and consequent release of breakdown products and degradation products from the clot itself[15].

In the development of biomarkers, the first stage involves biomarker discovery[16]. To ensure the success of a putative biomarker, this first stage of discovery should be carefully performed to ensure markers are a result of the disease and not sampling factors. To limit the potential impact of human error, robot automation has been developed for the release, labeling and cleanup of glycans[17]. Critically for widespread biomarker utilization, automation may allow complex analytical techniques such as glycan profiling to become high-throughput[18].

The aim of this study was to determine the effect of different sample tubes and processing method on the whole serum N-glycan profile in health and disease. A secondary objective was to describe a robot automated N-glycan release, labeling and cleanup process for use in a biomarker discovery system.

## Materials and Methods

### Patient recruitment

Suitable IBD patients were prospectively recruited from gastroenterology clinic and endoscopy lists. Symptomatic controls consisted of patients undergoing investigations for suspected IBD, but following radiological/endoscopic investigations were found not to have IBD.

### Serum sample collection and initial processing

Blood sample collection was undertaken at the same time for research and clinical samples to minimize patient discomfort. A Greiner 21Gauge butterfly needle with 30cm safety tube with Luer lock device was used for venipuncture. The following tubes were taken from the same patient at the same draw: **Tube 1–**3.5ml vacuette plastic SST II Advance tube with gel separator, clot activator, and BD Hemograd closure (BD, no 367956), **Tube 2**–2.5ml Z serum clot activator vacuette tube with gel separator (Greiner, no 454243) and **Tube 3**- 9ml Z Serum clot activated vacuette (No gel, Greiner, no 455092). Serum tubes were taken before other clinical and research samples to prevent reagent contamination from other blood tubes (e.g. EDTA)[19]. Tubes were processed according to Table 1. Serum was aliquoted into 500μL screw cap tubes and stored at -80°C.

**Table 1:** Serum Tube types and Processing methods (RT = Room Temperature).

Tube	Primary tube material	Gel separator	Clot activator	Minimum clotting time (minutes)/ temperature (°C)	Centrifuge speed (g)	Centrifuge time (minutes)	Centrifuge Temp (°C)	Total processing time (minutes)
**Tube 1 BD gel**	Plastic	Acrylic based gel	Spray Dried	60/4	2,500g	15	4	60–360
**Tube 2 Greiner gel**	Plastic	Gel unknown type	Silica	30/ RT	2,000g	10	18	30–360
**Tube 3 Greiner plain**	Plastic	None	Silica	60/4	2,500g	15	4	60–360

### Ethics statement

The Tayside Committee on Medical Ethics B (Ninewells Hospital, Dundee, NHS Tayside, Scotland) granted approval for this study with all patients and controls giving written, informed consent (LREC 06/S1101/16, LREC 2000/4/192).

### Robot Automation

**Fig 1:**
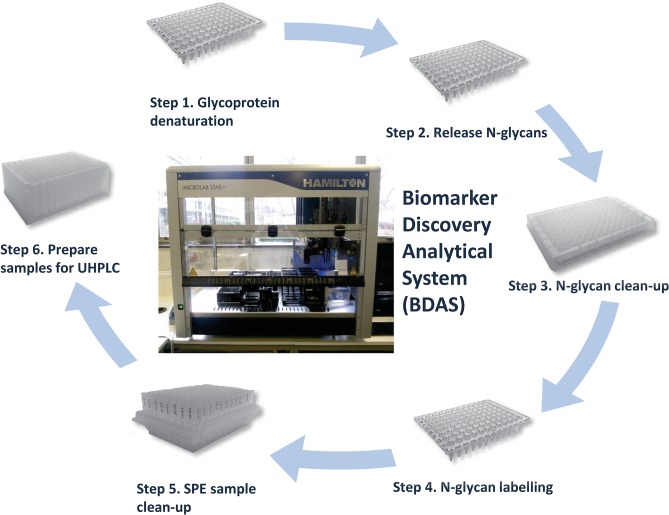
Biomarker discovery system workflow. (SPE = solid phase extraction, UHPLC = Ultra high performance liquid chromatography)

The sample processing for this was automated using the Hamilton Microlab STARlet liquid handling robot and is summarized in **Fig 1**.

### Step 1 and 2: Glycoprotein denaturation and N-glycan release

Ten microliters of each serum sample was aliquoted into a skirted 96 well PCR plate (4titude, 4ti-0960). The 96-well PCR plate was sealed with a pierce foil seal (4titude, 4ti-0531) and incubated at 100°C for 2 minutes. The pierce foil seal was carefully removed and to each sample was added 7.5 μL of de-ionised water and mixed. Reaction buffer x5 (5 μL, QAbio) and 1.25 μL of Denaturation solution (1 Molar β**-**mecaptoethanol and 2% SDS, QAbio) were added to each sample. The 96-well PCR plate was sealed with a pierce foil seal, samples mixed on a plate-shaker for 1–2 minutes, and centrifuged briefly to collect samples in the bottom of the wells. Samples were incubated for 10 minutes at 100°C. The samples were then allowed to cool to room temperature and the pierce foil seal carefully removed. To each sample was added 1.25 μL of Triton X, followed by 1 μL (2 μL diluted 1:1 with de-ionised water) of PNGase F (QAbio). The plate was again sealed with a pierce foil seal, mixed using the plate shaker, and centrifuged briefly. The sample was then incubated overnight at 37°C in the oven (17 hours +/- 1 hour). The pierce foil seal was carefully removed and the 96-well PCR plate of samples was placed in a rotary speed vac (room temp, no heat, 10 mBar, Thermo Savant) for 70 minutes to dry down the samples completely.

### Step 3: N-glycan clean-up

To the dried-down samples was added 20 μL of 1% formic acid (100 μL of formic acid in 9900 μL of water) followed by incubation for 50 minutes at room temperature. A Protein Binding Membrane (PBM) plate (LC-PBM-96, Ludger Ltd, UK) was washed with methanol (100 μL) and de-ionised water (300 μL). After each wash a vacuum was applied (−0.1 to −0.2 bar), using the integrated Hamilton vacuum manifold, to elute the wash through the membrane. The acidified sample was then transferred to the PBM plate. The initial PCR sample plate was washed with 100 μL of de-ionized water and transferred to the PBM plate. The PCR sample plate wash step was repeated once more and transferred to the PBM plate. A vacuum (−0.1 to −0.3 bar) was applied to elute the acidified sample and washings through the PBM plate and the eluent was collected within a 2 mL deep well collection plate (Ludger Ltd). The samples were then transferred back to a non-skirted 96 well PCR plate (4titude, 4ti-0710) and dried down again using a speed vac for 7±1 hours (room temp, no heat, 10 mBar, Thermo Savant).

### Step 4: N-Glycan labeling

The 2-AB labeling solution was prepared by adding 150 μL of DMSO/glacial acetic acid mix (Ludger Ltd) to the vial of 2-AB/2PB reductant (2-aminobenzamide, 2-picoline borane, Ludger Ltd). The solution was mixed until all the 2-AB/2PB reductant has dissolved. 10 μL of water was added to each of the dried down samples, followed by 10 μL of labeling reagent. The sample PCR plate was sealed with a pierce foil seal, mixed on a sample shaker, briefly centrifuged, and then incubated in an oven at 65 °C for 60 minutes. The sample was then cooled to room temperature.

### Step 5: SPE sample cleanup using HILIC (hydrophilic liquid interaction chromatography)

A HILIC method was performed using LC-T1 cartridges (Ludger Ltd) placed into a 96-well base plate (Ludger Ltd) placed upon the integrated Hamilton vacuum manifold. The LC-T1 cartridges were initially washed with 1 mL of water, and a vacuum applied (−0.1 to −0.2 bar) to aid the elution of the wash through the cartridge. The same process was repeated with 1 ml of 96% acetonitrile. The samples were then transferred to the LC-T1 cartridge by the addition of 80 μL of acetonitrile to each sample, subsequent mixing of the samples followed by the transfer of each diluted sample. 100 μL of acetonitrile was used to wash out the sample PCR plate and transferred to the LC-T1 cartridges to ensure all the sample has been transferred to the cartridges. Initially, the acetonitrile is allowed to pass through the cartridges by gravity, and after 10 minutes a vacuum (−0.05 to −0.2 bar) is applied slowly to elute any remaining acetonitrile. The cartridges are washed four times with 0.75 mL of 96% acetonitrile. After each wash addition, the 96% acetonitrile is left to elute under gravity for 4 minutes followed by a slow vacuum (−0.05 to −0.2 bar) to elute any remaining 96% acetonitrile through the cartridges. A higher vacuum (−0.2 to −0.5 bar) is used after the last wash elution step to remove as much 96% acetonitrile as possible from the cartridges. A 2 mL 96 deep well collection plate (Ludger Ltd) was then placed in the vacuum manifold under the cartridges. The 2-AB labelled N-glycans are then eluted using 1 mL of water. A low vacuum setting (−0.05 bar, 10 seconds) was used to start the elution followed by gravity elution for 15 minutes. A higher vacuum setting (−0.1 to −0.5 bar) was used to elute any remaining water from the cartridges.

### Step 6: Sample preparation for Ultra-high performance liquid chromatography (UHPLC)

Samples were prepared for UHPLC by taking 110 μL of each 2-AB labelled glycan sample and mixing with 390 μL of acetonitrile in a 96 deep well collection plate. The plate of samples was covered with a pierce silicon sealing mat (Ludger Ltd) and placed directly in the UHPLC and the samples analysed by HILIC-UHPLC using a Dionex UltiMate 3000 dual gradient system UHPLC fitted with a BEH-Glycan 1.7 μm, 2.1 x 150 mm column (Waters, UK) at 40 °C and a U3000 fluorescence detector set at excitation wavelength of 250 nm, emission wavelength of 428 nm, sensitivity = 8, lamp energy = high, controlled by Chromeleon data software version 6.8 (Dionex, USA).

A binary separation gradient was utilised where solvent A was 50 mM ammonium formate made from LudgerSep N Buffer stock solution, pH4.4 (Ludger Ltd) and solvent B was acetonitrile (Acetonitrile 190 far UV/gradient quality; Romil #H049, Charlton Scientific, UK). Gradient conditions were: 0 to 5 min, 24% A (0.4 mL/min); 5 to 38.5 min, 24 to 42% A (0.4 mL/min); 38.5 to 40.5 min, 42 to 60% A (0.4 to 0.25 mL/min); 40.5 to 42.5 min, 60% A (0.25 mL/min); 42.5 to 44.5, 60 to 24% A (0.25 mL/min); 44.5 to 50.5 min 24% A (0.25 mL/min); 50.5 to 51.5 min 24% A (0.25 to 0.4 mL/min); 51.5 to 55.0 min 24% A (0.4 mL/min). Samples were injected directly from the 96 deep well collection plate (22% aqueous/78% acetonitrile); injection volume 25 μL, sample loop 50 μL size with 80% acetonitrile solvent used for UHPLC loop and needle washing and to make up the injection volume to 50 μL for the U3000 partial mode injection setting. A 2-AB labelled glucose homopolymer (Ludger Ltd), was used as a system suitability standard as well as an external calibration standard for GU allocation of the system.

### Evaluation of automated N-glycan release protocol for use in a biomarker discovery system

To assess the reproducibility of the newly developed automated N-glycan release protocol standard samples were processed in replicate. The plasma IgG N-glycan and whole plasma N-glycan profile was assessed using pooled human IgG glycan (G4386-10G, Sigma Aldrich, St Louis, MO, USA) and pooled human plasma (P9523-5ML, Sigma Aldrich, St Louis, MO, USA) respectively.

### Quantitative correlation of samples

Peak identification and integration was performed using a custom algorithm written using R 3.1.1 (R Foundation for statistical computing, Vienna Austria). Raw chromatograms were exported from Chromeleon 7.1 (Dionex, USA). These were imported into R and then normalized using the ChemoSpec package[20].The time axis of the chromatograms was aligned using the highest peak as a reference. Peaks were identified using the first and second derivatives generated using the glkerns function from the lokern package[21]. Peak positions were grouped across using samples using hierarchical clustering, and peaks identified in at least 50% of samples were kept, resulting in 42 peaks in the final dataset. Where a corresponding peak was not present in a chromatogram, usually in regions of overlapping peaks, the surrounding peaks were used to estimate its position. Peak area was then defined as the integral of the chromatogram between perpendicular lines dropped from the troughs. The peak identification was checked visually using plots of the chromatograms and identified peaks. This method ensured that the same glycans were quantified across all samples. The peak areas were normalized to the sum of all peaks. Peaks within the neutral region of the chromatogram were also analysed separately and normalized to the sum of just that subset. Variability of measured glycan levels was expressed as the average coefficient of variation using log-transformed data ($CV\;=\;\sqrt{e^{s_{ln}^{2}}-1}$). Pairwise comparisons between each sample were performed using Pearson’s correlation efficient. Hierarchical clustering was performed using a distance metric of (1−|Pearson’s r|) and complete linkage. Principal co-ordinate analysis was done using classical multidimensional scaling of the (1−|Pearson’s r|) distance matrix. Demographic data were assumed to be non-normally distributed and Wilcoxon rank sum and Fisher’s exact test were used for comparisons. Differences at the level of individual glycans were assessed using analysis of variance of the log-transformed data from individuals who had all three types of sample. The patient of origin was used as a blocking variable.

Samples are named using an arbitrary letter for each participant, and a number for each tube type, corresponding to the numbering in Table 1.

### Qualitative assessment of serum samples

Chromatograms were assigned a numerical sample code, to which the assessor was blinded. Chromatograms were classified into five groups according to the neutral/immunoglobulin-type glycan section of the chromatograms (retention time 16 to 24 min) and their comparability to the glycoprofile of glycans released from normal human gamma globulins (Sigma, UK). The classes were ‘Normal’, identified as ‘NGal’ i.e. the serum neutral glycan region of the glycoprofile was similar to the gamma globulin glycans with similar levels of biantennary, core fucosylated glycan galactosylation, ‘Higher Galactosylation—HGal’ where biantennary core fucosylated glycan galactosylation levels were higher than the gamma globulin levels, ‘Much Higher Galactosylation—MHGal’, where biantennary core fucosylated glycan galactosylation levels were much higher than the gamma globulin levels, ‘Lower Galactosylation—LGal’, where biantennary core fucosylated glycan galactosylation levels were lower than the gamma globulin levels and ‘Much Lower Galactosylation—MLGal’, where biantennary core fucosylated glycan galactosylation levels were much lower than the gamma globulin levels. After classification into the five galactosylation levels, chromatograms were visually matched.

## Results

### An automated N-glycan release protocol for use in a biomarker discovery system

The first outcome of this study is the description of an automated N-glycan release, labeling and clean-up process using a Hamilton Microlab STARlet liquid handling robot (Fig 1). Established manual methods were optimized and adapted to produce an automated high throughput (HTP) method in a 96 well plate based format. The reproducibility of the automated HTP method was assessed by processing pooled samples of human serum IgG (repeated 48 times) and whole plasma N-glycan (repeated 24 times) in replicate. For both pooled serum IgG glycan and whole plasma N-glycan profiles the Pearson’s coefficient demonstrated a high level of correlation of normalised peak areas (IgG glycans mean 0.9998 (range 0.9993–0.9999), Plasma whole N-glycans mean 0.99991 (range 0.9996–0.99998)) (S1 Fig). The complete dataset is available in S1 and S2 Data.

### Patient demographics

The demographics of IBD patients and controls are displayed in Table 2. All patients were White European ethnicity and ate a normal diet consisting of mixed meat, fish and vegetables. The C-reactive protein was significantly higher in CD and symptomatic controls compared to UC (vs. CD p = 0.01, vs. Symptomatic Control p = 0.04).

**Table 2:** Patient demographics (CD = Crohn’s disease, UC = ulcerative colitis, IQR = interquartile range, * = Wilcox sum rank test, † = χ^2^ with Yate’s continuity correction, mg = milligrams, L = Litre).

	Crohn’s disease (n = 7)	CD vs Symptomatic control	Symptomatic control (n = 7)	UC vs Symptomatic control	Ulcerative colitis (n = 10)
**Number of females (%)**	2/7 (28.6)	p = 1 †	4/7 (57.1)	p = 1 †	3/10 (30)
**Age (median, IQR)**	28 (26.8–35.3)	p = 0.9*	28 (26–31.5)	p = 0.2*	50 (32.8–59.5)
**Current or ex-smoker at time of sampling (%)**	2/7 (28.6)	p = 1 †	3/7(42.9)	p = 0.5†	7/10(70)
**White cell count (x10** ^**9**^ **/L)**	7 (6.3–8.5)	p = 0.2*	5.8(5.5–6.2)	p = 0.3*	6.7(5.8–8.5)
**C-reactive protein (mg/L)**	13(6.3–21.3) Available from 5/7 patients	p = 0.8*	10(4–13) Available from 5/7 patients	p = 0.04*	2(1.25–2.5) Available from 6/7 patients
**Albumin (g/L)**	42(42–42)	p = 0.3*	40.5(39.3–40.9)	p = 0.6*	40(39–40)
**Fecal calprotectin (**μg/g)	180(110–370) Available from 7/7 patients	p = 0.2*	1(1–60.8) Available from 3/7 patients	p = 1*	1(1–220.8) Available from 3/10 patients

### Quantitative assessment of intra-individual variation in glycan profile

**Fig 2:**
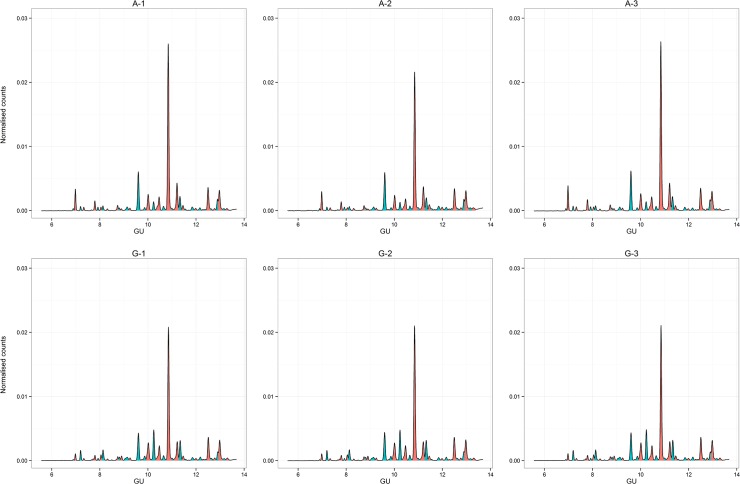
Fluorescence chromatogram showing a 2-aminobenzamide labeled glucose homopolymer (GHP) run on a UHPLC HILIC column. Fig 2A–2C and 2D–2F are derived from different patients respectively. Peaks are labeled arbitrarily in order from 1 to 42, with glucose unit values according to Guile *et a*l [22] Peaks are colored alternately to aid identification.

The complete chromatogram dataset is available in S1 Table, S2 Fig and S3 Data Chromatogram peaks were labeled in order from 1 to 42 (**Fig 2**). Glycan peaks were divided into neutral (peaks 1 to 16) and total serum N-glycans (all peaks).

**Fig 3:**
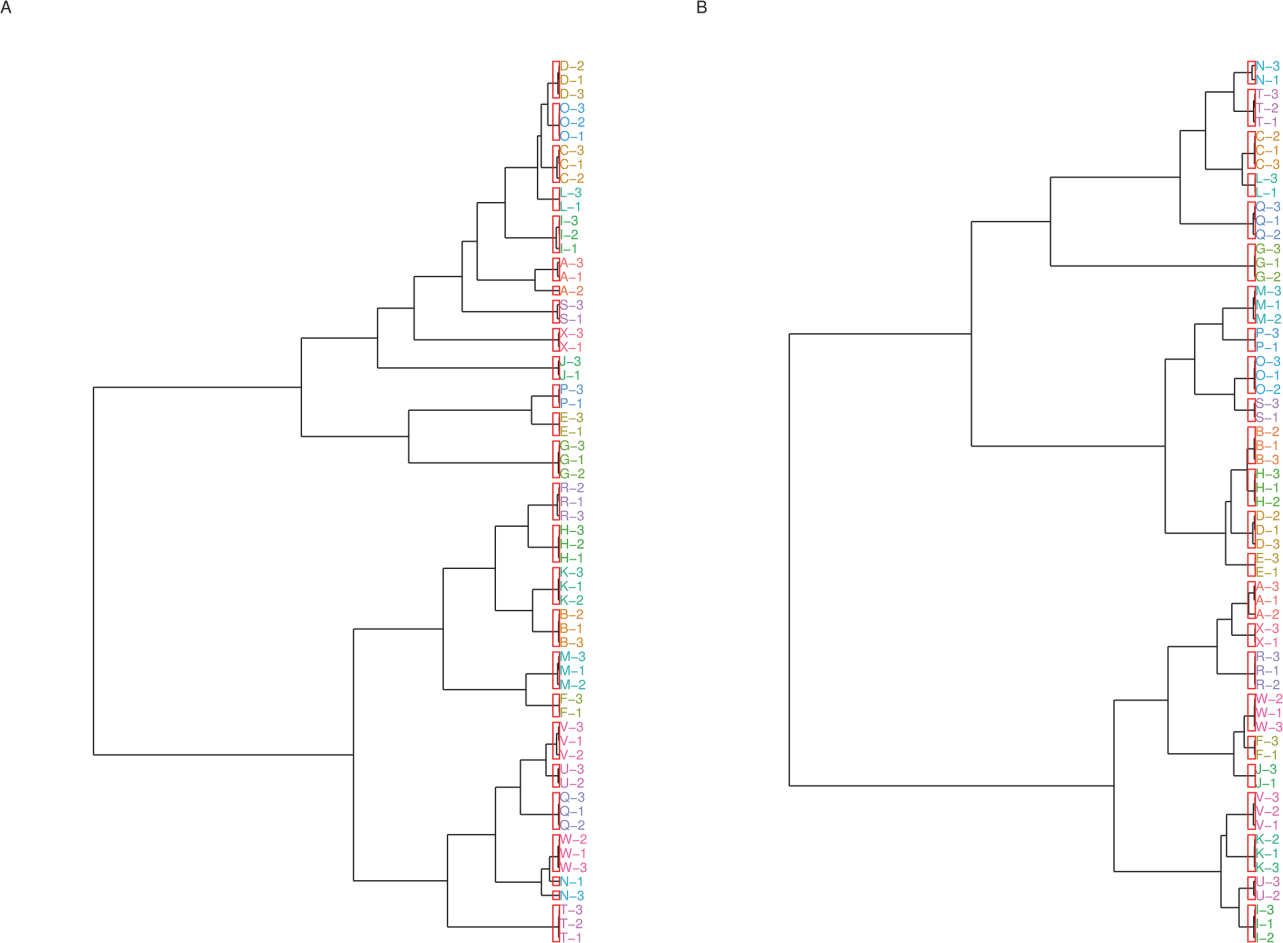
Clustering correlation complete linkage a) Neutral glycans b) Total glycans. Samples from the same individual are labeled using the same letter.

**Fig 4:**
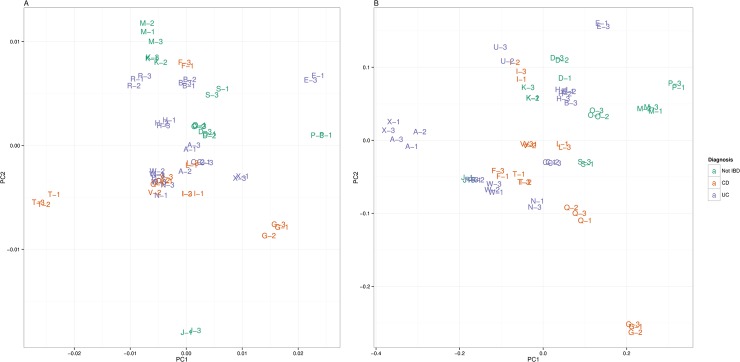
Principal coordinate analysis plot using classical multidimensional scaling of the (1−|Pearson’s r|) distance matrix for a) Neutral serum N-glycans b) Total serum N-glycans (CD: Crohn’s disease; UC: Ulcerative colitis; IBD: Inflammatory bowel disease).

Pairwise comparison of samples demonstrated good intra-individual correlation (Pearson’s coefficient 0.99–1.0) (S2 Table). Hierarchical clustering demonstrated a good ability to match samples from the same individual (Fig 3). Complete correlation linkage using neutral glycans paired all samples from the same individual in all cases, although one sample of three from individual A came from a different 6^th^ order branch, but the same fifth order branch (Fig 3A). Correlation linkage using total serum N-glycan structures paired samples from the same individual slightly less well, with several samples from the same individual originating from slightly different lower order branches, but the same higher order branches (Fig 3B). Clustering of samples was visualized using principal coordinate analysis with individual samples according to disease status. Again, samples from the same individual appear to cluster together with the exception of individual A. There was no apparent clustering according to disease status (Fig 4).

Geometric mean peak areas and coefficients of variation (CV) for individual glycans can be seen in Table 3. The CV for some of the smaller peaks was quite high, especially peaks 11 and 30, but was less than 6% for all peaks with at least 1% of the total area. Analysis of variance (ANOVA) of the individual glycans revealed no differences that were significant by sample type (minimum uncorrected p value 0.015, but 0.62 after Bonferroni correction for multiple testing).

**Table 3:** Mean peak areas, coefficients of variation for peaks and tests of glycans against tube type.

Neural				
Peak	GU	Geometric mean % peak area	Mean CV %	P
1	5.89	0.13	14.22	1.00
**2**	**5.98**	**1.54**	**4.34**	1.00
3	6.19	0.85	3.19	1.00
4	6.32	0.35	8.77	1.00
5	6.61	0.09	24.91	1.00
**6**	**6.75**	**1.50**	**3.65**	1.00
7	6.88	0.58	4.89	1.00
8	6.99	0.59	3.32	1.00
**9**	**7.07**	**1.27**	**2.10**	1.00
10	7.25	0.39	10.99	1.00
11	7.42	0.06	51.62	1.00
**12**	**7.65**	**1.26**	**3.42**	1.00
13	7.72	0.36	6.18	1.00
14	7.81	0.45	7.93	1.00
**15**	**8.04**	**1.19**	**5.27**	1.00
16	8.15	0.37	11.24	1.00
**Charged**				
**Peak**	**GU**	**Geometric mean % peak area**	**Mean CV %**	**P**
**17**	**8.49**	**8.05**	**1.36**	0.62
18	8.64	0.18	21.38	1.00
19	8.75	0.76	4.14	1.00
**20**	**8.90**	**4.05**	**1.82**	1.00
**21**	**9.13**	**2.19**	**5.99**	1.00
**22**	**9.37**	**4.39**	**1.12**	0.77
**23**	**9.55**	**1.35**	**4.47**	1.00
**24**	**9.77**	**36.29**	**2.29**	1.00
25	9.93	0.52	9.73	1.00
**26**	**10.16**	**5.33**	**1.31**	1.00
**27**	**10.30**	**2.37**	**1.39**	1.00
**28**	**10.44**	**1.20**	**2.77**	1.00
29	10.55	0.29	9.37	1.00
30	10.70	0.08	39.01	1.00
**31**	**10.88**	**1.14**	**5.83**	1.00
32	11.05	0.61	7.26	1.00
33	11.26	0.91	5.94	1.00
34	11.40	0.24	13.61	1.00
35	11.51	0.30	12.72	1.00
**36**	**11.65**	**5.91**	**2.24**	1.00
37	11.79	0.46	7.27	1.00
38	11.95	0.55	6.02	1.00
**39**	**12.09**	**2.32**	**2.20**	1.00
**40**	**12.17**	**6.05**	**3.28**	1.00
41	12.38	0.62	6.19	1.00
42	12.50	0.79	7.48	1.00

Rows where geometric mean peak area >1% are highlighted in bold. CV = Coefficient of variation, calculated on log-transformed data. P = Bonferroni-corrected p value from analysis of variance (ANOVA) against sample type with patient of origin as a blocking factor.

### Qualitative assessment of intra-individual variation in glycan profile

By overlaying chromatograms from the 25 patients samples the blinded assessor was able to correctly match patient samples in 18 of the 25 patients representing a 72% success rate (Table 4).

**Table 4:** Qualitative matching of chromatograms.

Chromatogram group	Galactosylation Class			Sample name			Diagnosis
1	LGal	NGal	NGal	G3	G1	G2	CD
2	MLGal	MLGal	MLGal	R1	R3	R2	UC
3	LGal	LGal	HGal	*N1*	*V1*	*L3*	-
4	NGal	NGal	NGal	I1	I2	I3	CD
5	LGal	LGal		U2	U3		UC
6	NGal	LGal	NGal	Q2	Q1	Q3	CD
7	NGal	NGal	NGal	K2	K3	K1	SC
8	MLGal	MLGal	LGal	T2	T3	T1	CD
9	HGal	HGal	HGal	H3	H2	H1	UC
10	HGal	HGal	HGal	D1	D3	D2	SC
11	HGal	NGal		S1	S3		SC
12	MLGal	MLGal		X1	X3		UC
13	LGal	LGal	LGal	C1	C3	C2	UC
14	MLGal	MLGal	LGal	J3	J1	*N3*	-
15	MHGal	MHGal	MHGal	*P1*	E1	E3	-
16	HGal	HGal	HGal	O3	O2	O1	SC
17	HGal	HGal	HGal	B1	B3	B2	UC
18	LGal	MLGal	MLGal	W1	W2	W3	UC
19	HGal	LGal	LGal	*L1*	V3	V2	CD
20	MLGal	MLGal	LGal	A1	A3	A2	UC
21	HGal	HGal	HGal	M2	M1	M3	SC
22	MLGal	MLGal		F1	F3		CD
23	HGal			P3			SC

Samples that failed to be matched correctly are highlighted in italics. NGal: Profile similar to normal human gamma globulin; HGal: Higher galactosylation; MHGal: Much higher galactosylation; LGal: Lower galactosylation; MLGal: Much lower galactosylation CD: Crohn’s disease; UC: Ulcerative colitis; SC: Symptomatic control.

## Discussion

This study demonstrates minimal inter-individual variation in serum N-glycan profiling following three different methods of serum tube processing. Robot processing of samples in this study demonstrates feasibility of high-throughput, automated serum N-glycan profiling studies that in future may be used as part of a biomarker discovery system.

Several studies have shown significant variation in glycans between healthy individuals within the sample population.[23] In the context of disease, temporal changes in the glycan profile have been noted for the same individual over both short and long periods of time.[3] However in healthy individuals, the N-glycan profile is relatively stable for up to five days.[24] The aforementioned study noted that certain glycans demonstrated greater inter-individual variability than others.[24] Given the large number of factors that can affect the glycan profile, this study demonstrates that three different methods of sample handling used did not significantly affect the N-glycan profile. Several elements of the serum collection tube are known to affect various clinical biochemical assays.[9] The present study suggests that factors including clot activators and gel separator medium do not profoundly affect the serum glycan profile.

The strengths of this study include the combination of both quantitative and qualitative methods to compare the glycan profile within- and between individuals. Unsupervised, unbiased, quantitative methods such as hierarchical clustering and multidimensional scaling plots accurately clustered samples from the same individual together. Blinded qualitative assessment confirmed that chromatograms could easily be matched ‘by eye’. In the quantitative analyses, the chromatogram was considered in its entirety and in a subsection denoting neutral glycans. This neutral glycan area of the chromatogram consists mainly, but not entirely, of IgG associated glycans. There is large inter-individual variation in the IgG glycome in the general population and the relative proportions of these glycans are indicative in diseases such as rheumatoid arthritis[25,26]. We were able to demonstrate good intra-individual correlation in spite of these changes noted in inflammatory diseases.

A second source of considerable chromatogram variation is serum glycans that terminate in sialic acid. These nine carbon chain acidic monosaccharides which impart charge onto glycans are notoriously labile under conditions of heat and acidity and much work has been done to reduce their degradation during glycan analysis[27,28]. Incubation at a high temperature (100 °C) during the N-glycan release may result in loss of terminal sialic acid. Whilst this may be relevant for future studies, all samples were treated uniformly prior to comparison this methodological study. Variations in sialic acid groups are often seen in common human diseases[29]; it is therefore important to ensure that technical variation does not interfere with the ability to compare such data. This study suggests that for sialylation, as with neutral glycans, differences between individuals were much greater than between differently processed samples from the same individual (S3 Fig).

Several limitations of the present study should be noted. Very small peaks were excluded, but the smallest of the included peaks still exhibited a relatively high coefficient of variation. Minimal intra-individual variation was noted between the three sampling methods used in this study, however the number of included samples was relatively small and there is a risk of type II error. Moreover, the findings of this study may not be generalisable to other serum tube/processing methods. Future studies should compare multiple post-collection processing methods using broader ranges of centrifugation speed and time, serum coagulation time and temperature. Significantly, this study does not address technical variation introduced by different people processing samples nor variation between centers.

This study did not aim to compare glycan profiles between cases and control, nor infer any biological consequence of the differences in glycans observed. This study was not powered to detect glycan differences between cases and controls. Larger case-control studies have been published [7] and international consortia are working towards addressing this question(www.ibdbiom.eu). Only patients with IBD were included in this study; it is unclear whether the findings would be applicable to serum N-glycan profiling in other inflammatory conditions. However, this study did demonstrate that measurement of serum glycans is robust to variation in sample processing in IBD patients as well as controls.

## Conclusion

The three different serum sample tubes processed using the described methods cause minimal inter-individual variation in serum whole N-glycan profile when processed using an automated workstream. This has important implications for N-glycan biomarker discovery studies using different serum processing standard operating procedures.

## Supporting Information

S1 Table Complete chromatogram dataset. Patients are labeled A to X, with each patient having between 2 and 3 samples denoted by the letter. Data is expressed as area under each of the 42 chromatogram peaks defined using the perpendicular drop method. Serum N-glyans are split into neutral (peaks 1–16) and charged (peaks 17–42).
(XLSX)

S2 Table Pairwise Correlation of samples using Pearson's Correlation Coefficient of % area under peak. (XLSX)

S1 Fig Overlaid chromatograms from A. Whole Serum N-glycan profile pooled human plasma repeated 24 times and B. Pooled human IgG glycan repeated 48 times. (PDF)

S2 Fig Chromatograms from each sample analysed. (PDF)

S3 Fig Boxplots of each individual glycan by sample type, with p values from analysis of variance. GP = Glycan peak.
(PDF)

S1 Data Reproducibility Whole plasma N-glycan chromatogram raw data. (ZIP)

S2 Data Reproducibility Whole plasma IgG chromatogram raw data. (ZIP)

S3 Data Serum Tube comparison Whole serum N-glycan raw data. (ZIP)

## References

[1] Ercan A, Cui J, Hazen MM, Batliwalla F, Royle L, Rudd PM, et al Hypogalactosylation of serum N-glycans fails to predict clinical response to methotrexate and TNF inhibition in rheumatoid arthritis. Arthritis Res. Ther.. 2012;14:R43 10.1186/ar3756 22390545 PMC3446410

[2] Ercan A, Cui J, Chatterton DEW, Deane KD, Hazen MM, Brintnell W, et al Aberrant IgG galactosylation precedes disease onset, correlates with disease activity, and is prevalent in autoantibodies in rheumatoid arthritis. Arthritis Rheum. 2010;62:2239–2248. 10.1002/art.27533 20506563 PMC4118465

[3] Novokmet M, Lukić E, Vučković F, Ðurić Ž, Keser T, Rajšl K, et al Changes in IgG and total plasma protein glycomes in acute systemic inflammation. Sci Rep. 2014;4:1–10. 10.1038/srep04347 PMC3949295 24614541

[4] Gornik O, Lauc G. Glycosylation of serum proteins in inflammatory diseases. Dis Markers. 2008;25:267–278. 19126970 10.1155/2008/493289 PMC3827815

[5] Collins ES, Galligan MC, Saldova R, Adamczyk B, Abrahams JL, Campbell MP, et al Glycosylation status of serum in inflammatory arthritis in response to anti-TNF treatment. Rheumatol (United Kingdom). 2013;52:1572–1582. 10.1093/rheumatology/ket189 23681398

[6] Chen C, Schmilovitz-Weiss H, Liu X, Pappo O, Halpern M, Sulkes J, et al Serum protein N-glycans profiling for the discovery of potential biomarkers for nonalcoholic steatohepatitis. J Proteome Res. 2009;8:463–470. 10.1021/pr800656e 19140676

[7] Miyahara K, Nouso K, Saito S, Hiraoka S, Harada K, Takahashi S, et al Serum glycan markers for evaluation of disease activity and prediction of clinical course in patients with ulcerative colitis. PLoS One. 2013;8:e74861 10.1371/journal.pone.0074861 24116015 PMC3792068

[8] Shinzaki S, Iijima H, Nakagawa T, Egawa S, Nakajima S, Ishii S, et al IgG oligosaccharide alterations are a novel diagnostic marker for disease activity and the clinical course of inflammatory bowel disease. Am J Gastroenterol. 2008;103:1173–1181. 10.1111/j.1572-0241.2007.01699.x 18177457

[9] Bowen RAR, Hortin GL, Csako G, Otañez OH, Remaley AT. Impact of blood collection devices on clinical chemistry assays. Clin. Biochem.. 2010;43:4–25. 10.1016/j.clinbiochem.2009.10.001 19822139

[10] Cuhadar S, Atay A, Koseoglu M, Dirican A, Hur A. Stability studies of common biochemical analytes in serum separator tubes with or without gel barrier subjected to various storage conditions. Biochem medica. 2012;22:202–214. 22838186 10.11613/bm.2012.023 PMC4062343

[11] Shi RZ, van Rossum HH, Bowen RAR. Serum testosterone quantitation by liquid chromatography-tandem mass spectrometry: Interference from blood collection tubes. Clin Biochem. 2012;45:1706–1709. 10.1016/j.clinbiochem.2012.08.008 22971570

[12] Drake SK, Bowen RAR, Remaley AT, Hortin GL. Potential interferences from blood collection tubes in mass spectrometric analyses of serum polypeptides. Clin Chem. 2004;50:2398–2401. 15563493 10.1373/clinchem.2004.040303

[13] Dasgupta A, Dean R, Saldana S, Kinnaman G, McLawhon RW. Absorption of therapeutic drugs by barrier gels in serum separator blood collection tubes. Volume- and time-dependent reduction in total and free drug concentrations. Am J Clin Pathol. 1994;101:456–461. 8160636 10.1093/ajcp/101.4.456

[14] Dasgupta A, Yared MA, Wells A. Time-dependent absorption of therapeutic drugs by the gel of the Greiner Vacuette blood collection tube. Ther Drug Monit. 2000;22:427–431. 10942183 10.1097/00007691-200008000-00011

[15] Hsieh S-Y, Chen R-K, Pan Y, Lee H-L. Systematical evaluation of the effects of sample collection procedures on low-molecular-weight serum/plasma proteome profiling. Proteomics. 2006;6:3189–3198. 16586434 10.1002/pmic.200500535

[16] Zolg JW, Langen H. How industry is approaching the search for new diagnostic markers and biomarkers. Mol Cell Proteomics. 2004;3:345–354. 14749446 10.1074/mcp.M400007-MCP200

[17] Royle L, Campbell MP, Radcliffe CM, White DM, Harvey DJ, Abrahams JL, et al HPLC-based analysis of serum N-glycans on a 96-well plate platform with dedicated database software. Anal Biochem. 2008;376:1–12. 10.1016/j.ab.2007.12.012 18194658

[18] Huffman JE, Pučić-Baković M, Klarić L, Hennig R, Selman MHJ, Vučković F, et al Comparative performance of four methods for high-throughput glycosylation analysis of immunoglobulin G in genetic and epidemiological research. Mol Cell Proteomics. 2014;13:1598–1610. 10.1074/mcp.M113.037465 24719452 PMC4047478

[19] Sharratt CL, Gilbert CJ, Cornes MC, Ford C, Gama R. EDTA sample contamination is common and often undetected, putting patients at unnecessary risk of harm. Int J Clin Pract. 2009;63:1259–1262. 10.1111/j.1742-1241.2008.01981.x 19624792

[20] Hanson BA. ChemoSpec: Exploratory Chemometrics for Spectroscopy version 2.0–2. 2014.

[21] Herrmann E, Martin Maechler. lokern: Kernel Regression Smoothing with Local or Global Plug-in Bandwidth version 1.1–5. 2013.

[22] Guile GR, Rudd PM, Wing DR, Prime SB, Dwek RA. A rapid high-resolution high-performance liquid chromatographic method for separating glycan mixtures and analyzing oligosaccharide profiles. Anal Biochem. 1996;240:210–226. 8811911 10.1006/abio.1996.0351

[23] Knezević A, Polasek O, Gornik O, Rudan I, Campbell H, Hayward C, et al Variability, heritability and environmental determinants of human plasma N-glycome. J Proteome Res. 2009;8:694–701. 10.1021/pr800737u 19035662

[24] Gornik O, Wagner J, Pucić M, Knezević A, Redzic I, Lauc G. Stability of N-glycan profiles in human plasma. Glycobiology. 2009;19:1547–1553. 10.1093/glycob/cwp134 19726492

[25] Pucic M, Knezevic A, Vidic J, Adamczyk B, Novokmet M, Polasek O, et al High Throughput Isolation and Glycosylation Analysis of IgG-Variability and Heritability of the IgG Glycome in Three Isolated Human Populations. Mol. Cell. Proteomics.. 2011;10:M111.010090–M111.010090. 10.1074/mcp.M111.010090 21653738 PMC3205872

[26] Parekh RB, Dwek RA, Sutton BJ, Fernandes DL, Leung A, Stanworth D, et al Association of rheumatoid arthritis and primary osteoarthritis with changes in the glycosylation pattern of total serum IgG. Nature. 1985;316:452–457. 3927174 10.1038/316452a0

[27] Reiding KR, Blank D, Kuijper DM, Deelder AM, Wuhrer M. High-throughput profiling of protein N-glycosylation by MALDI-TOF-MS employing linkage-specific sialic acid esterification. Anal Chem. 2014;86:5784–5793. 10.1021/ac500335t 24831253

[28] Harvey DJ, Royle L, Radcliffe CM, Rudd PM, Dwek RA. Structural and quantitative analysis of N-linked glycans by matrix-assisted laser desorption ionization and negative ion nanospray mass spectrometry. Anal Biochem. 2008;376:44–60. 10.1016/j.ab.2008.01.025 18294950

[29] Pucic M, Pinto S, Novokmet M, Knezevic A, Gornik O, Polasek O, et al Common aberrations from the normal human plasma N-glycan profile. Glycobiology. 2010;20:970–975. 10.1093/glycob/cwq052 20378934

