# Correction: Changes to Serum Sample Tube and Processing Methodology Does Not Cause Inter-Individual Variation in Automated Whole Serum N-Glycan Profiling in Health and Disease

**DOI:** 10.1371/journal.pone.0129335

**Published:** 2015-06-01

**Authors:** Nicholas T. Ventham, Richard A. Gardner, Nicholas A. Kennedy, Archana Shubhakar, Rahul Kalla, Elaine R. Nimmo, IBD-BIOM Consortium, Daryl L. Fernandes, Jack Satsangi, Daniel I. R. Spencer

There is an error in the title of this article: "Inter-Individual" should be "Intra-Individual." The correct title is: Changes to Serum Sample Tube and Processing Methodology Does Not Cause Intra-Individual Variation in Automated Whole Serum N-Glycan Profiling in Health and Disease.

The correct citation is: Ventham NT, Gardner RA, Kennedy NA, Shubhakar A, Kalla R, Nimmo ER, et al. (2015) Changes to Serum Sample Tube and Processing Methodology Does Not Cause Intra-Individual Variation in Automated Whole Serum N-Glycan Profiling in Health and Disease. PLoS ONE 10(4): e0123028. doi:10.1371/journal.pone.0123028.

There is also an error in the caption for Fig 2: "glucose homopolymer (GHP)" should be "glycans." Please see the correct caption here.

**Fig 2:**
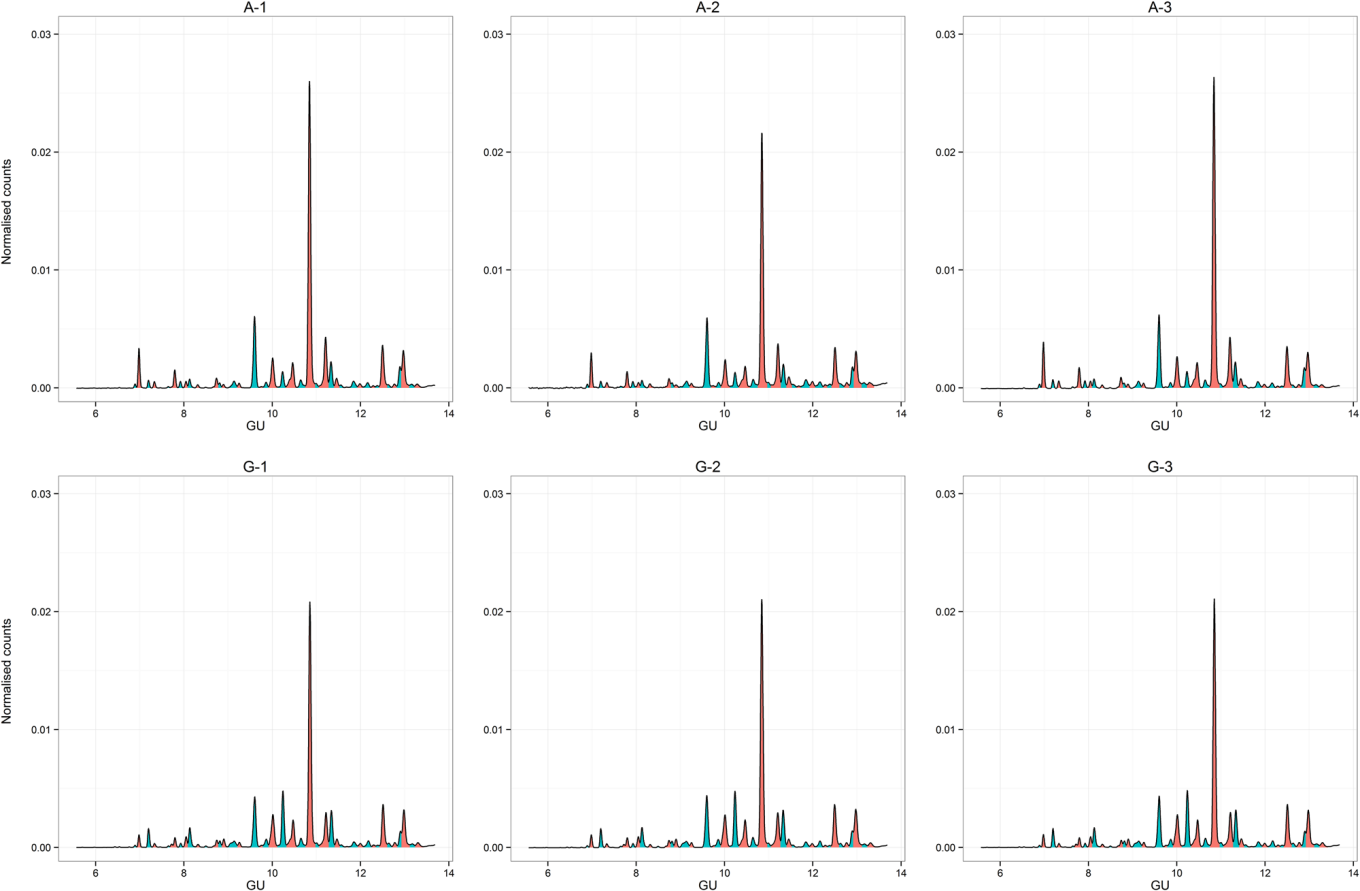
Fluorescence chromatogram showing a 2-aminobenzamide labeled glycans run on a UHPLC HILIC column. Fig 2A–2C and 2D–2F are derived from different patients respectively. Peaks are labeled arbitrarily in order from 1 to 42, with glucose unit values according to Guile et al [22] Peaks are colored alternately to aid identification.
