# Supplementary figures and images for: Changes to Serum Sample Tube and Processing Methodology Does Not Cause Inter-Individual Variation in Automated Whole Serum N-Glycan Profiling in Health and Disease
Source: PLoS One. 2015 Apr 1;10(4):e0123028. doi: 10.1371/journal.pone.0123028 (PMC4382121; doi:10.1371/journal.pone.0123028)

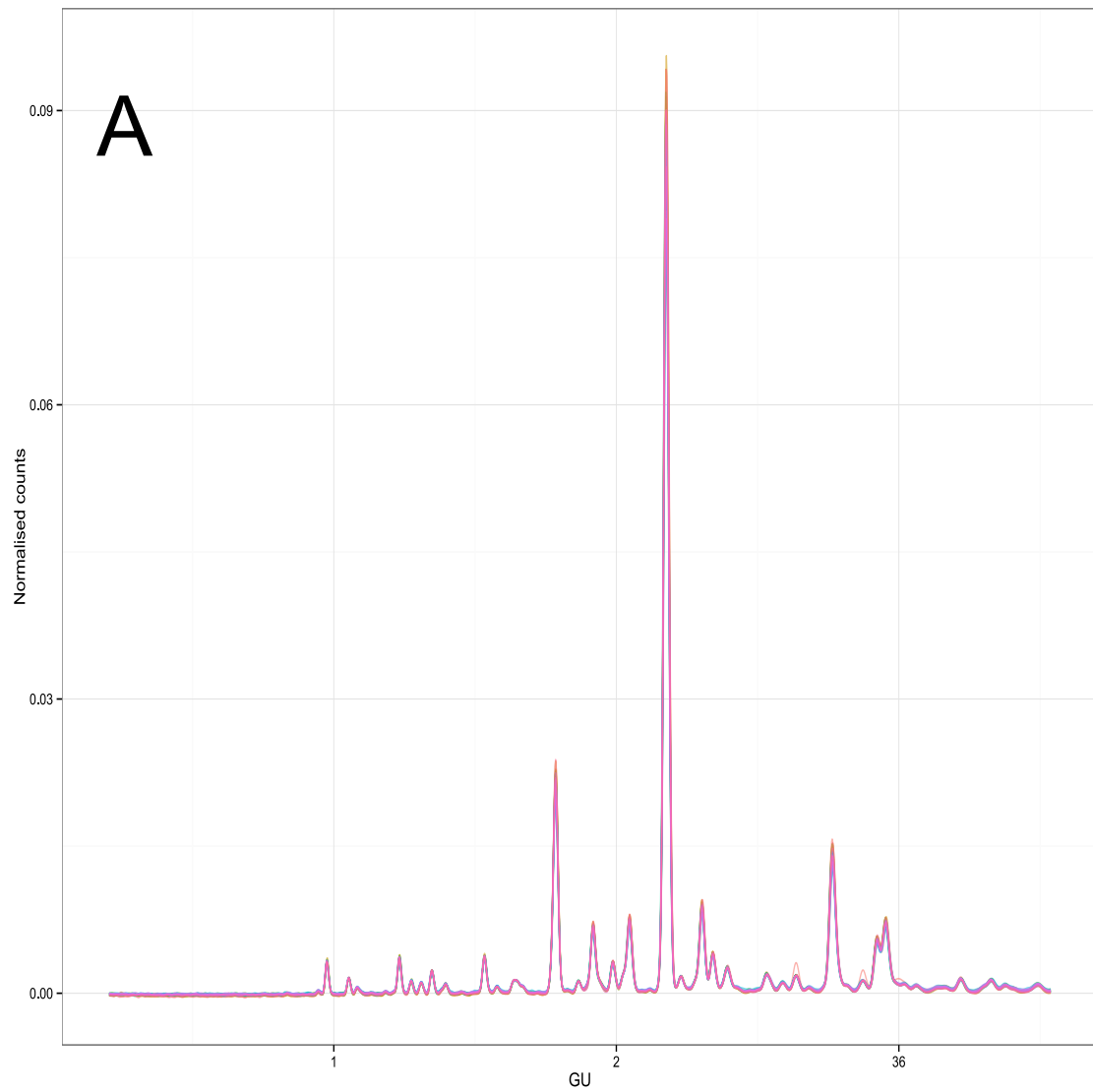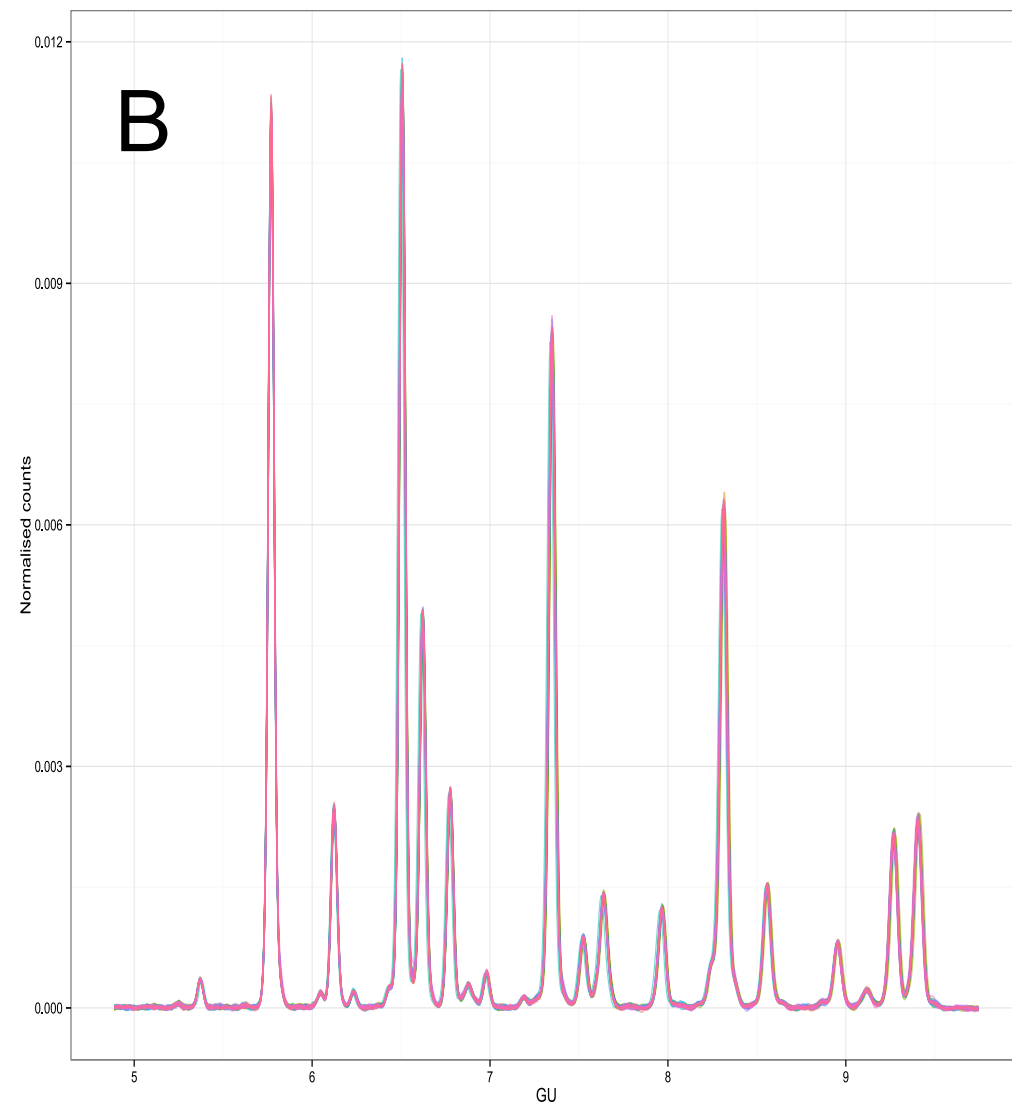

Supplement: S1 Fig — (PDF) [file pone.0123028.s003.pdf]

A-1

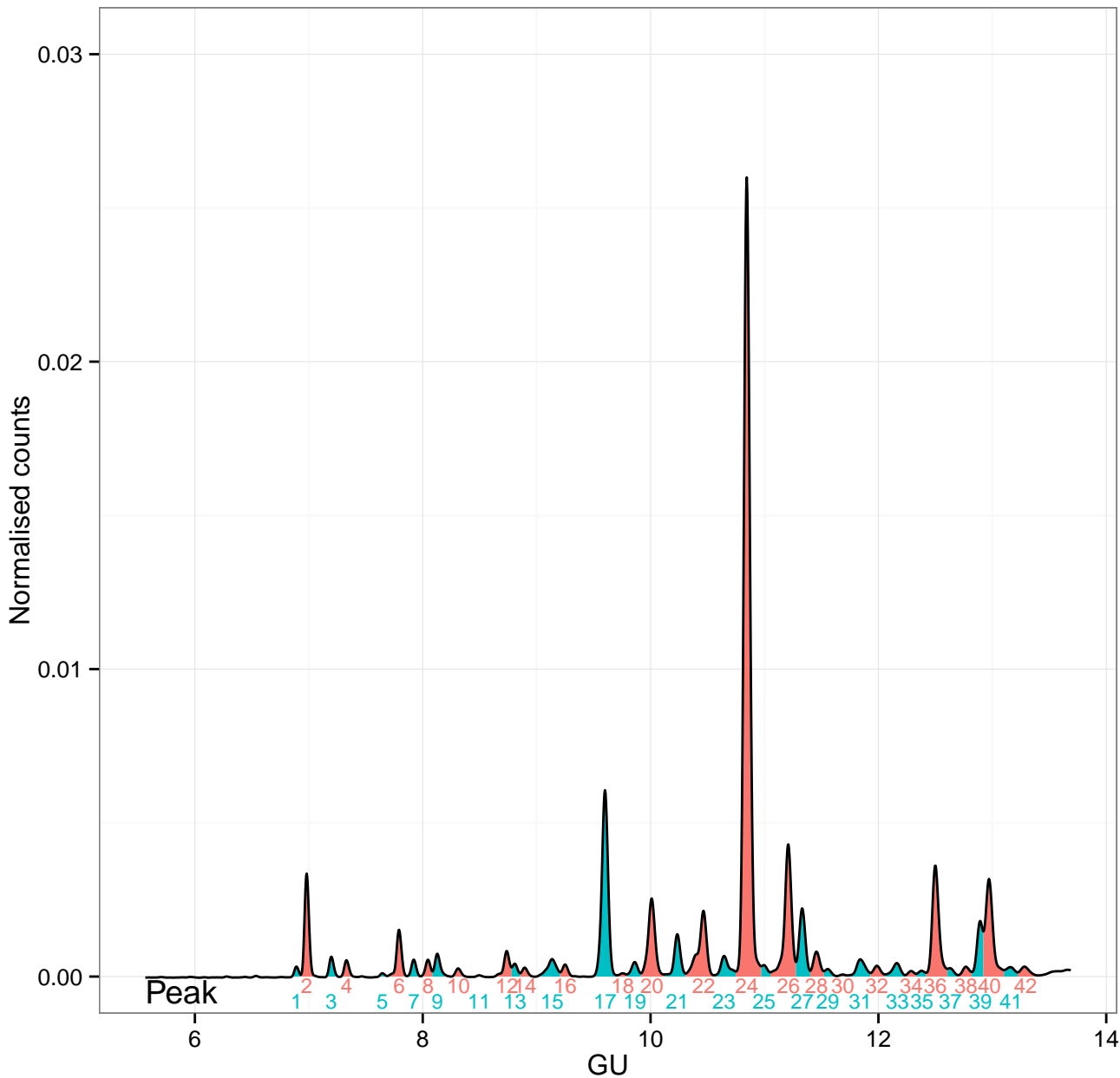

A-2

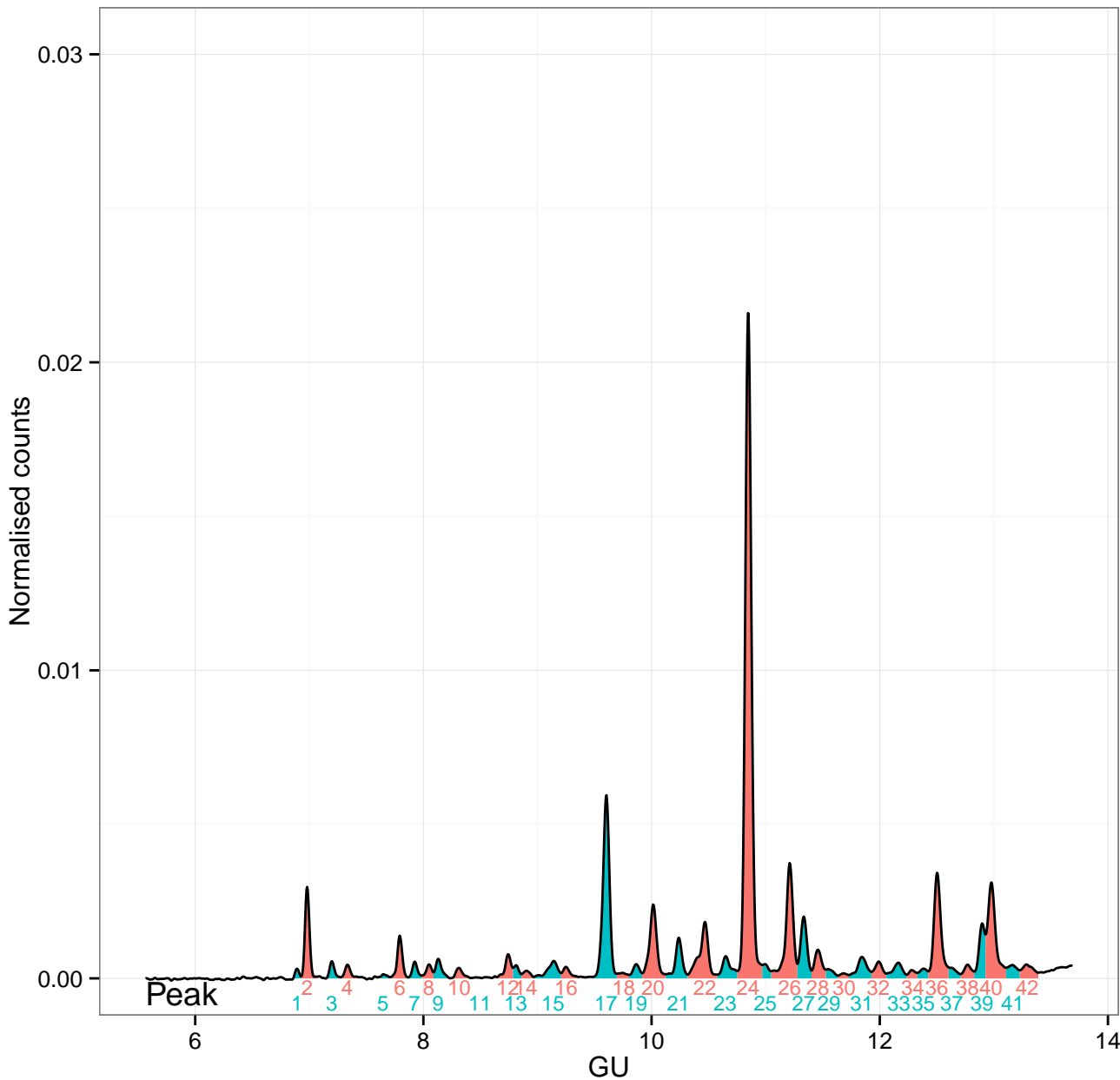

A-3

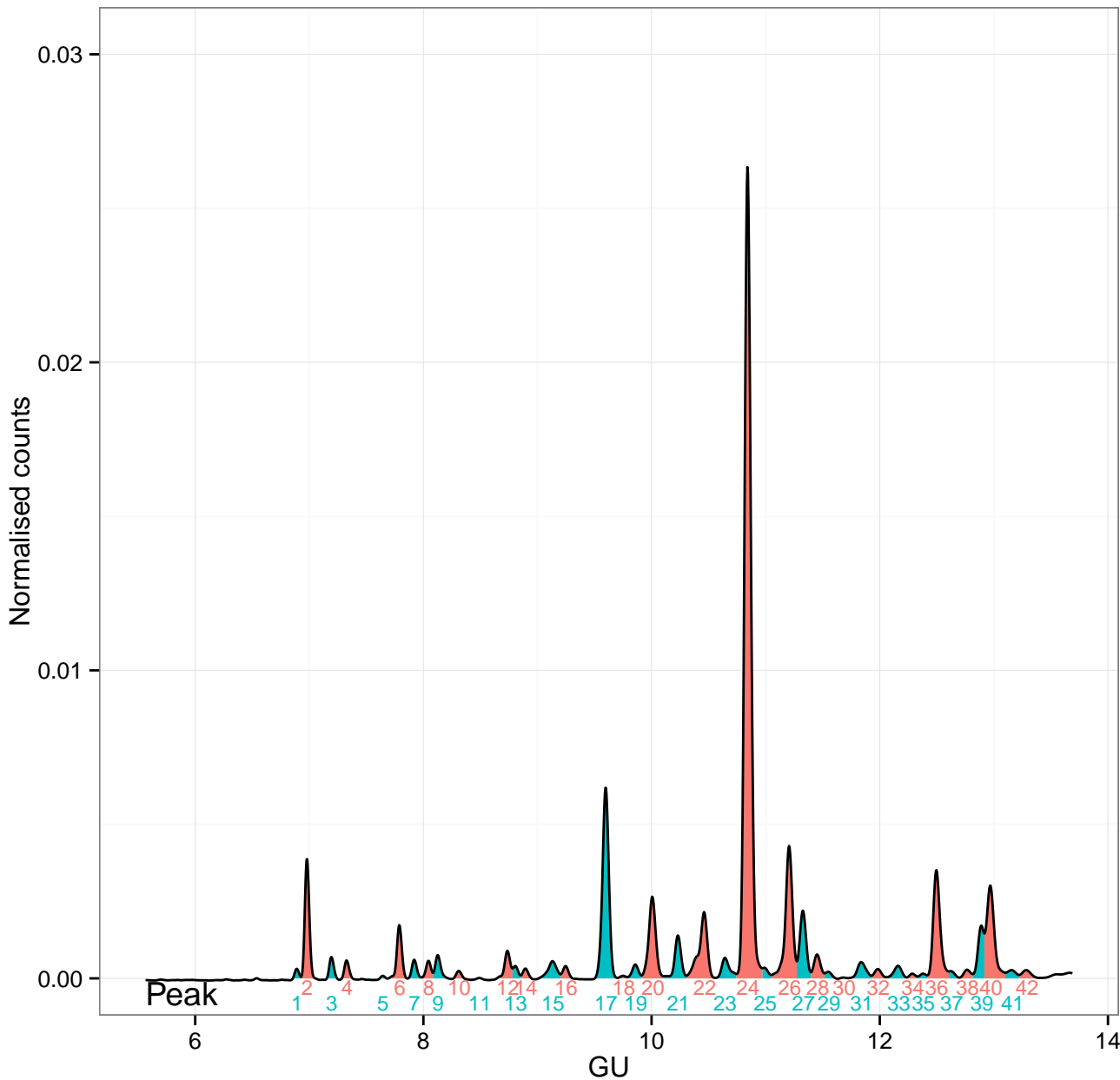

B-1

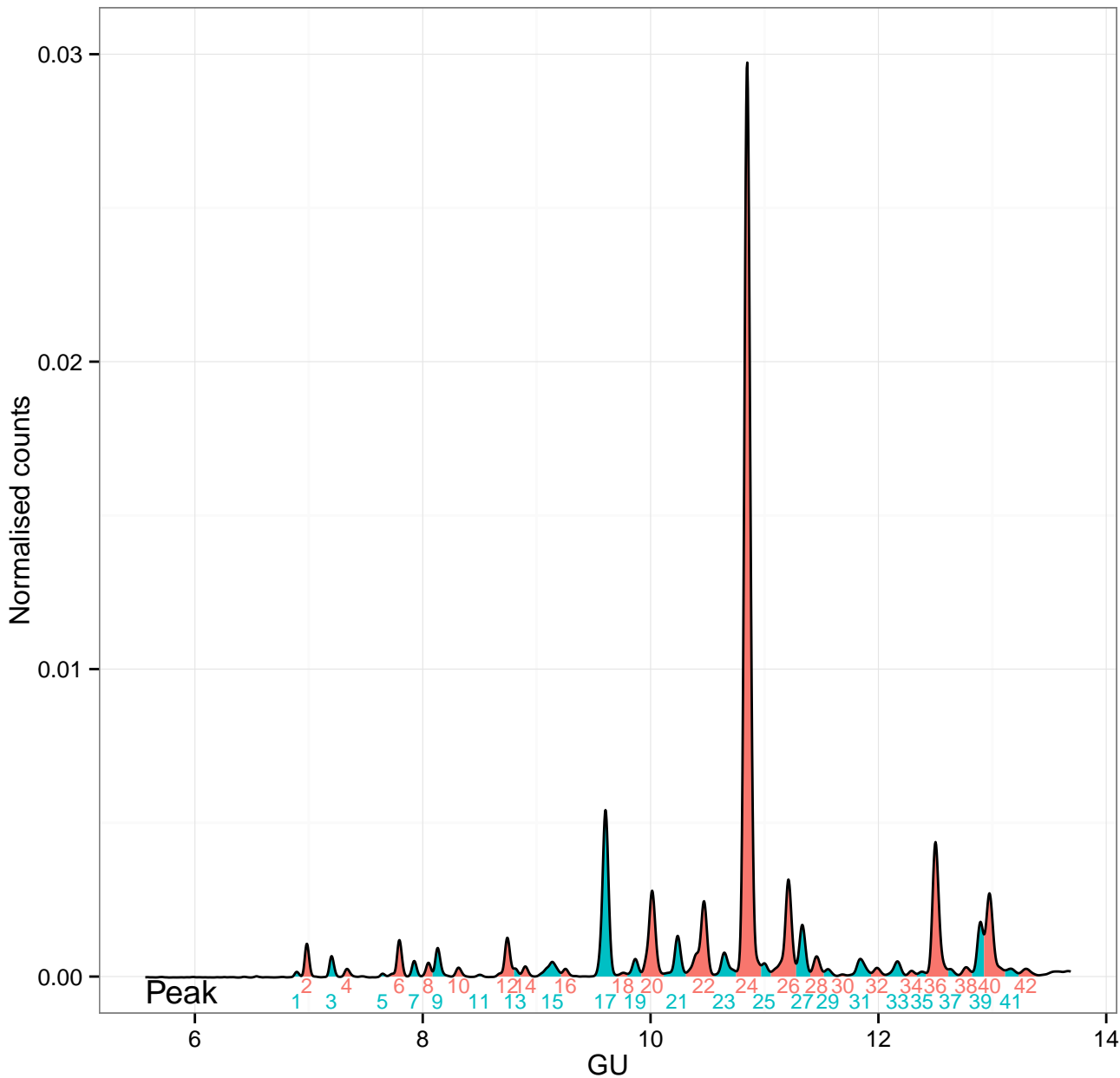

B-2

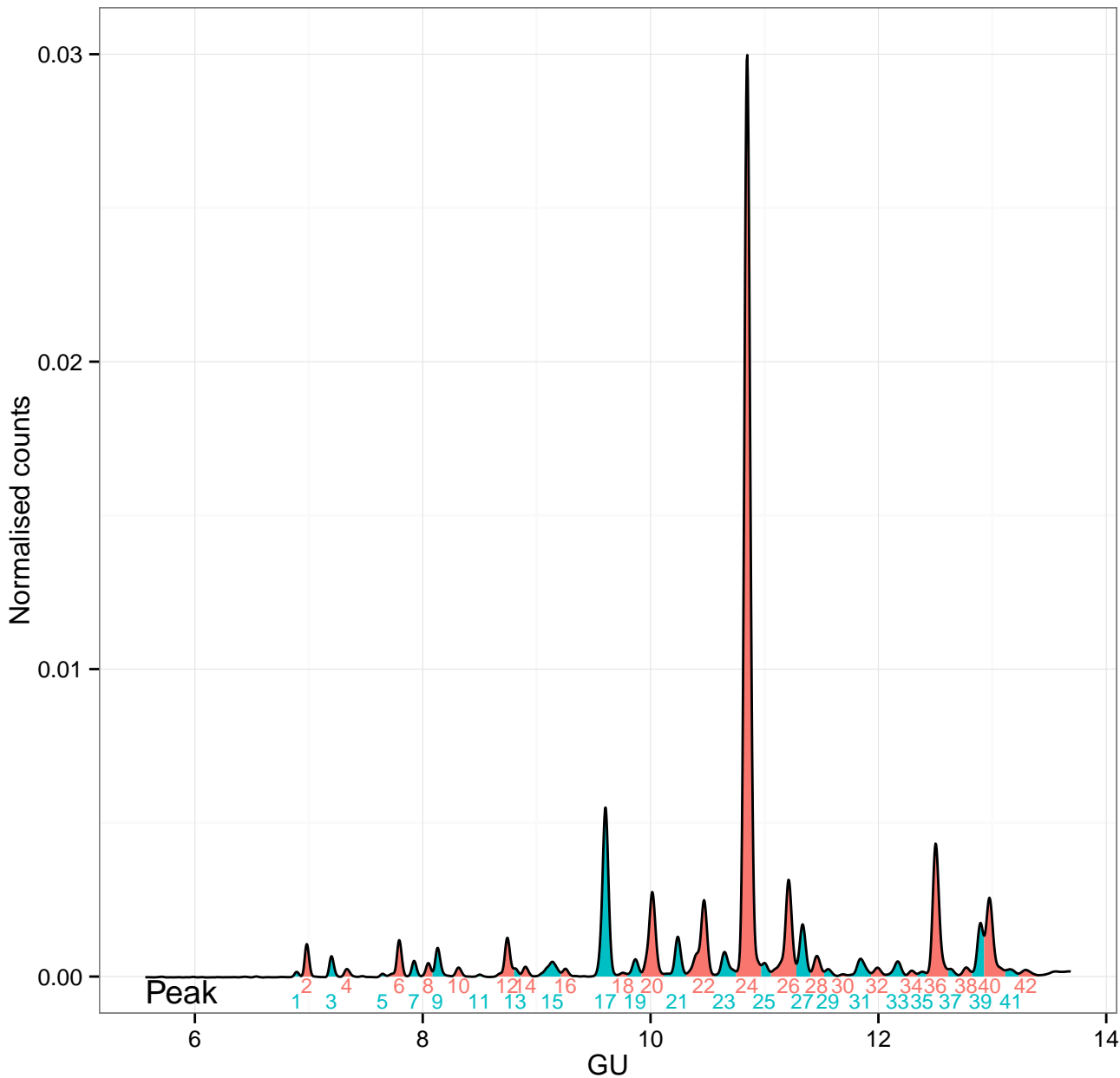

B-3

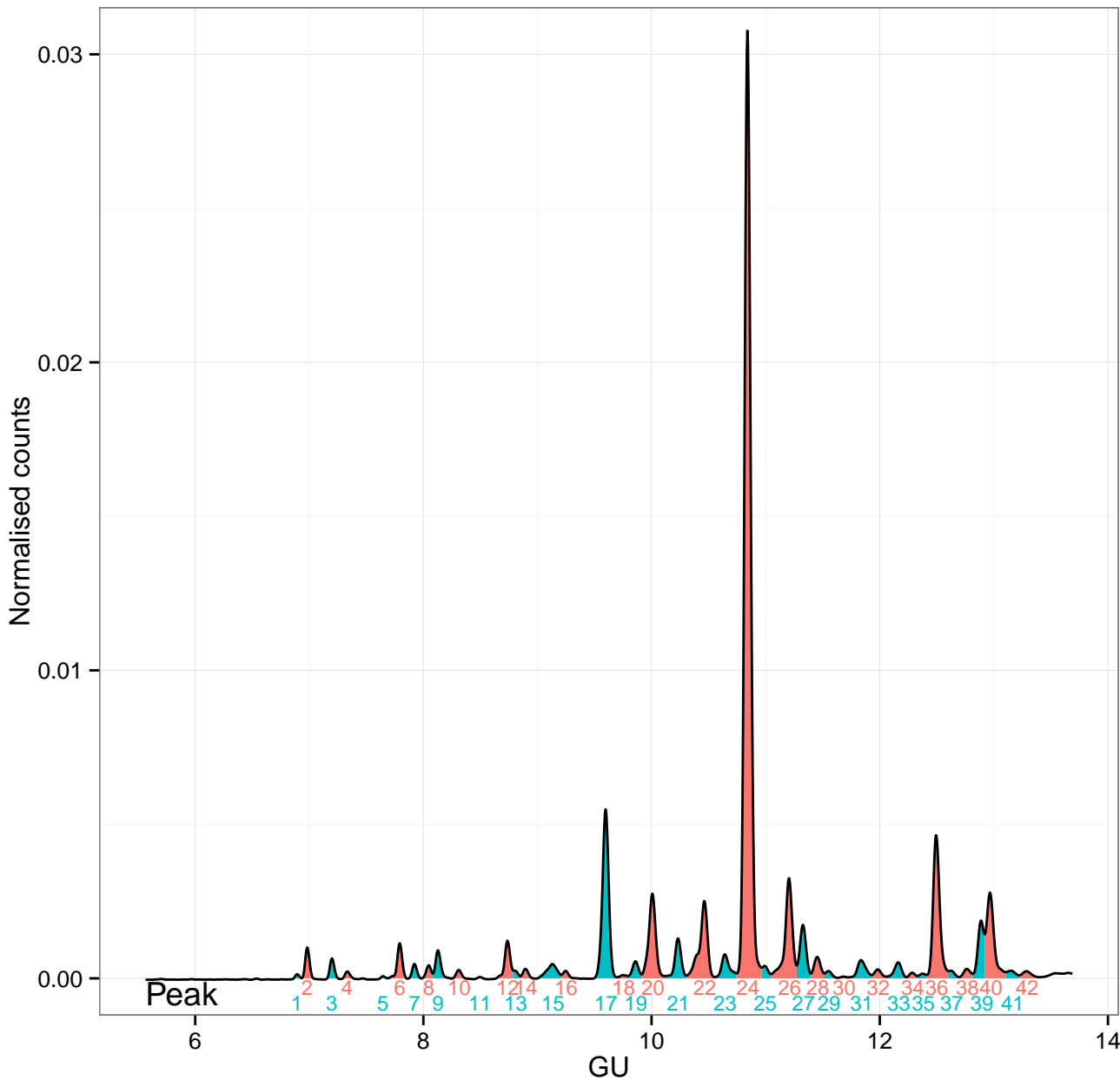

C-1

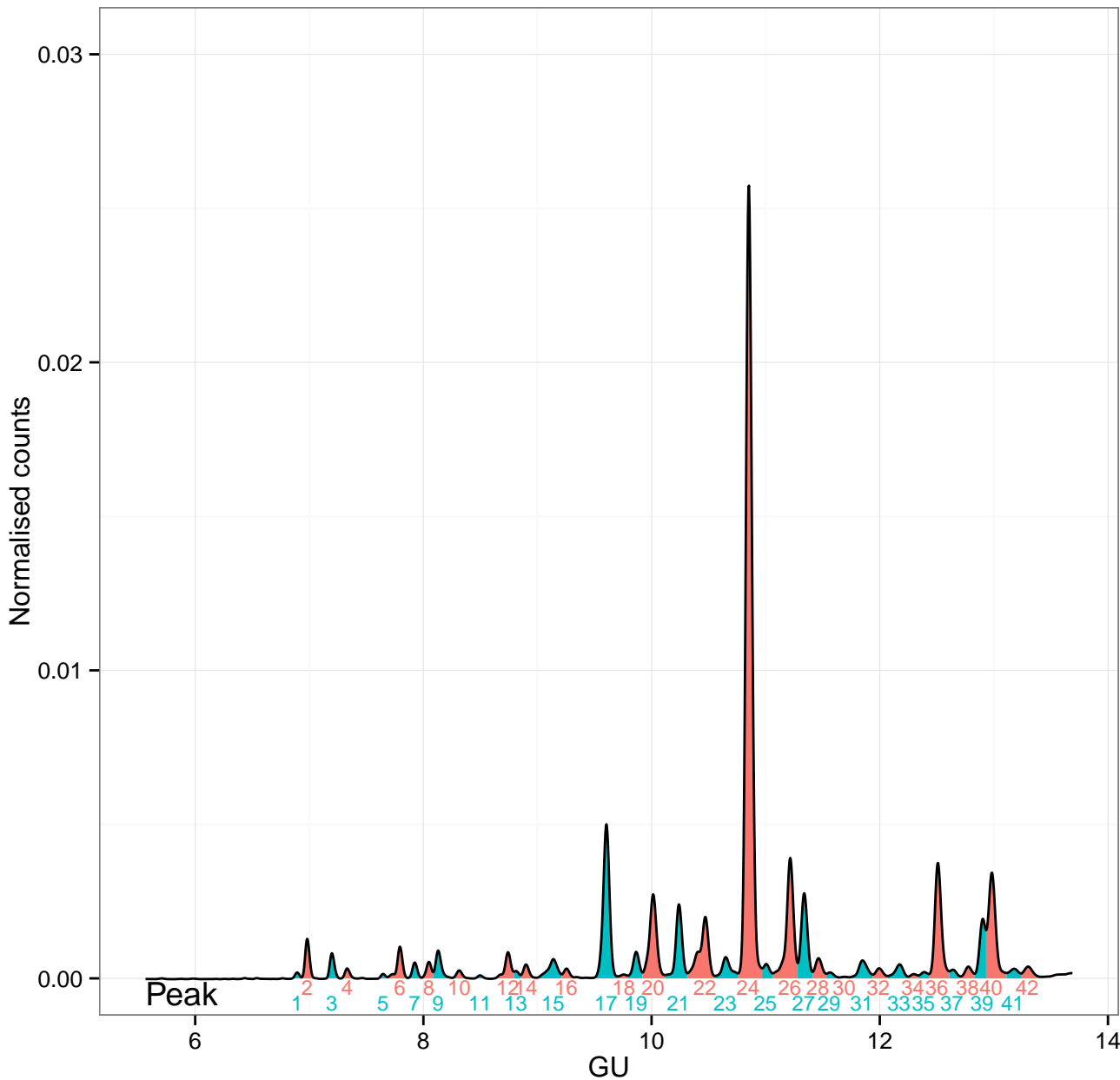

C-2

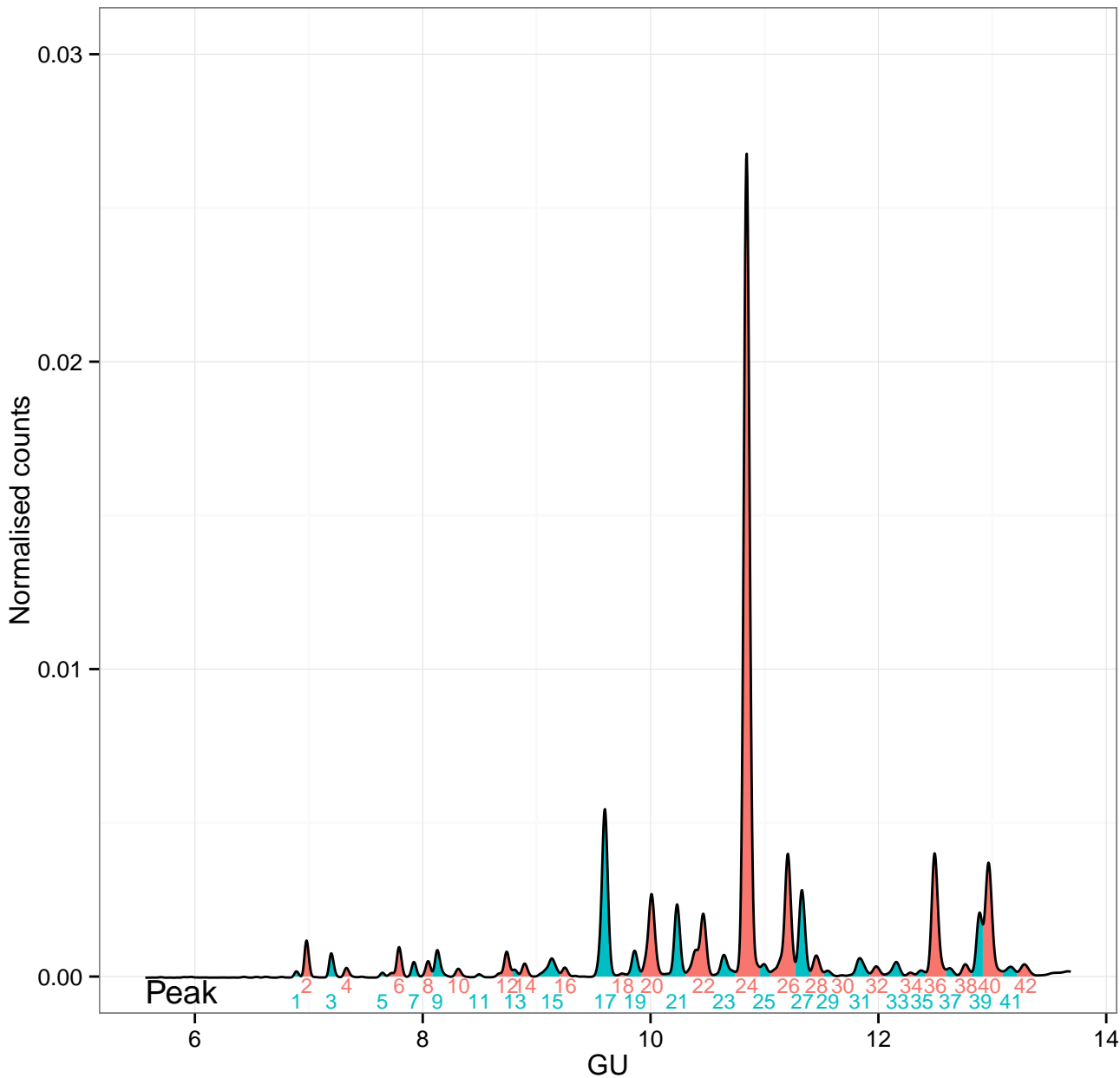

C-3

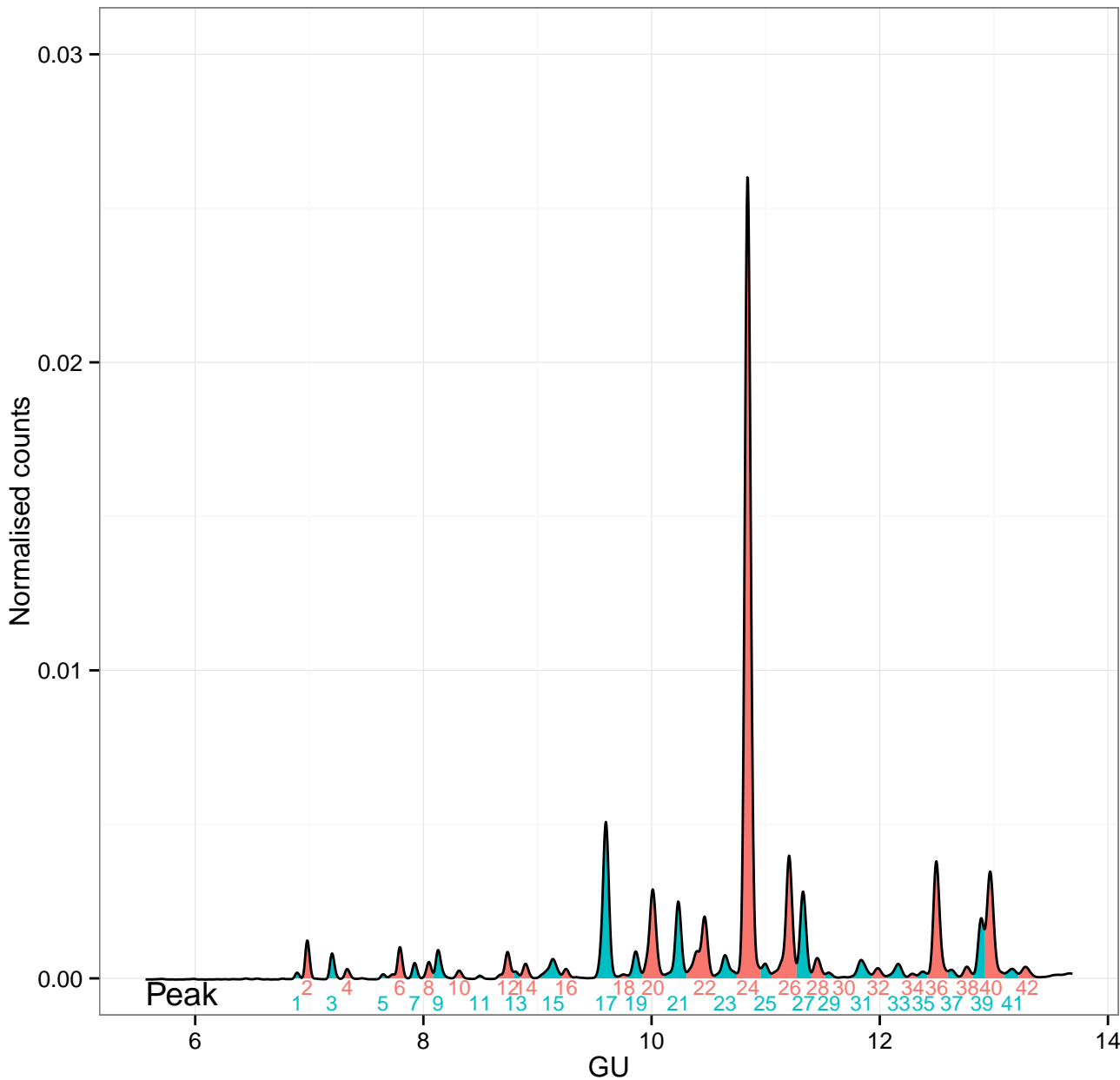

D-1

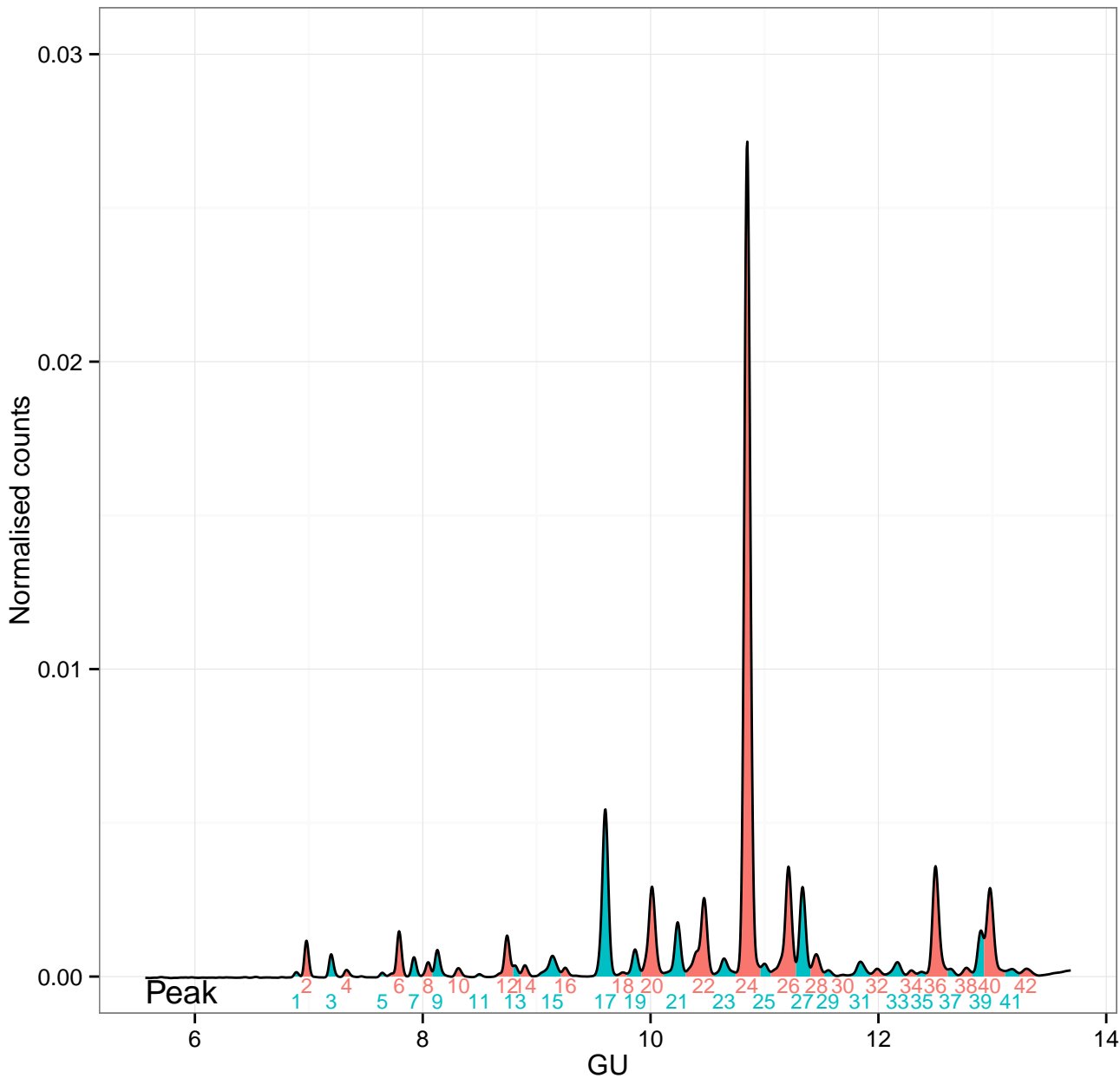

D-2

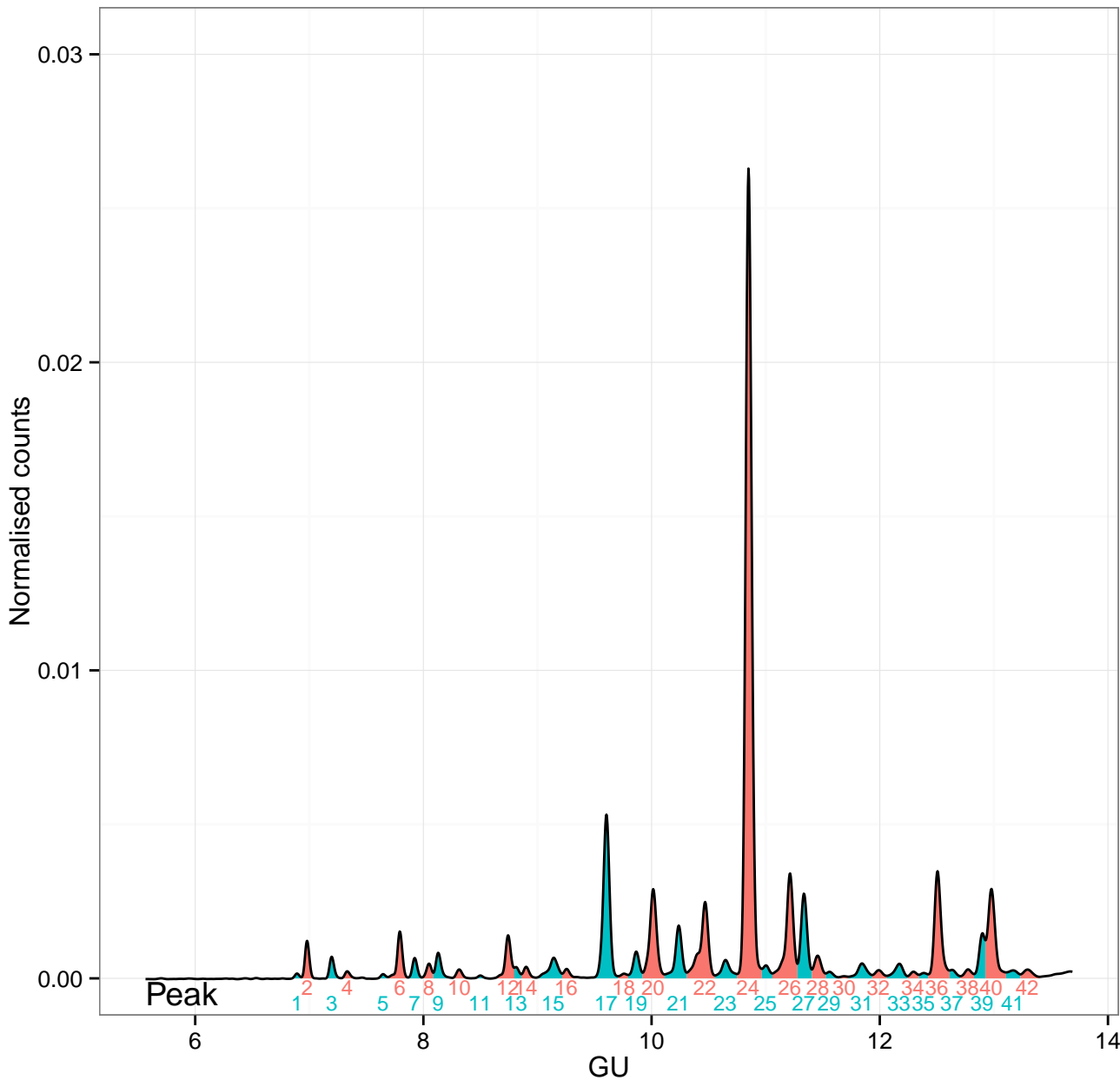

D-3

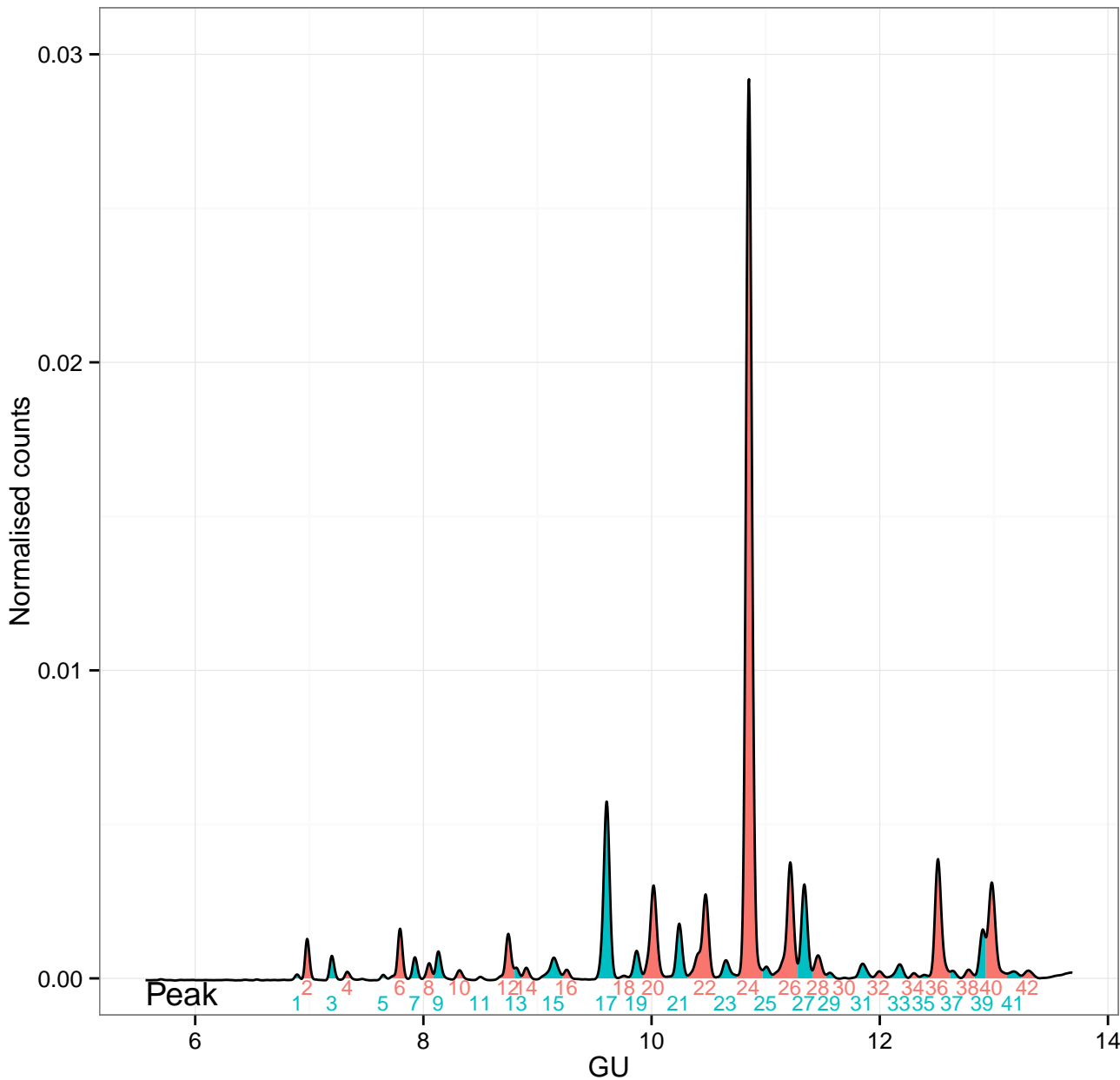

E-1

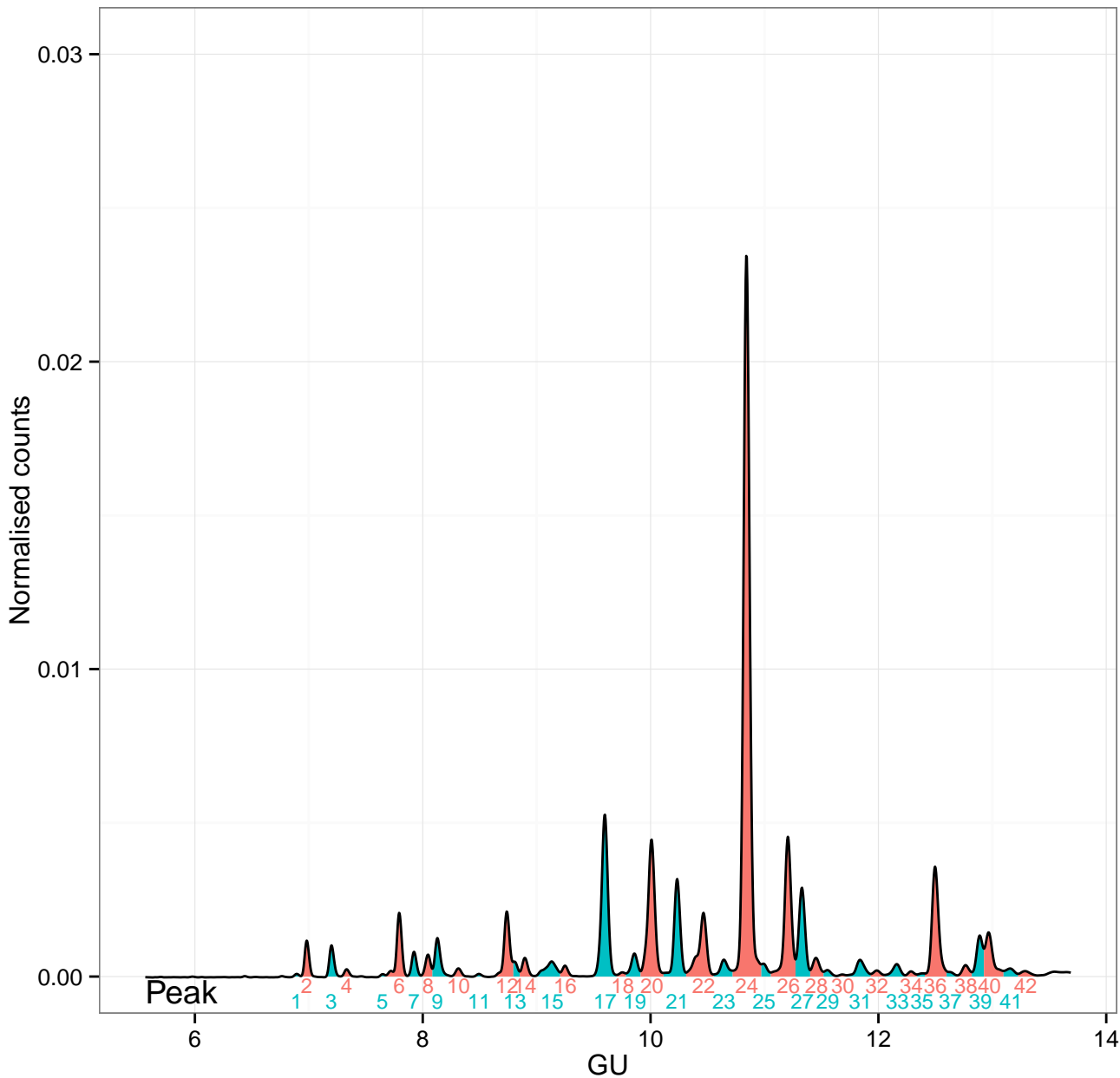

E-3

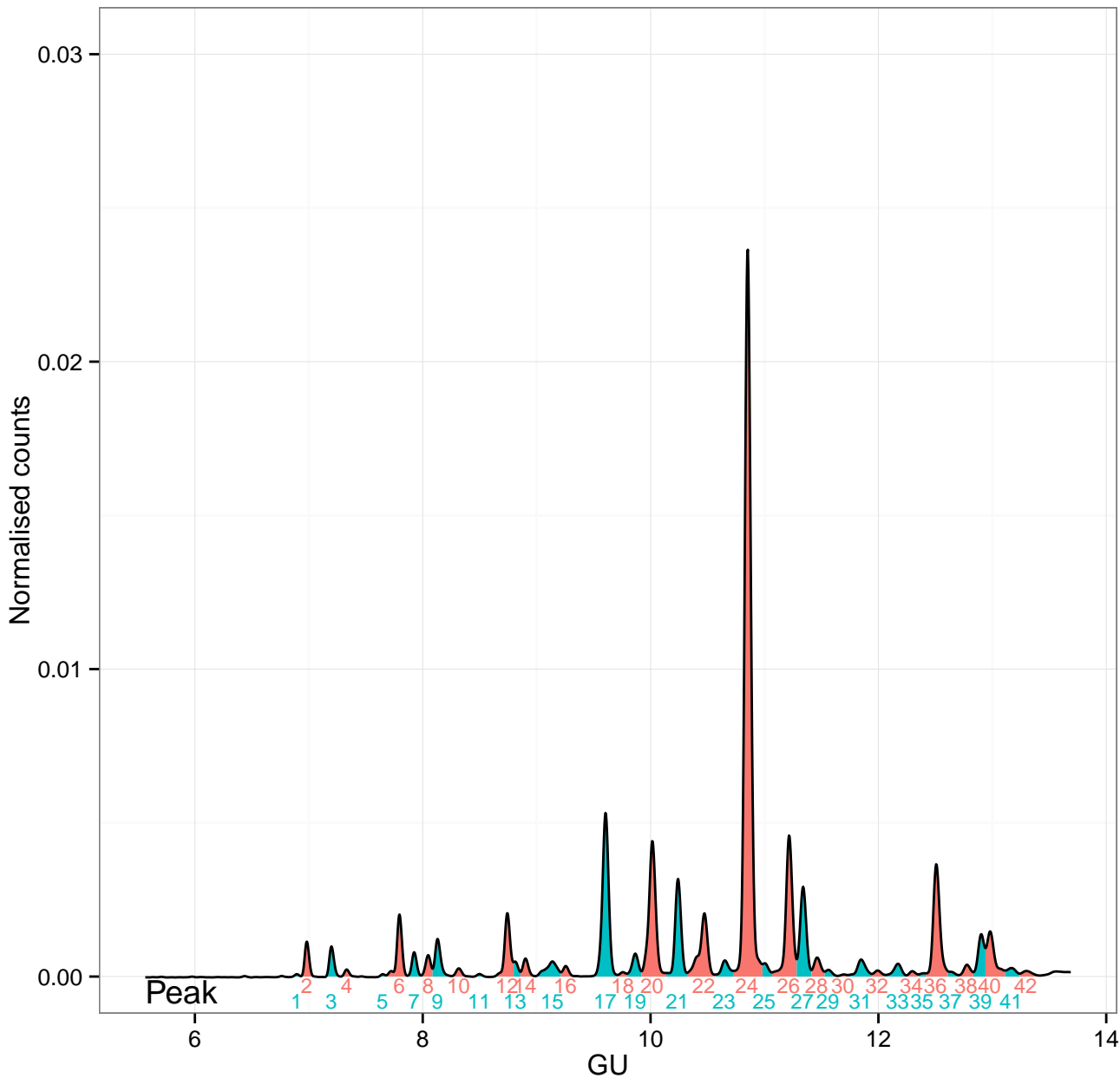

F-1

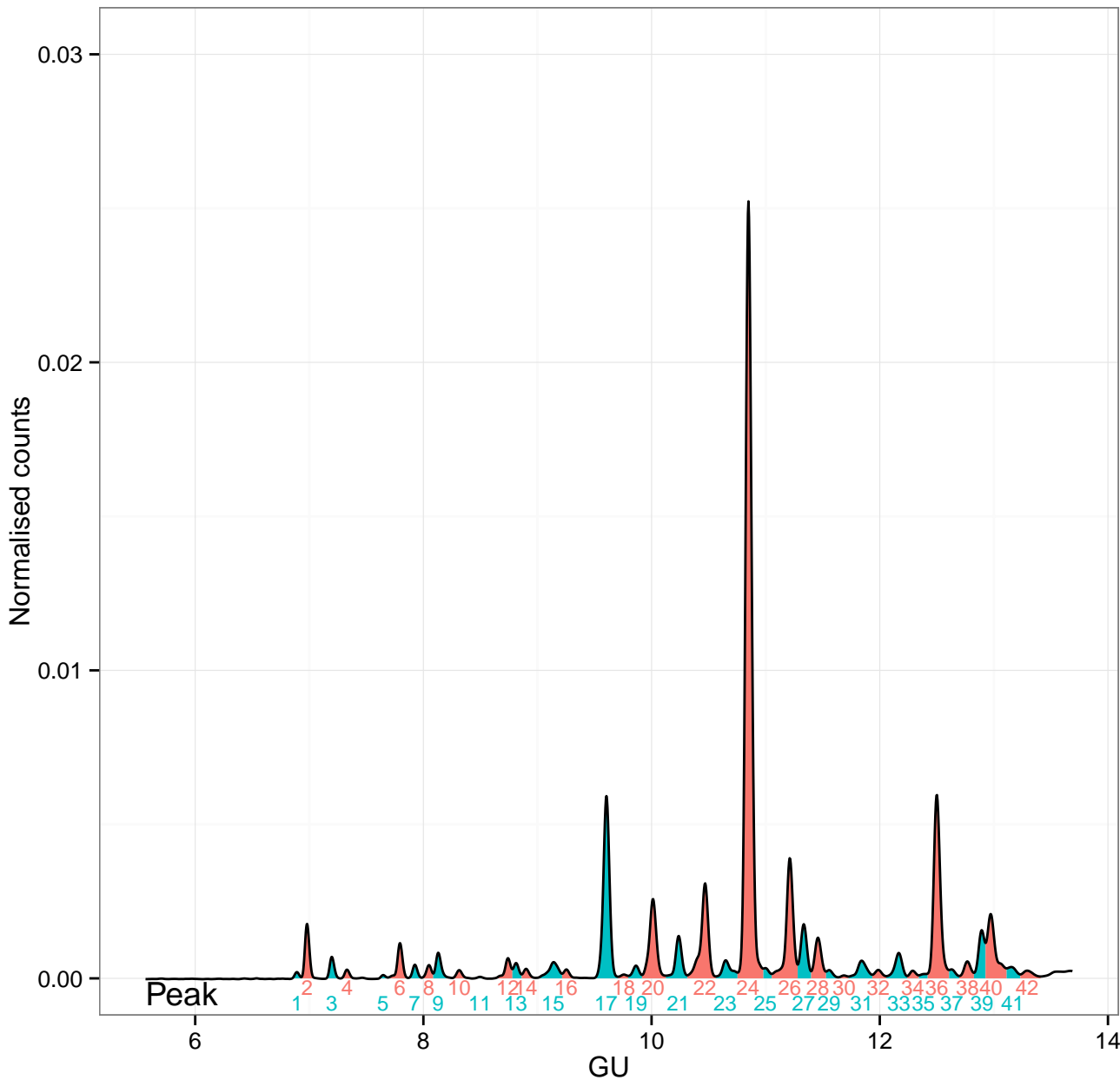

F-3

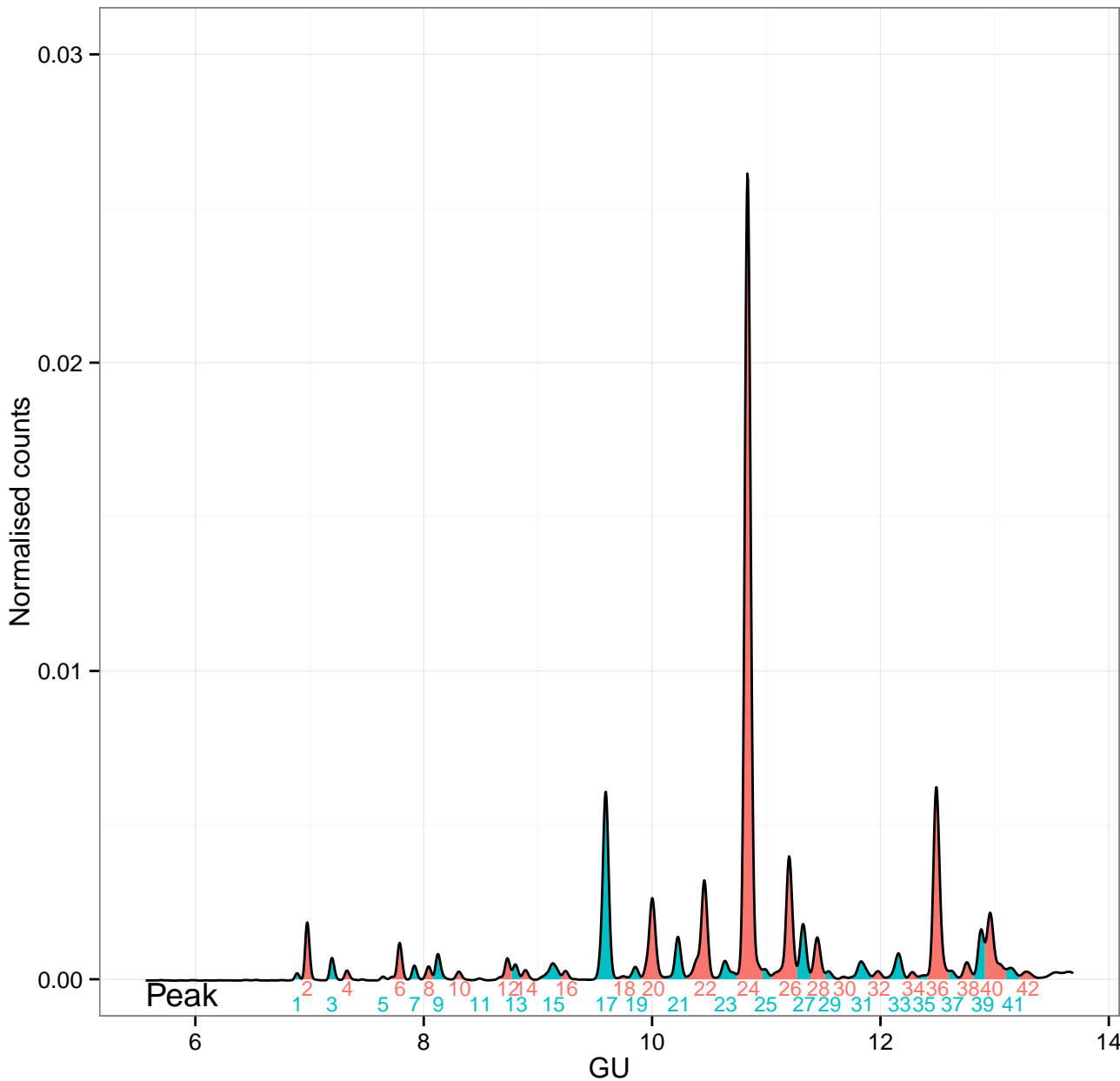

G-1

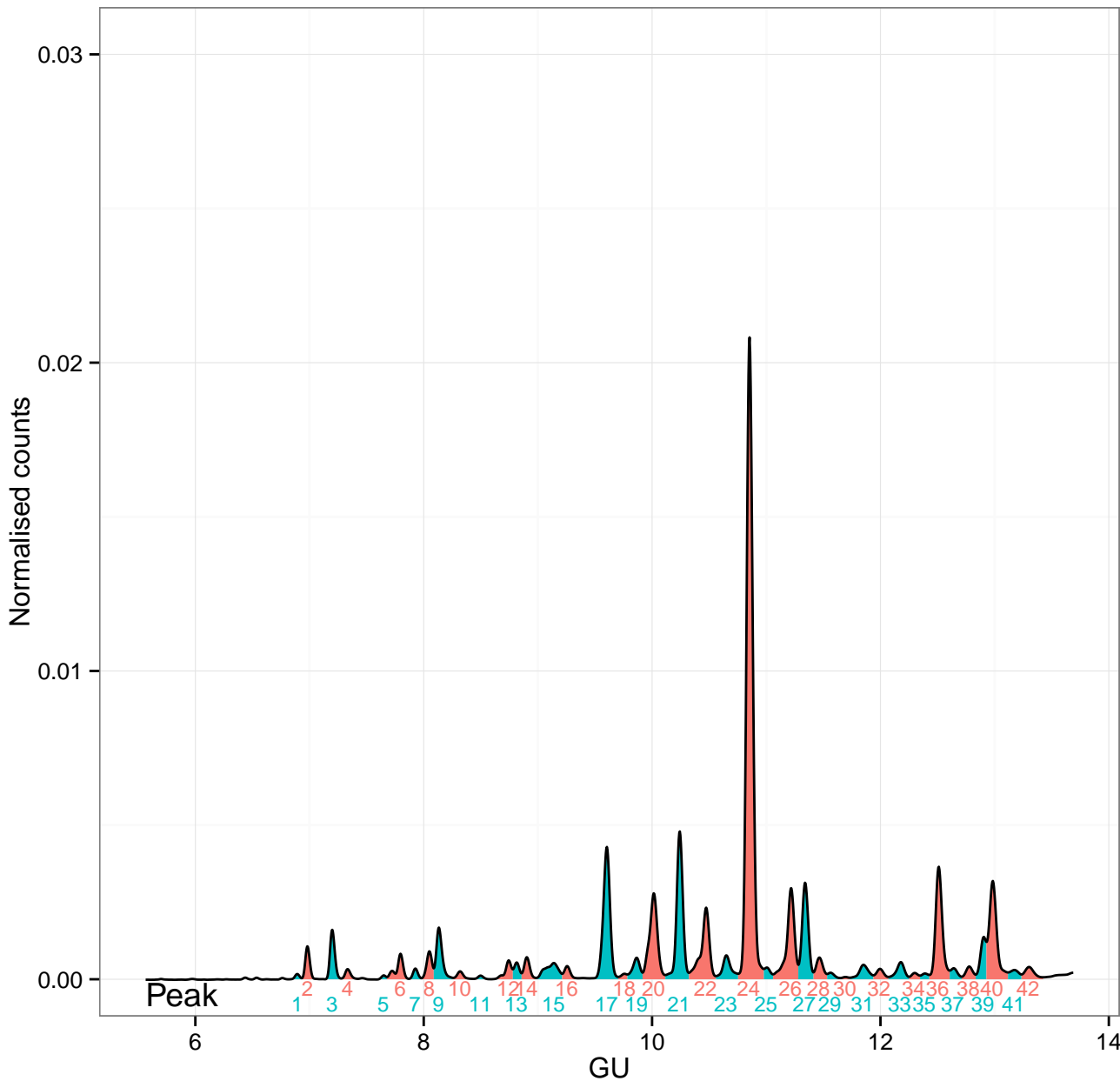

G-2

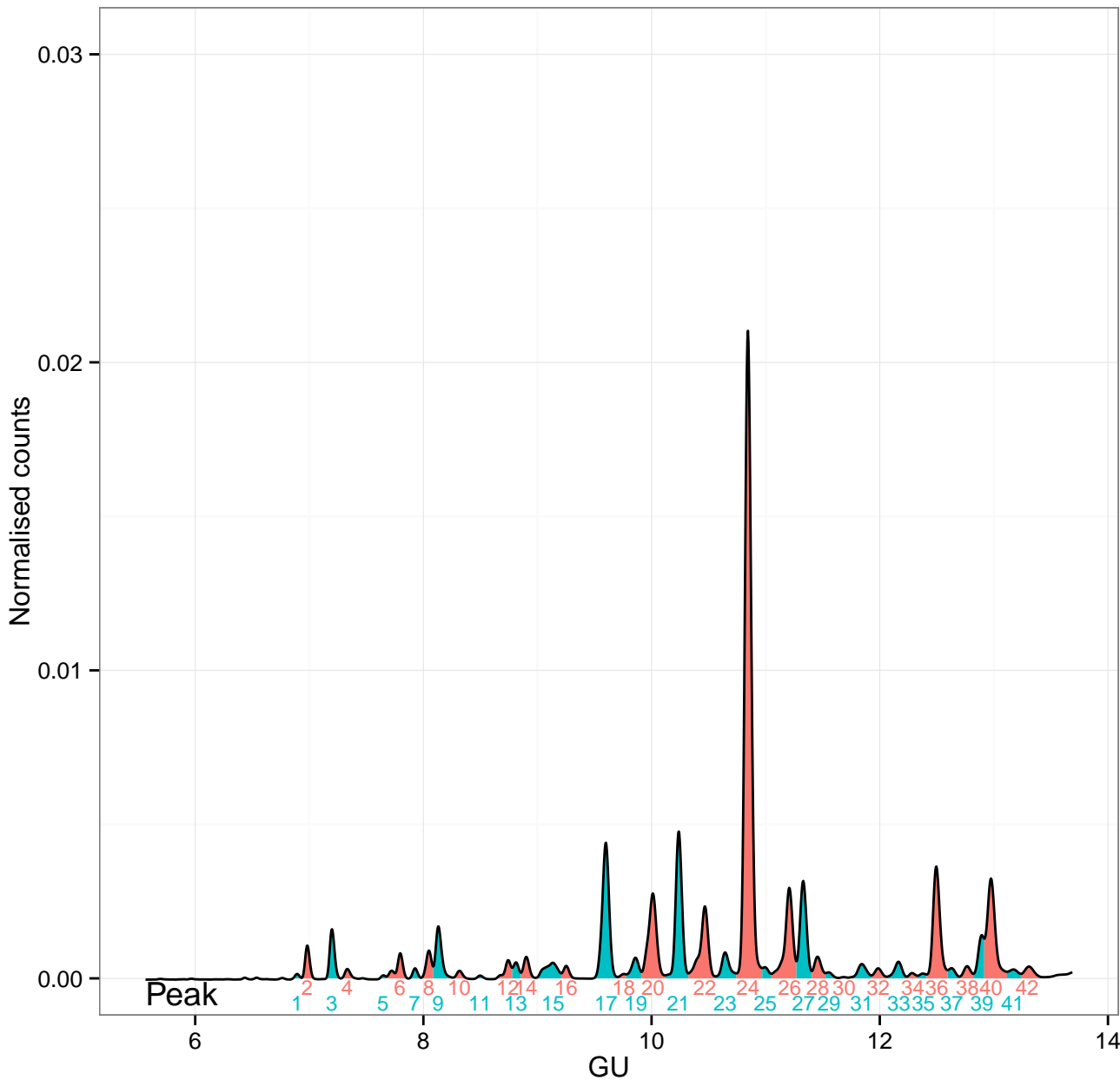

G-3

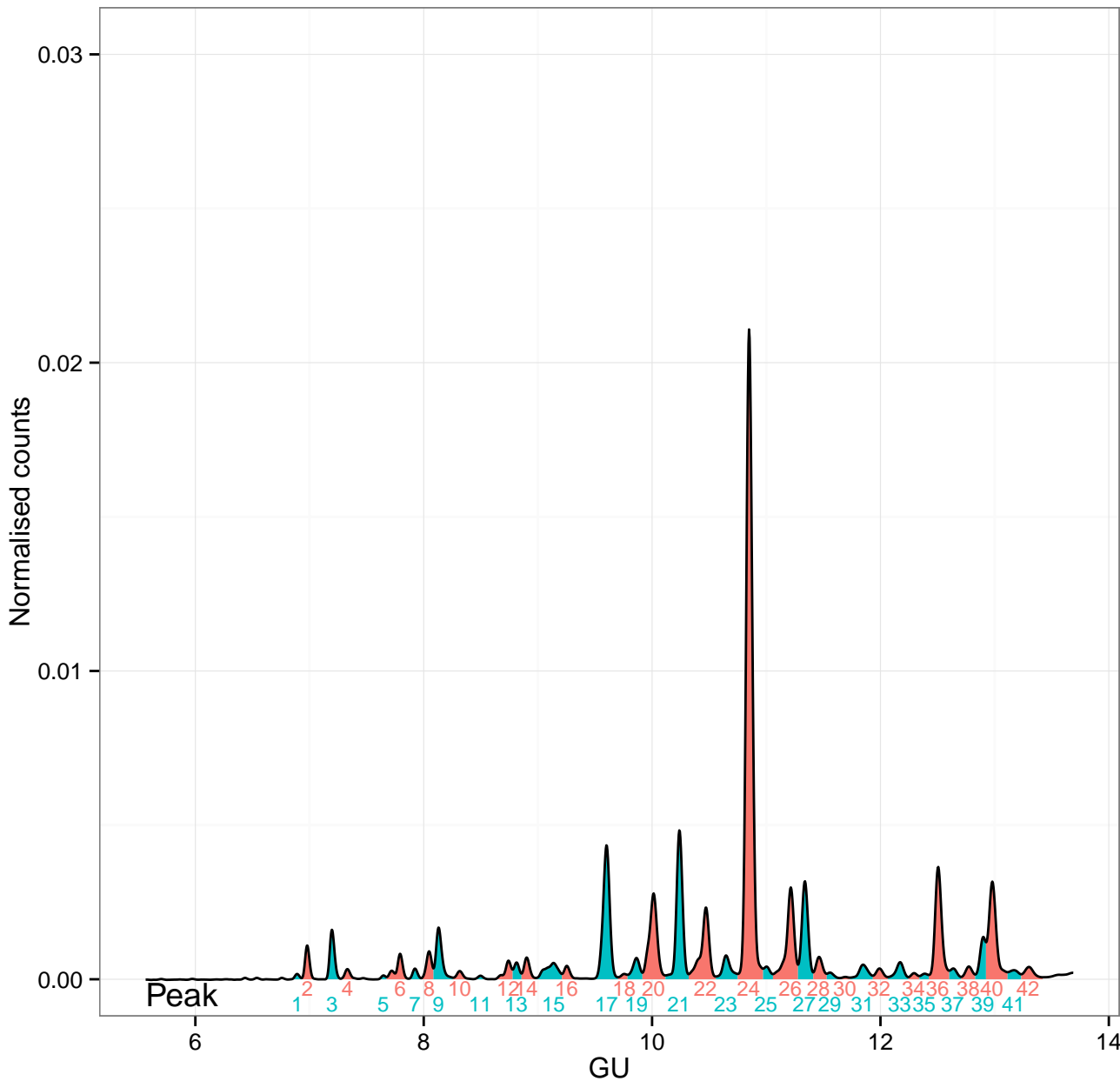

H-1

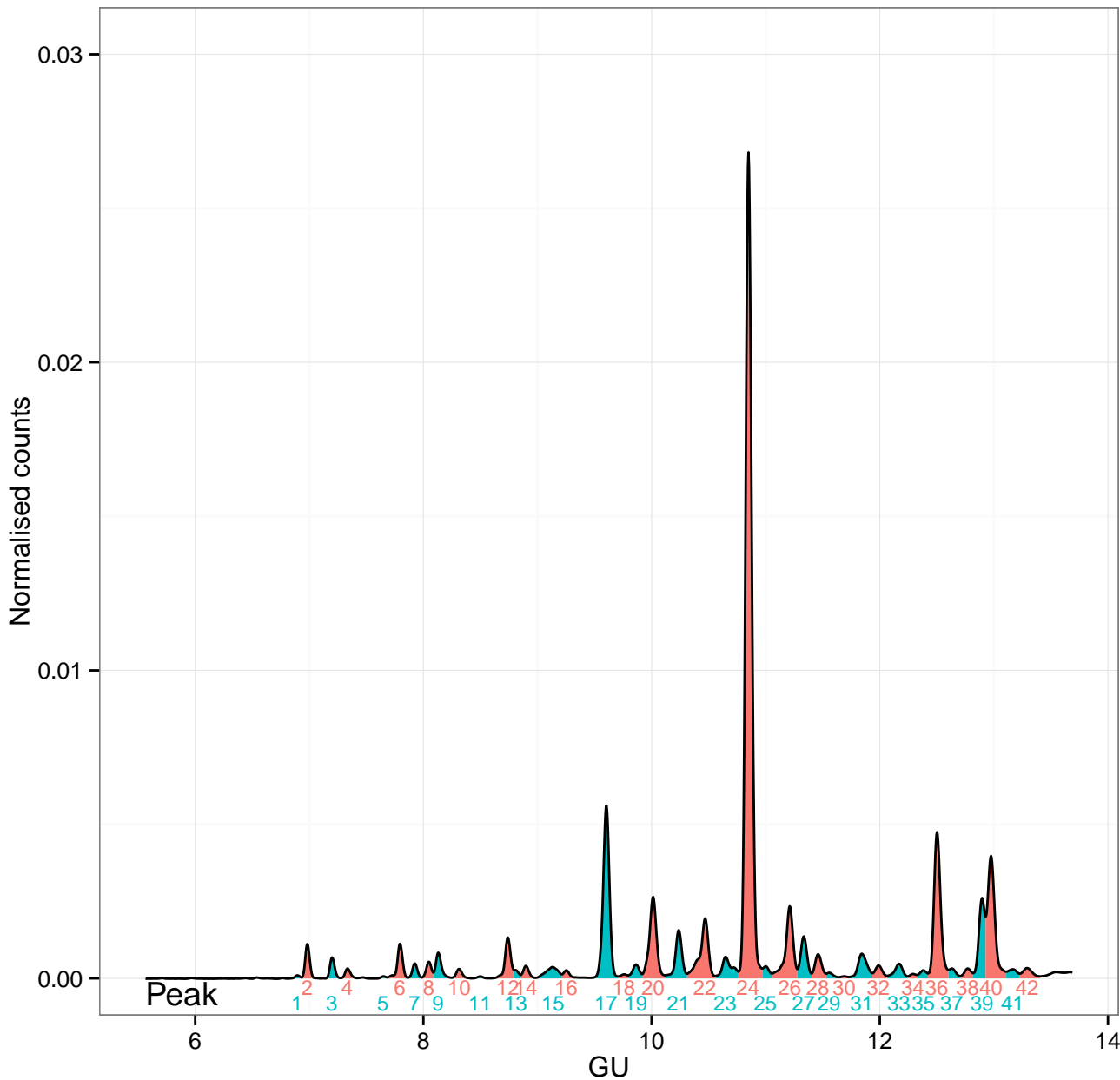

## H-2

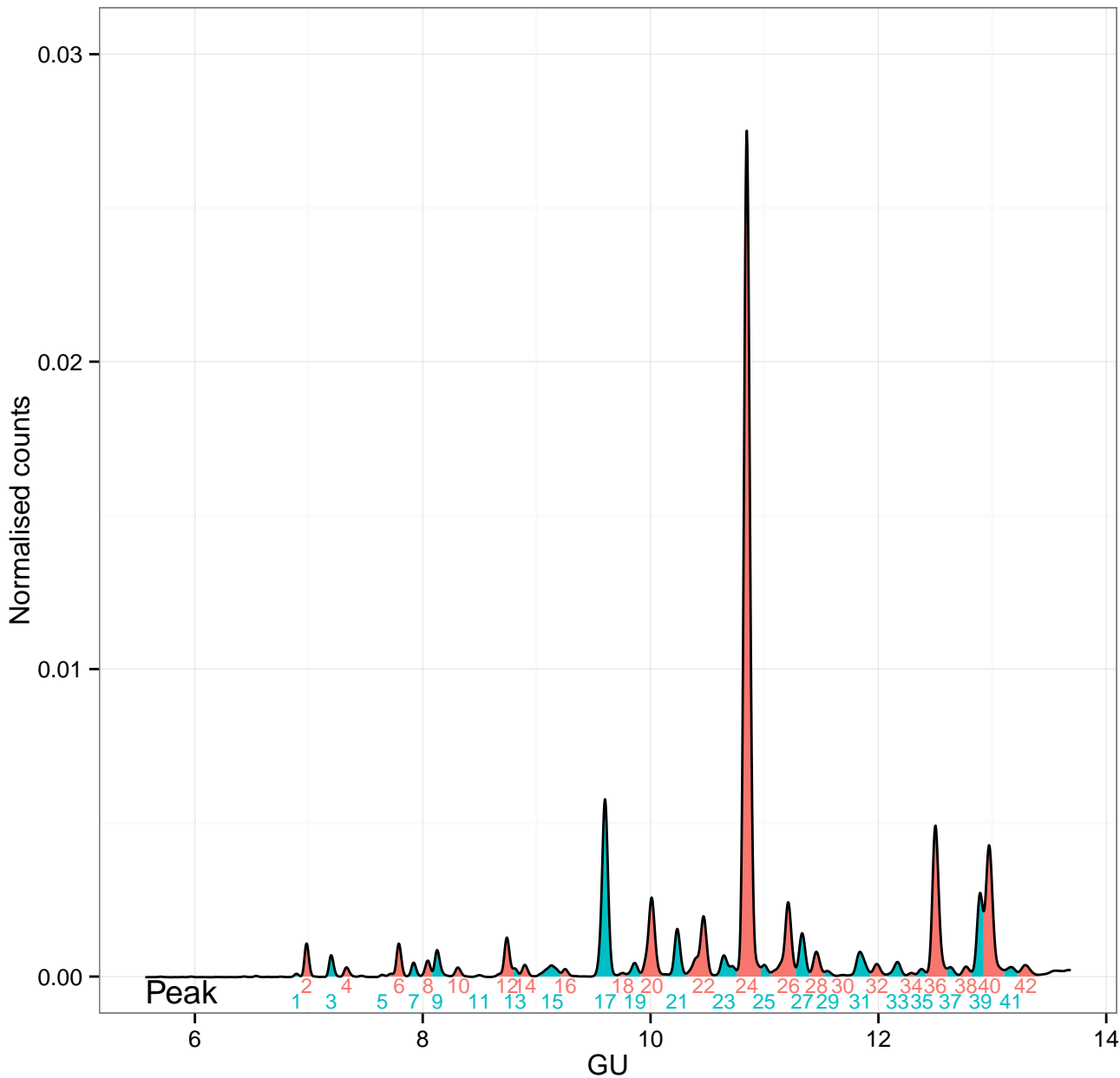

# H-3

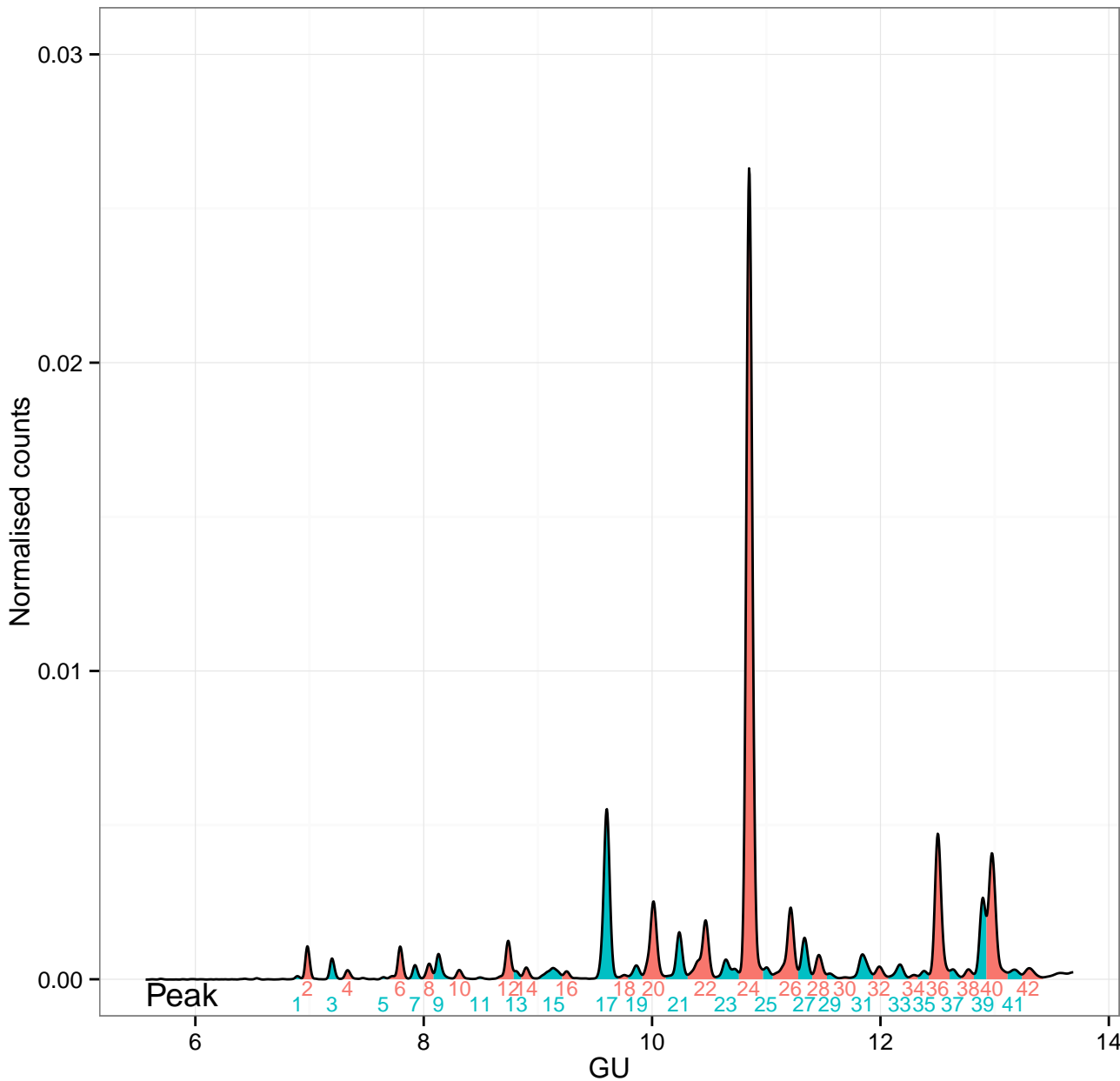

I-1

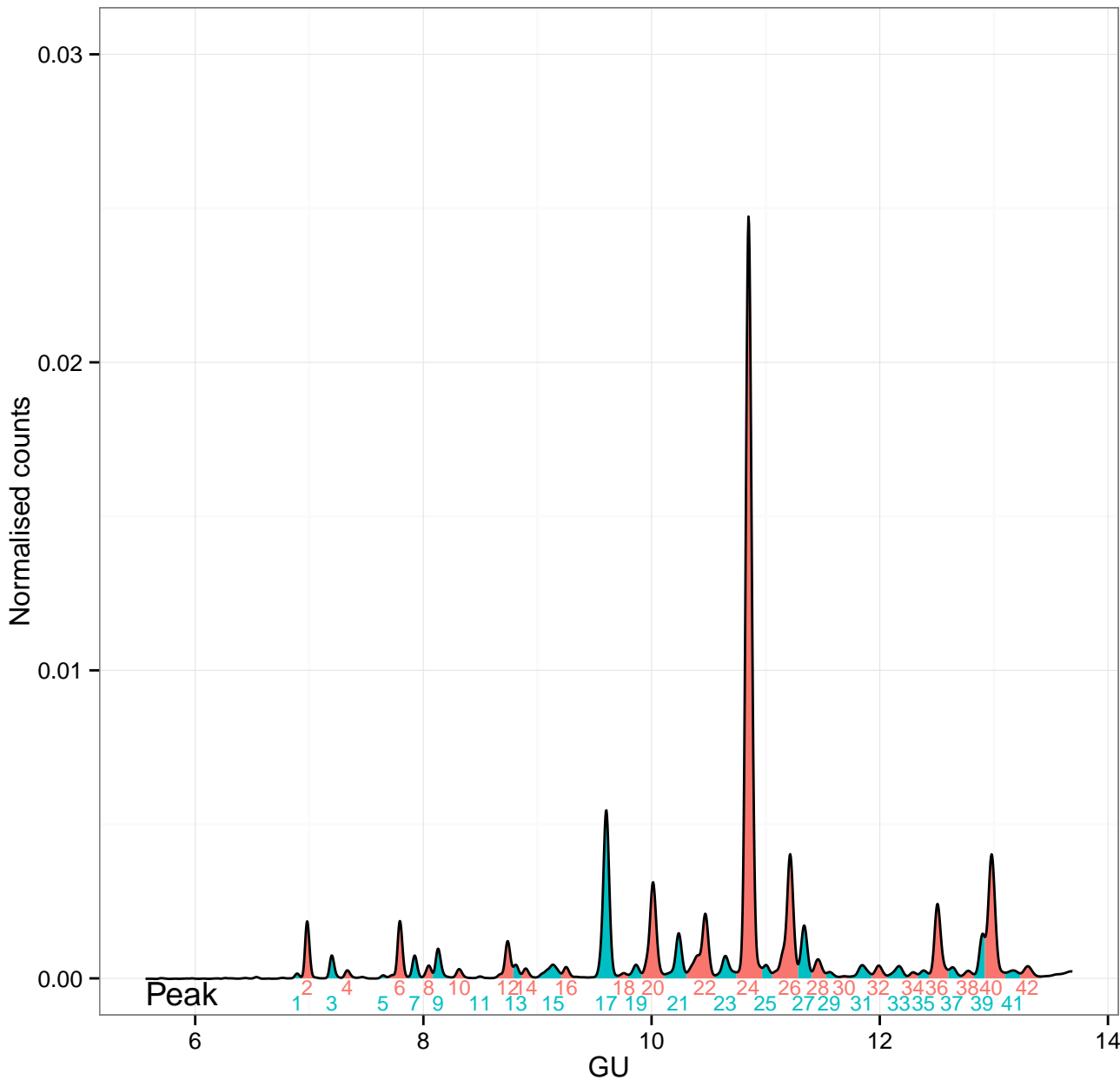

I-2

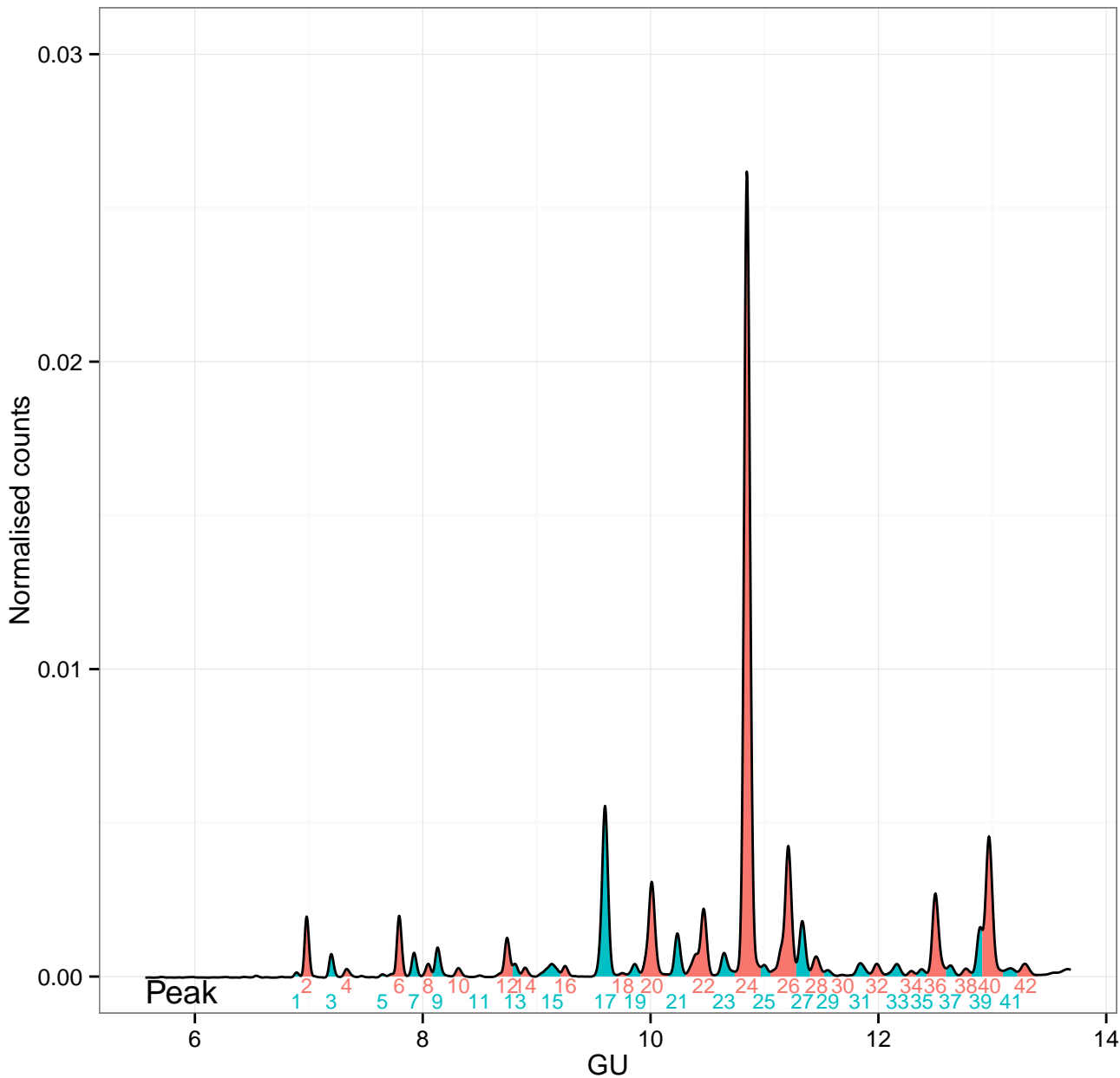

I-3

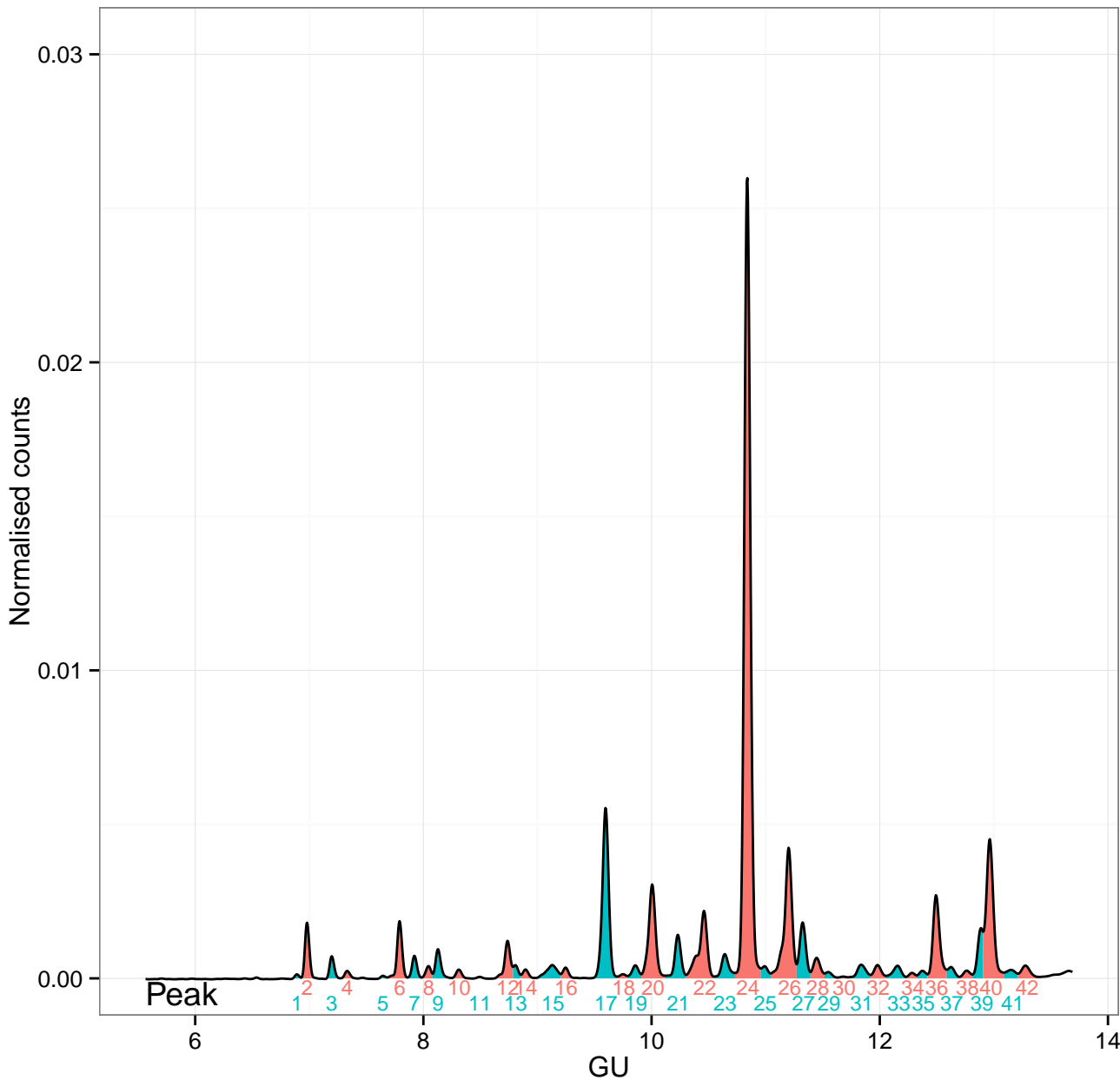

J-1

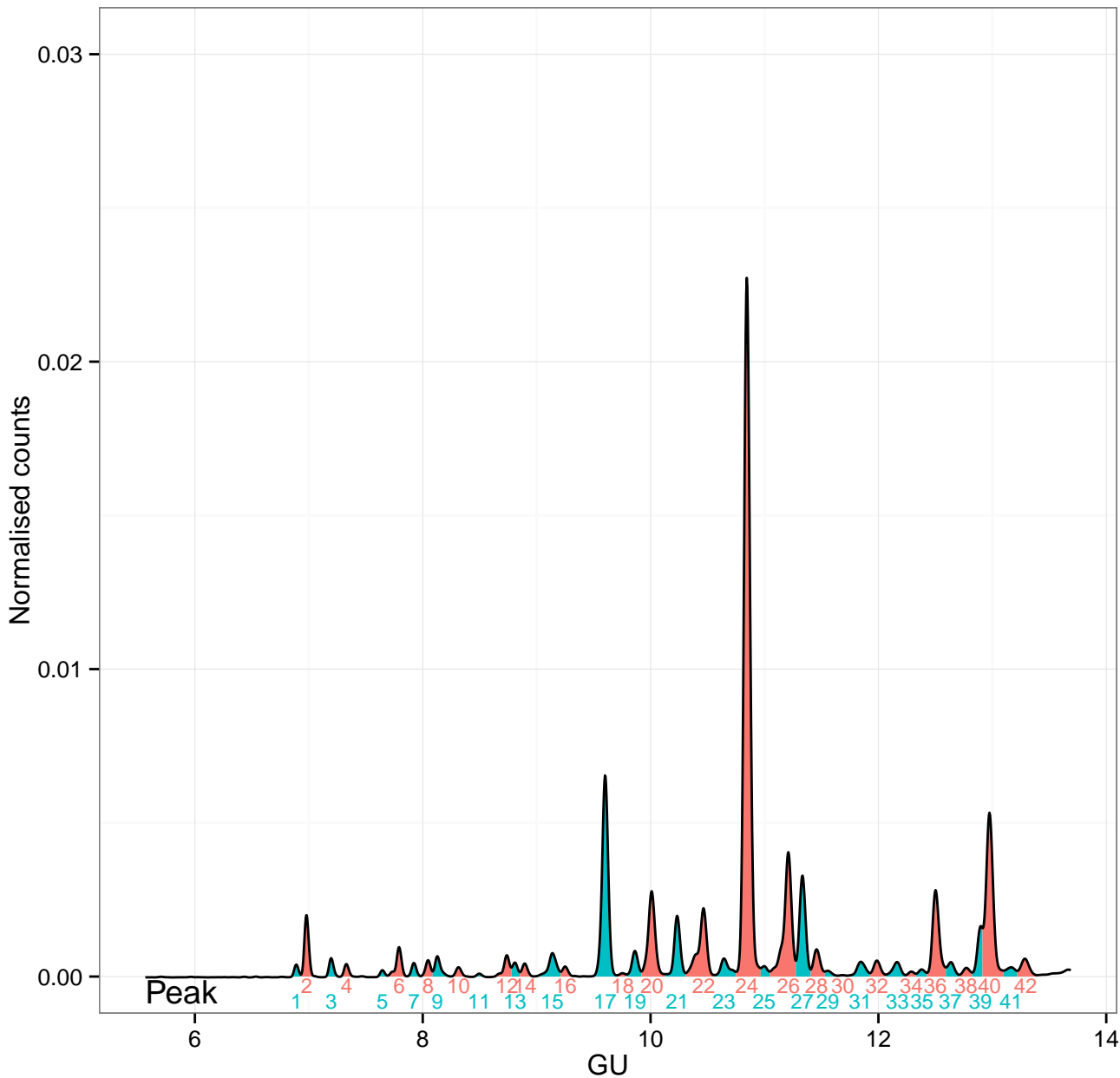

J-3

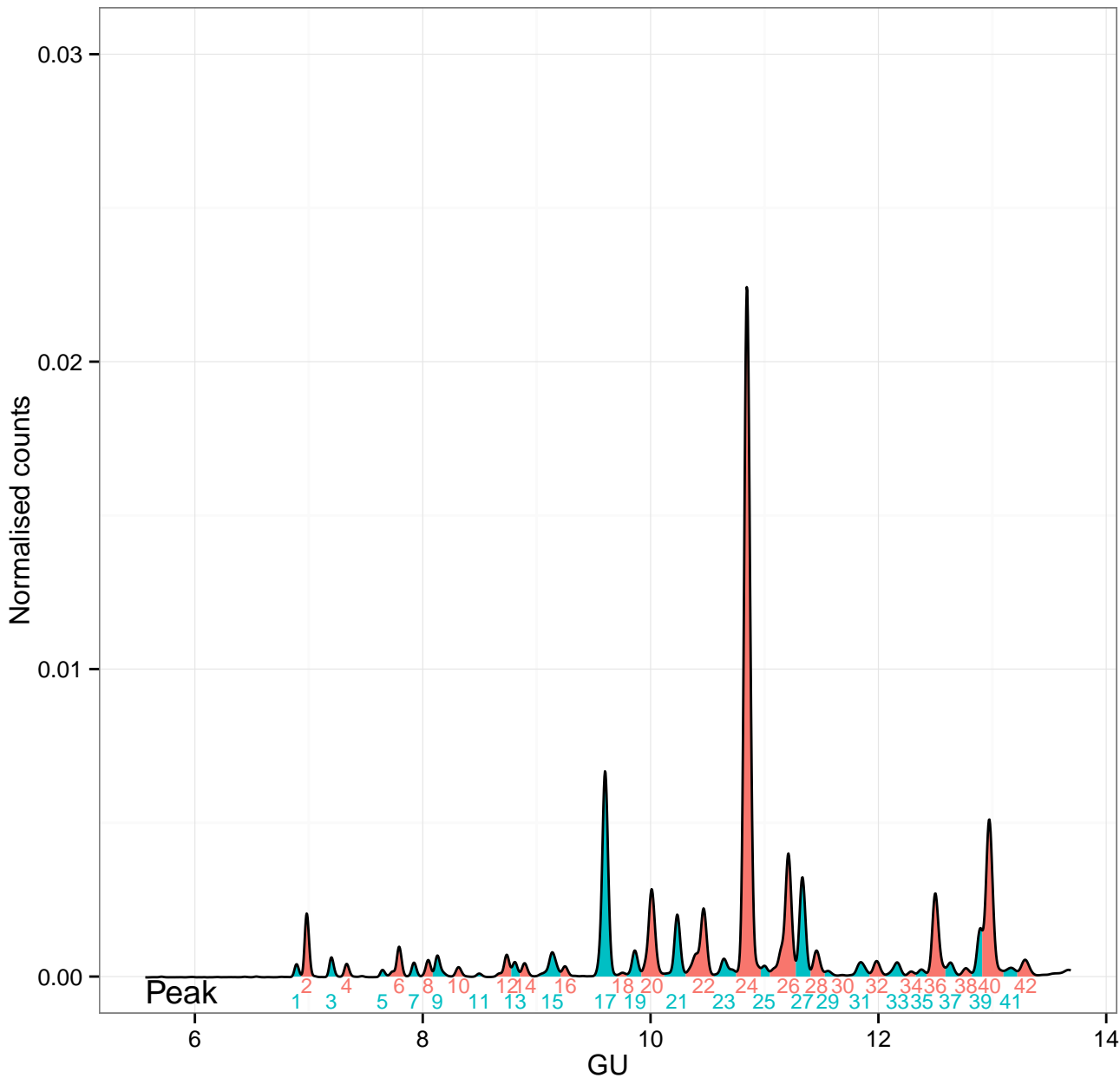

K-1

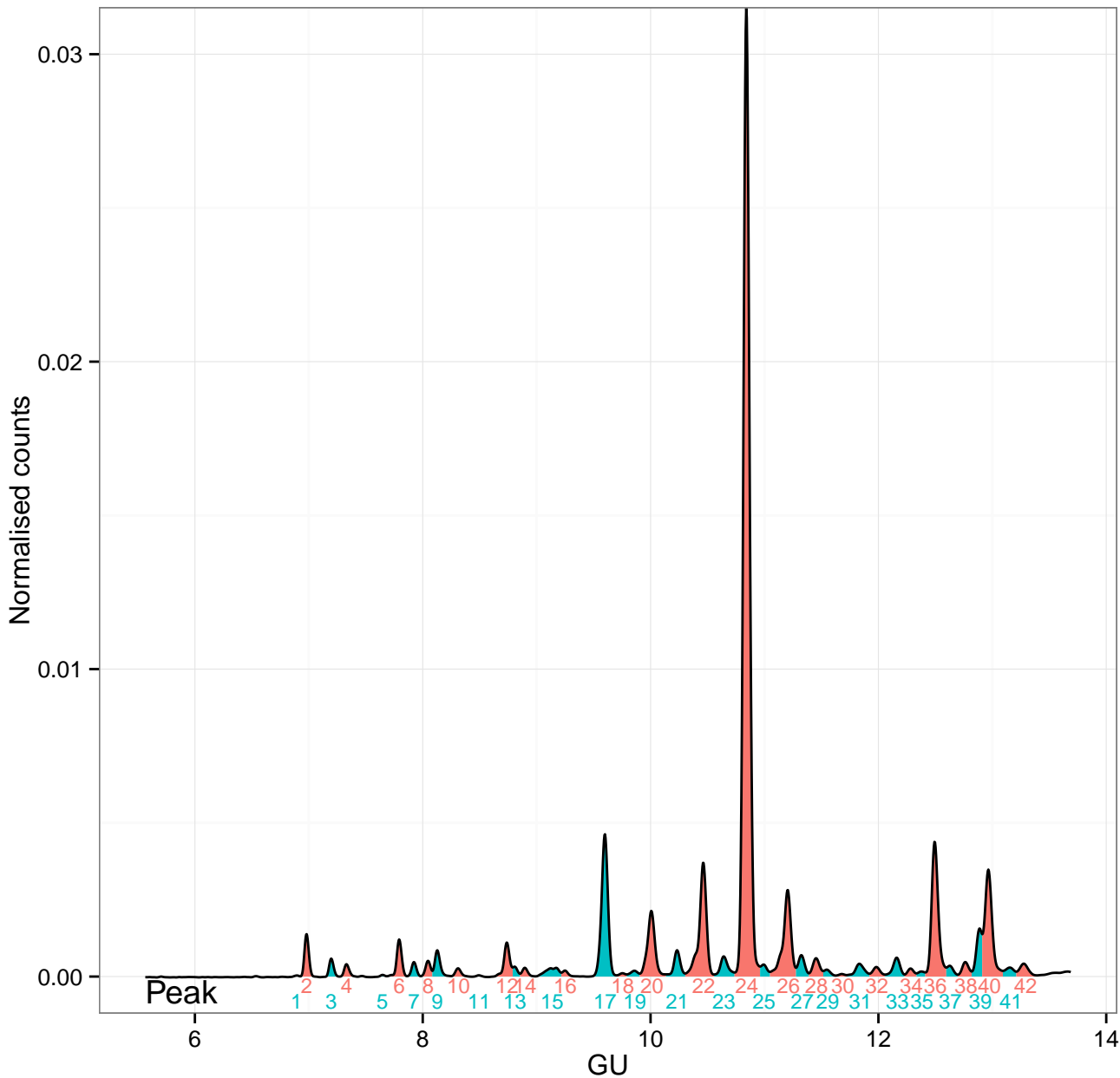

K-2

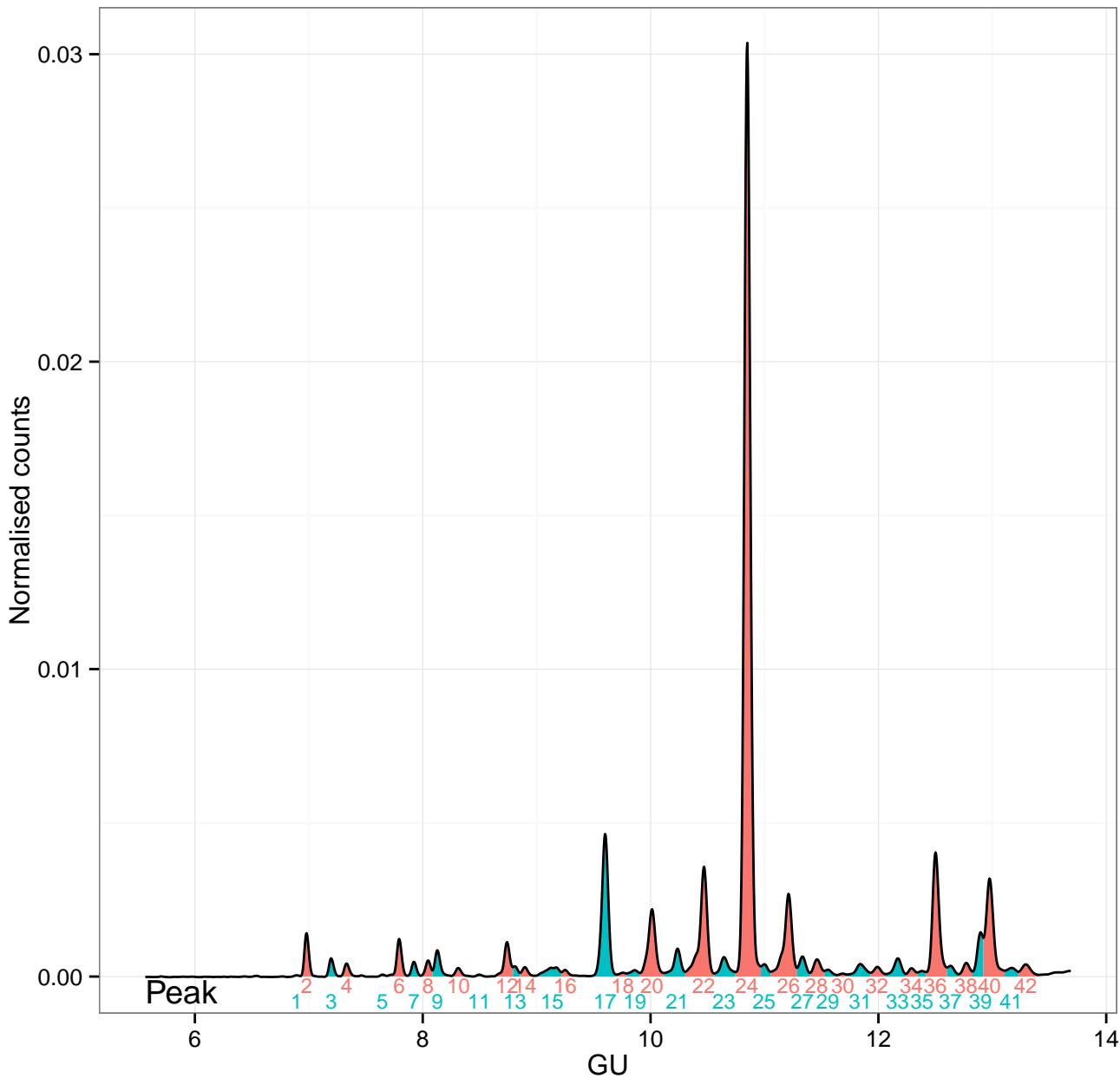

K-3

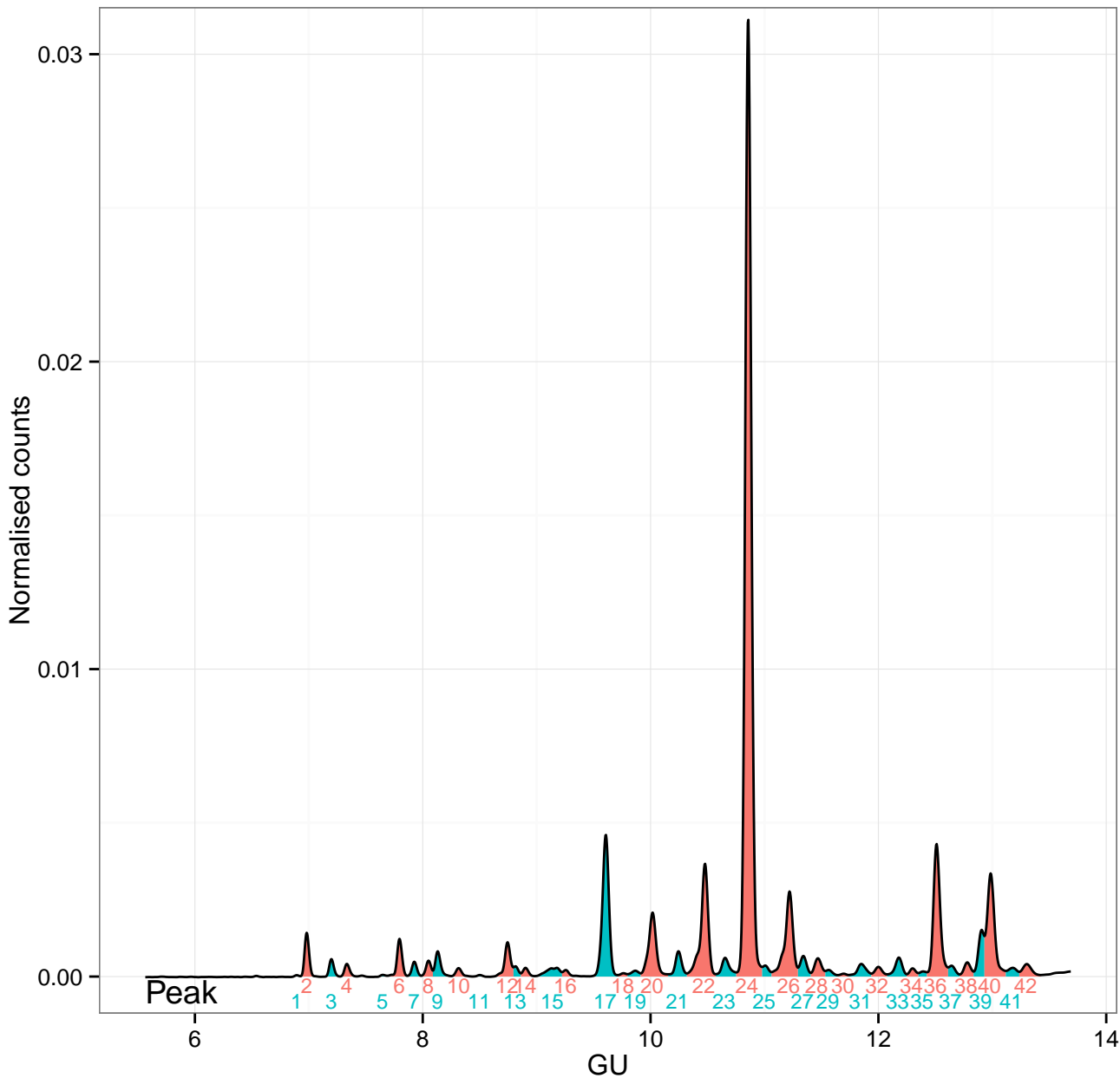

L-1

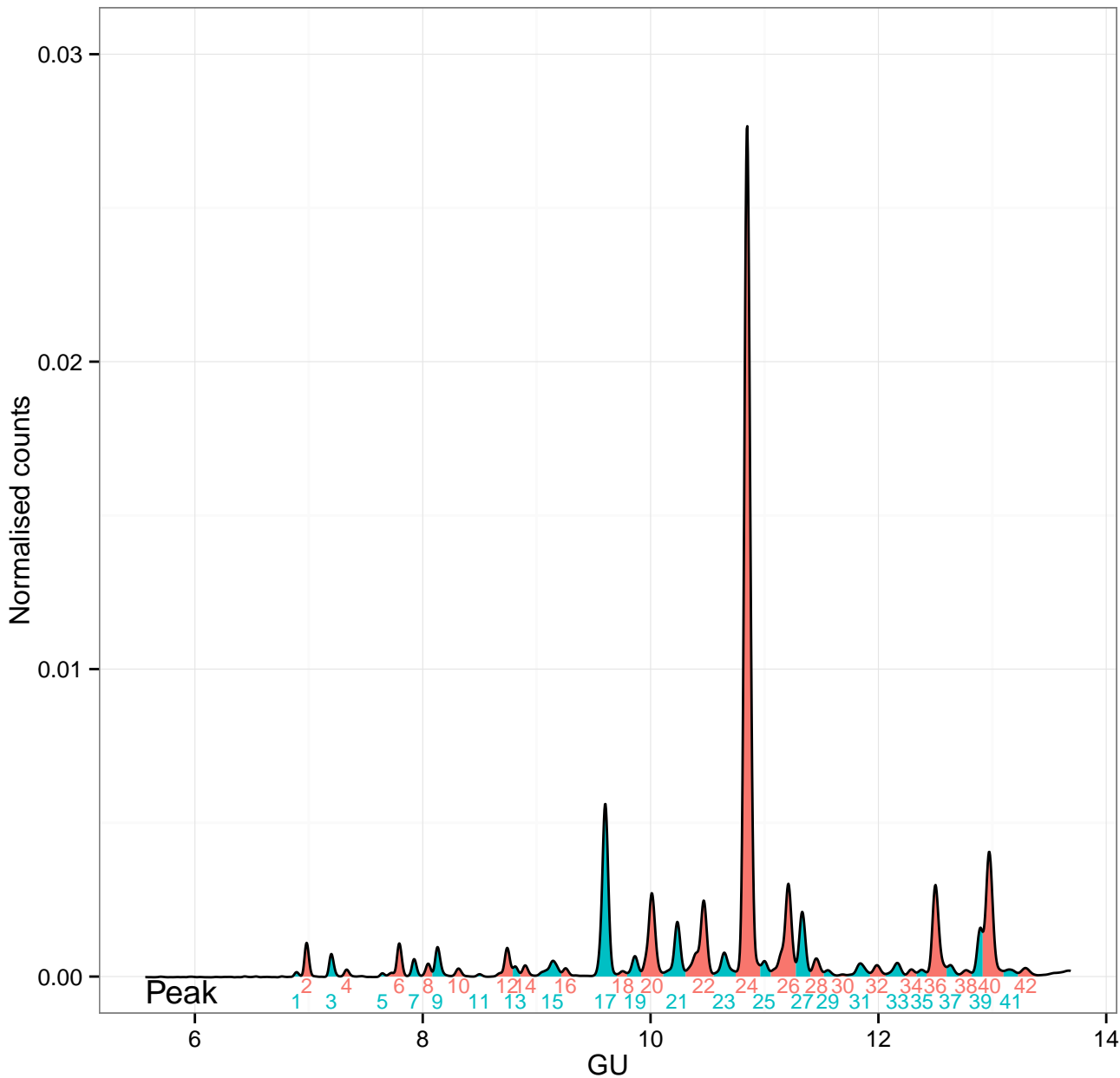

L-3

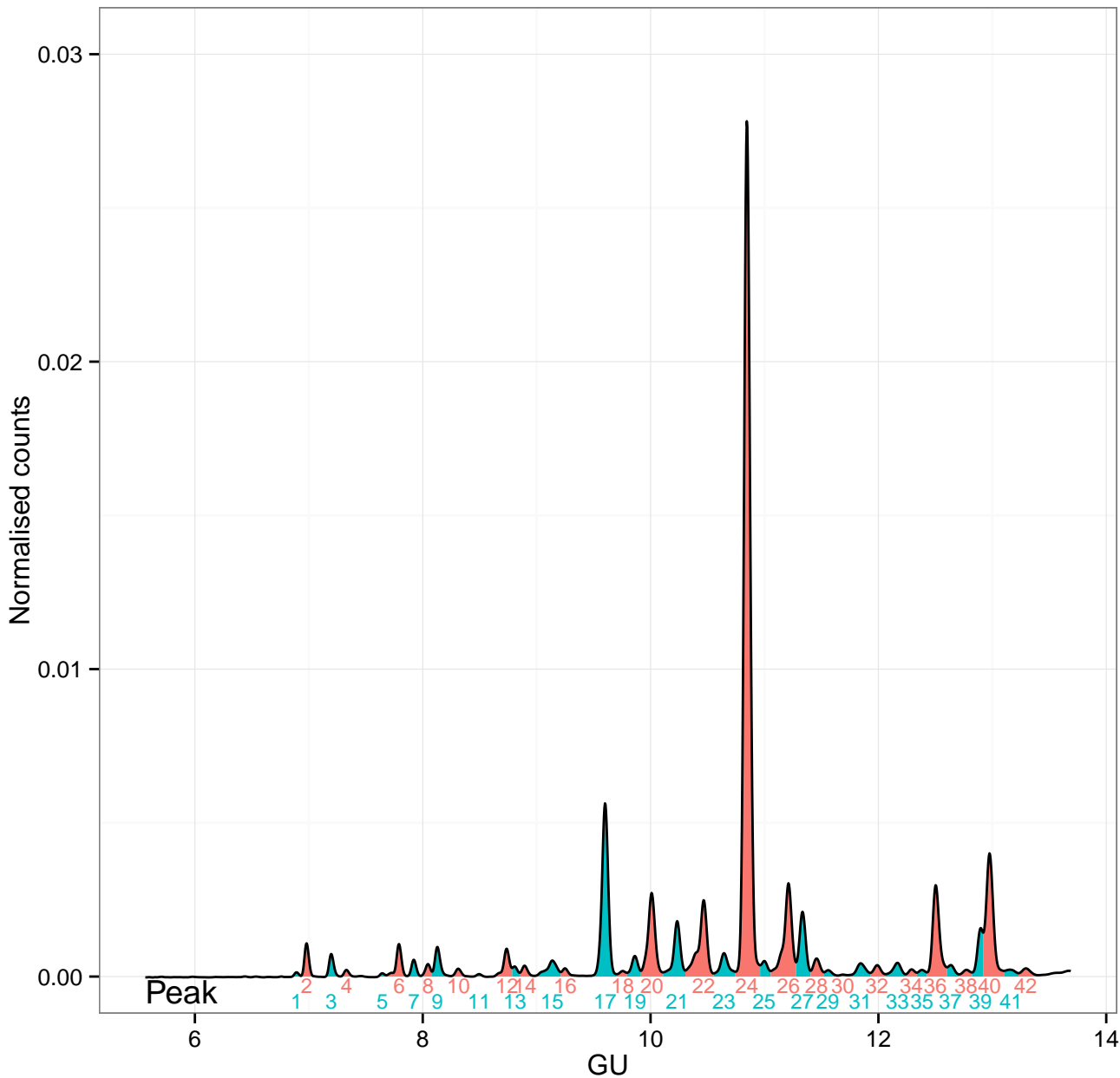

M-1

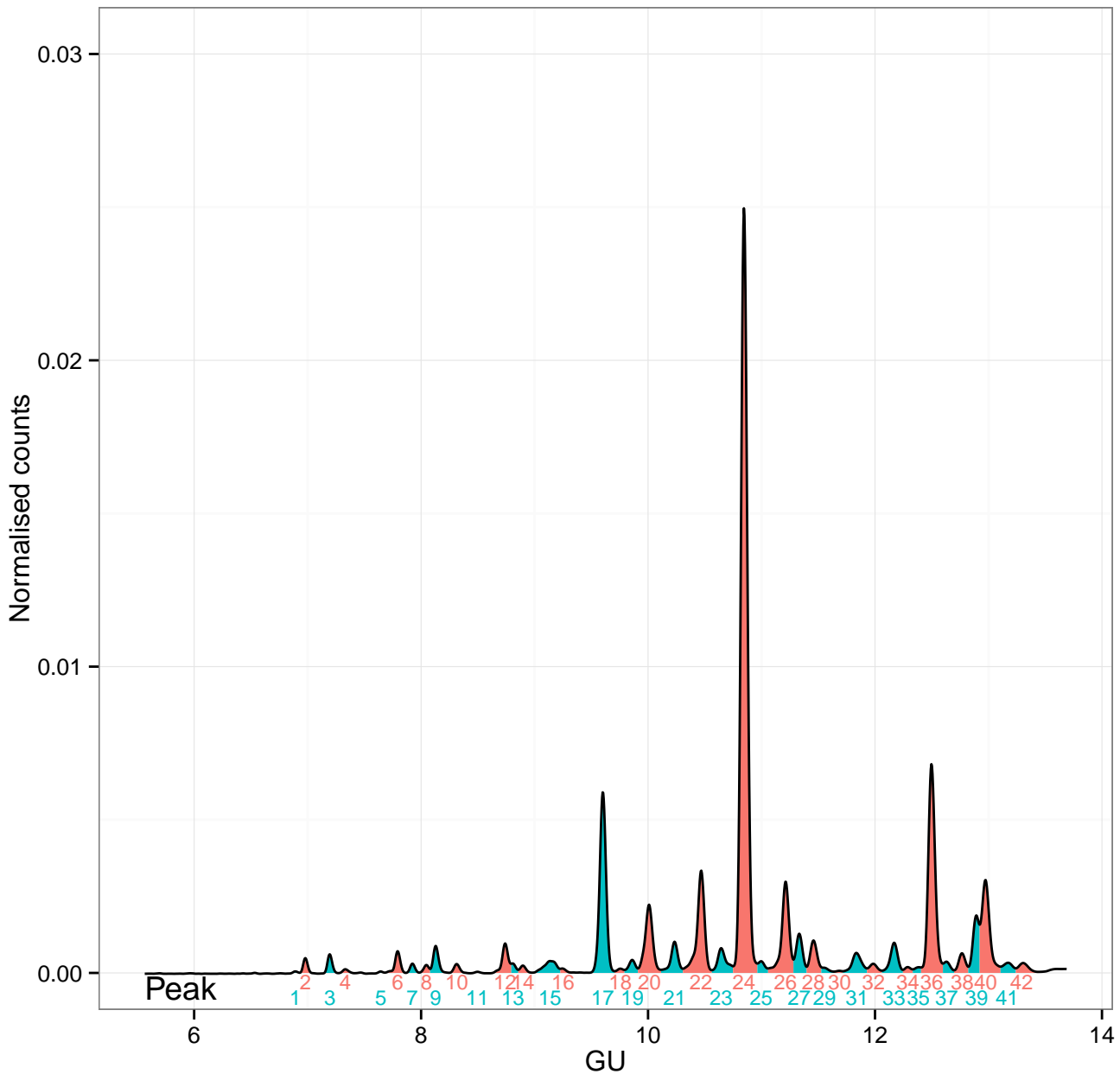

M-2

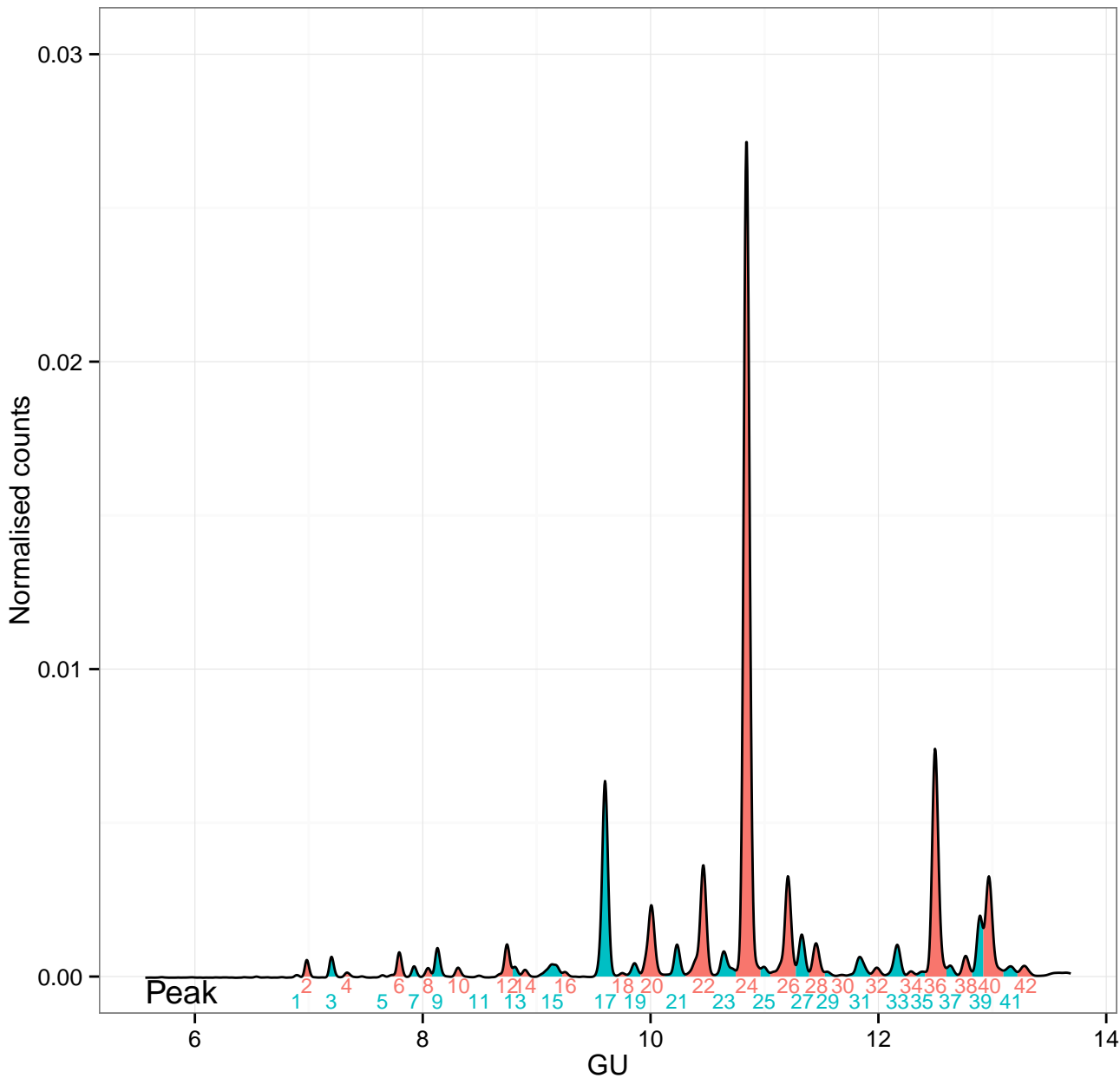

# M-3

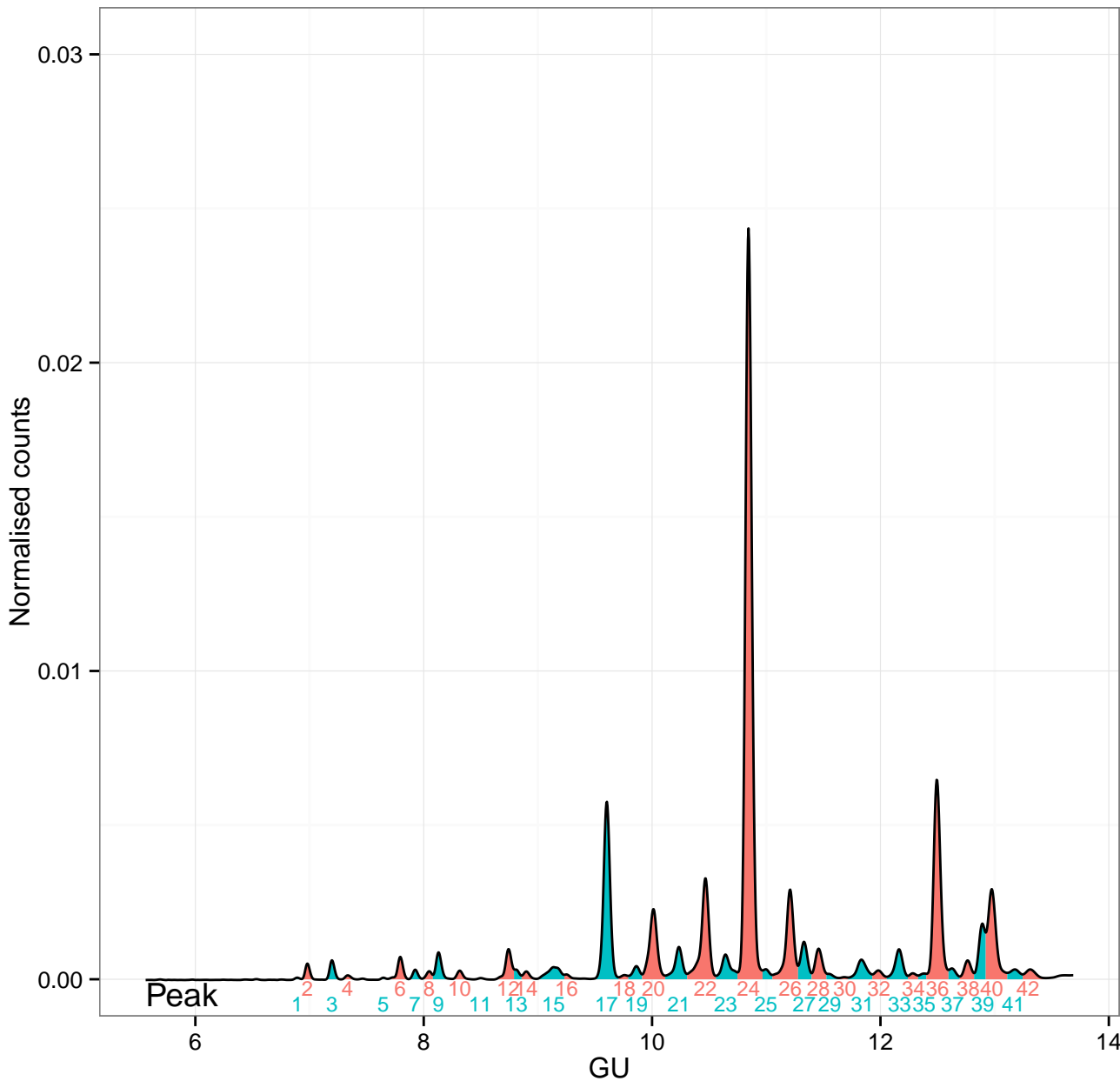

N-1

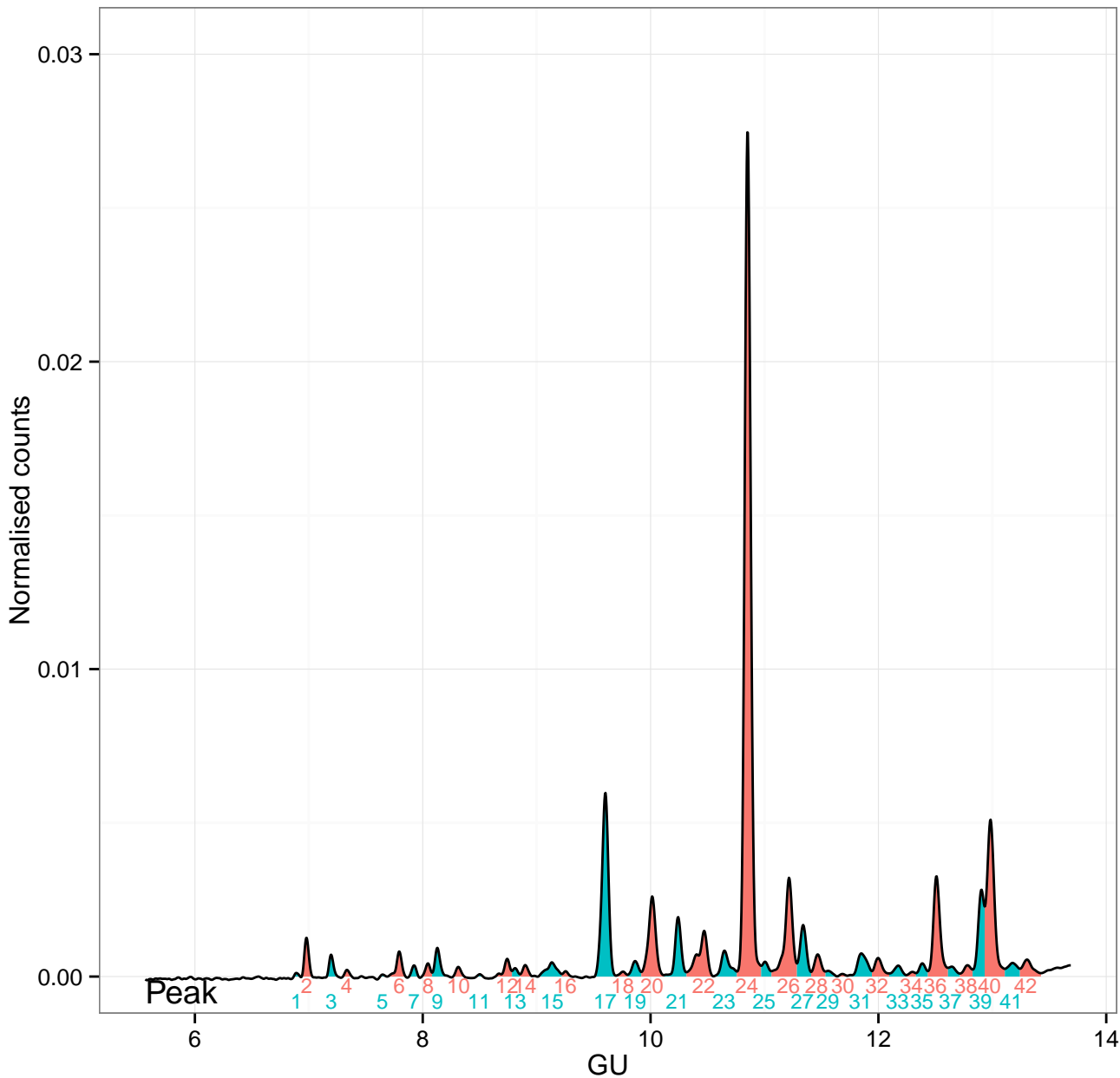

N-3

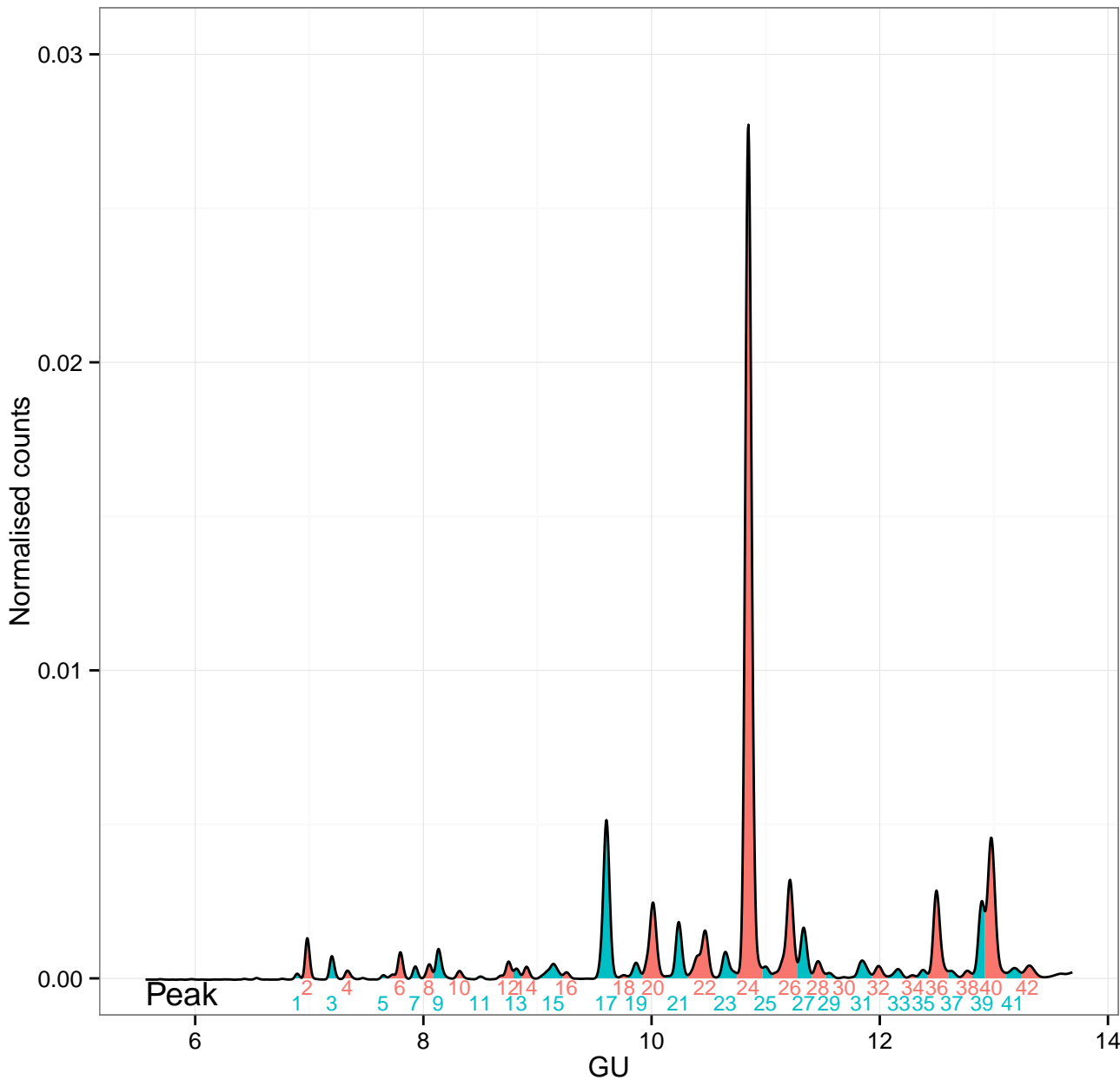

O-1

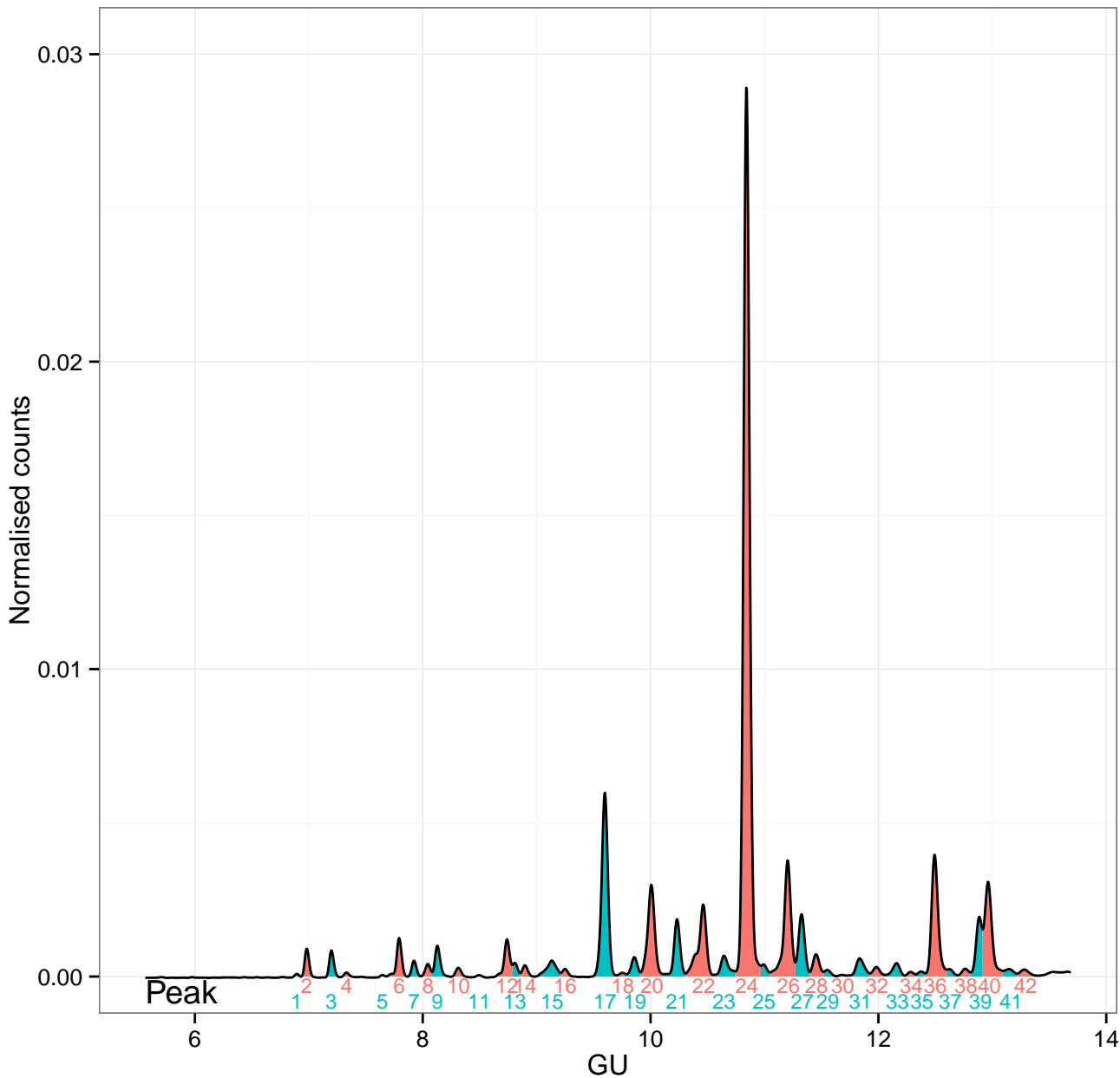

O-2

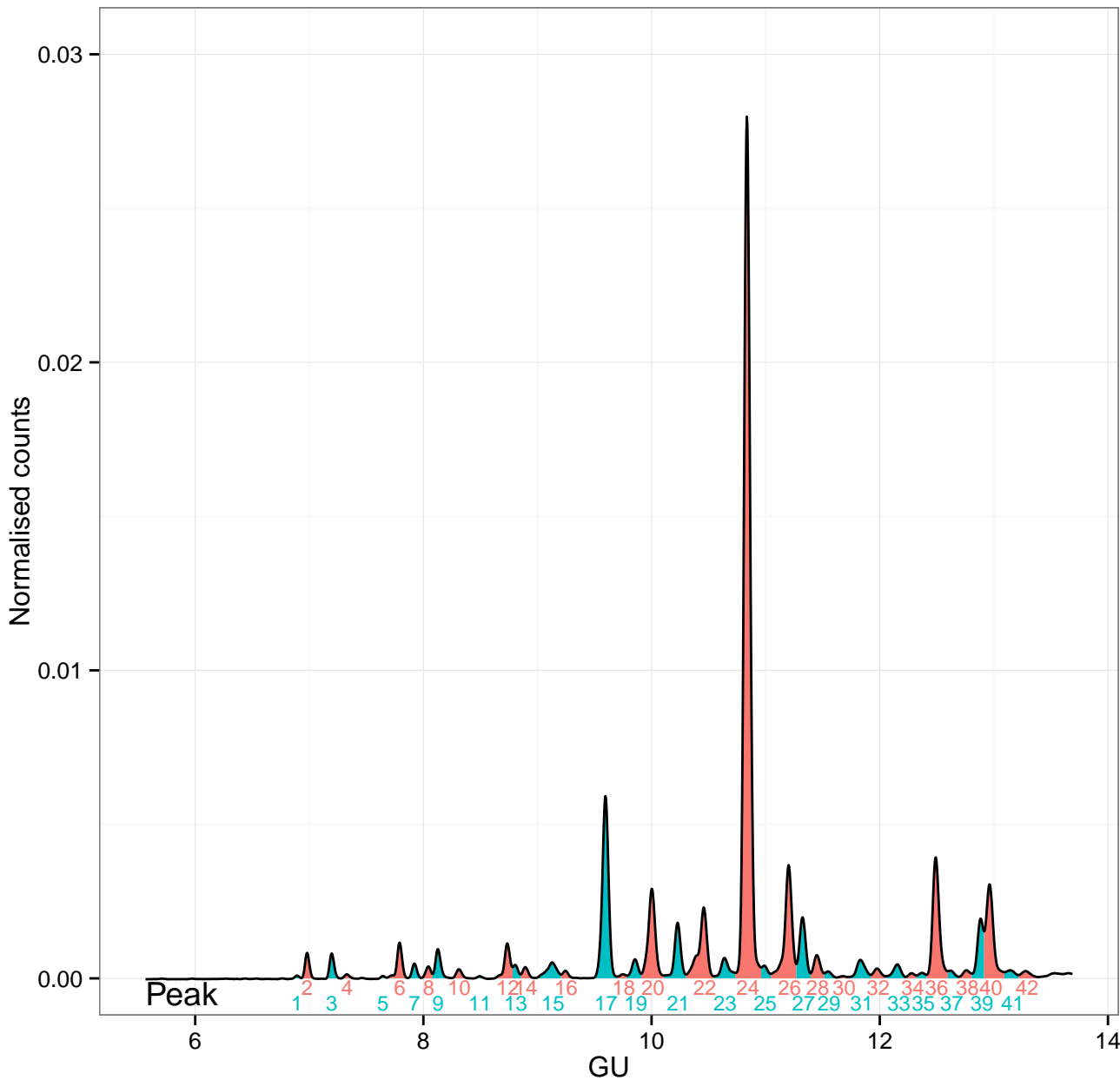

O-3

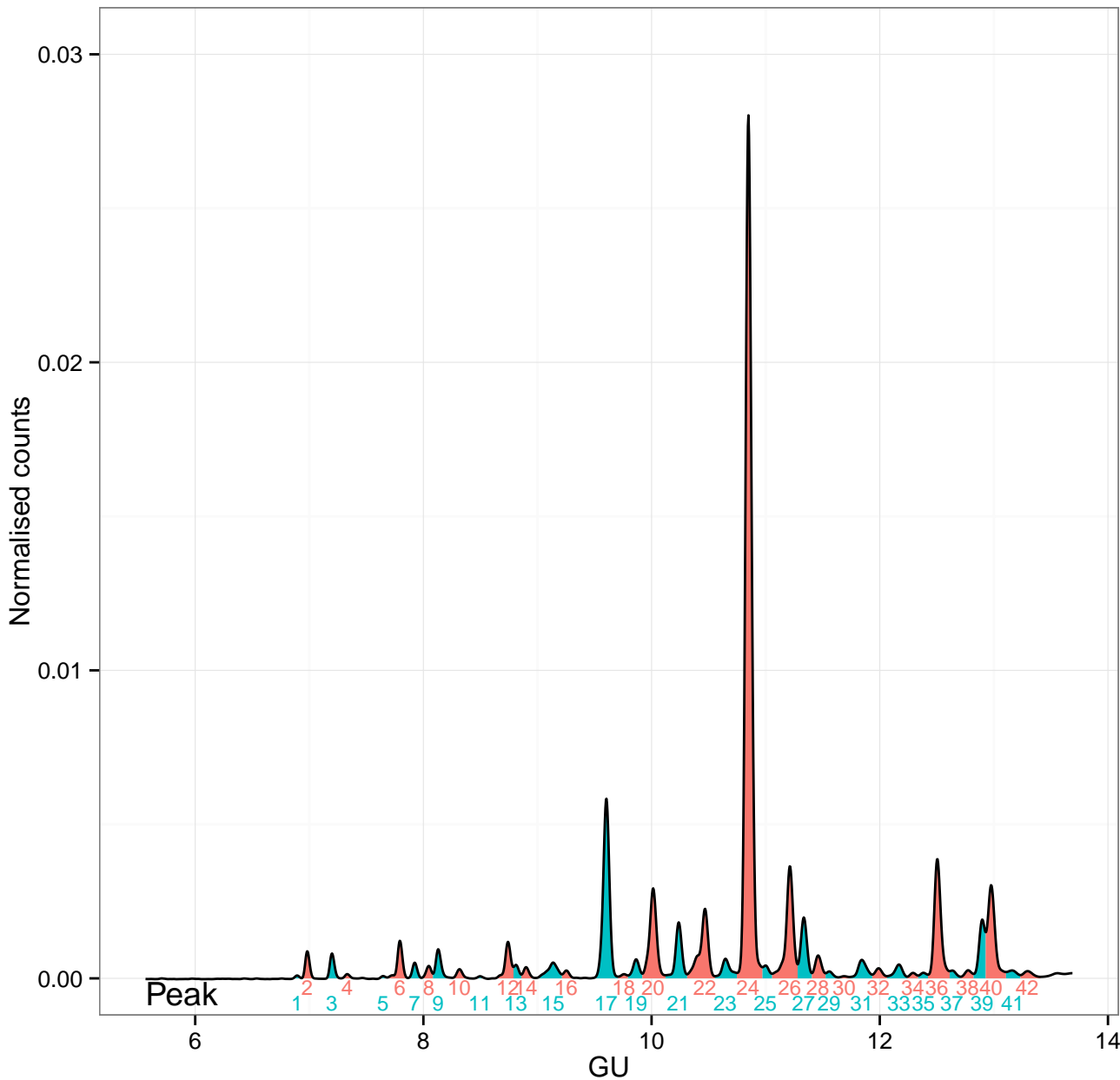

P-1

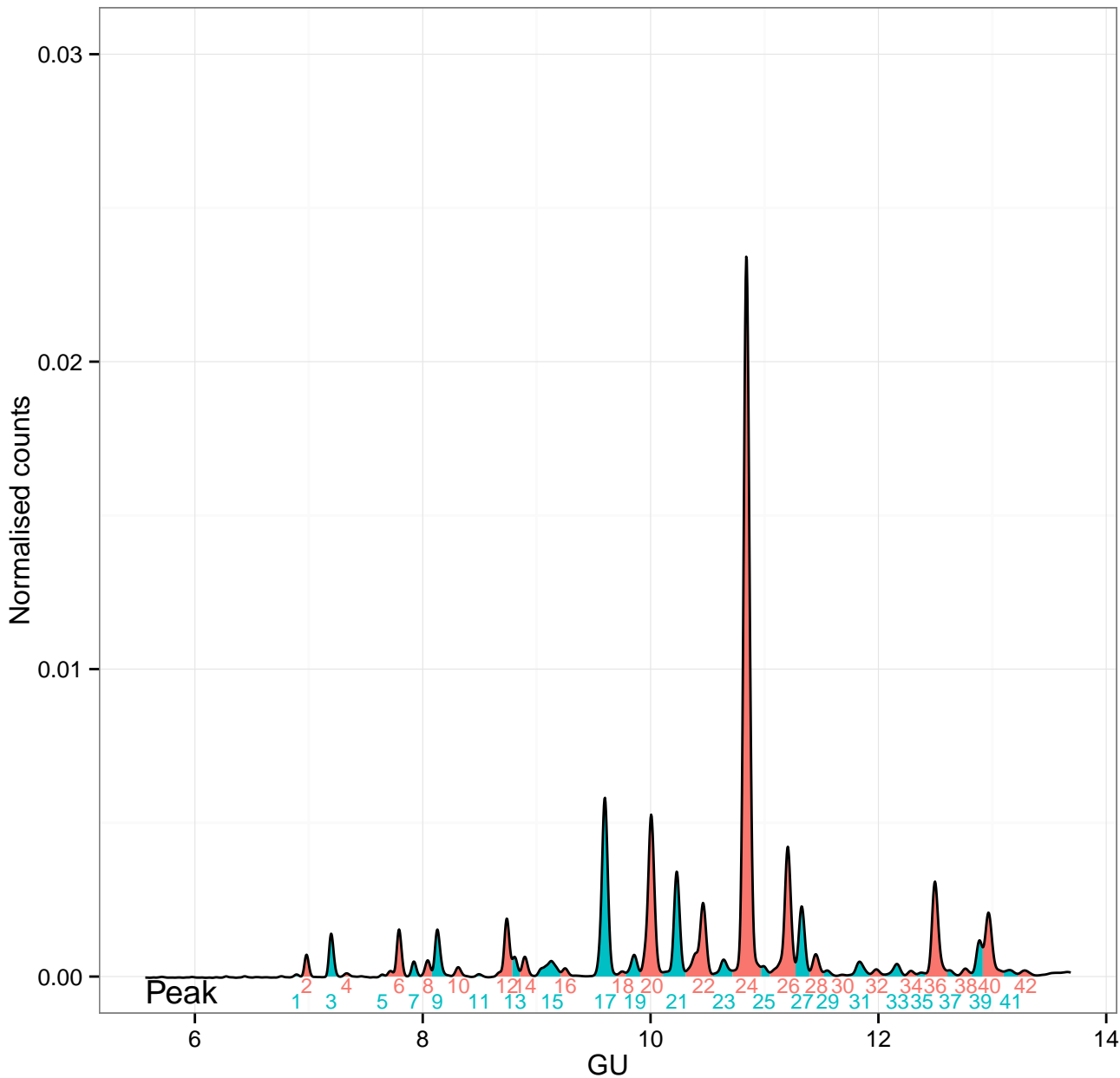

P-3

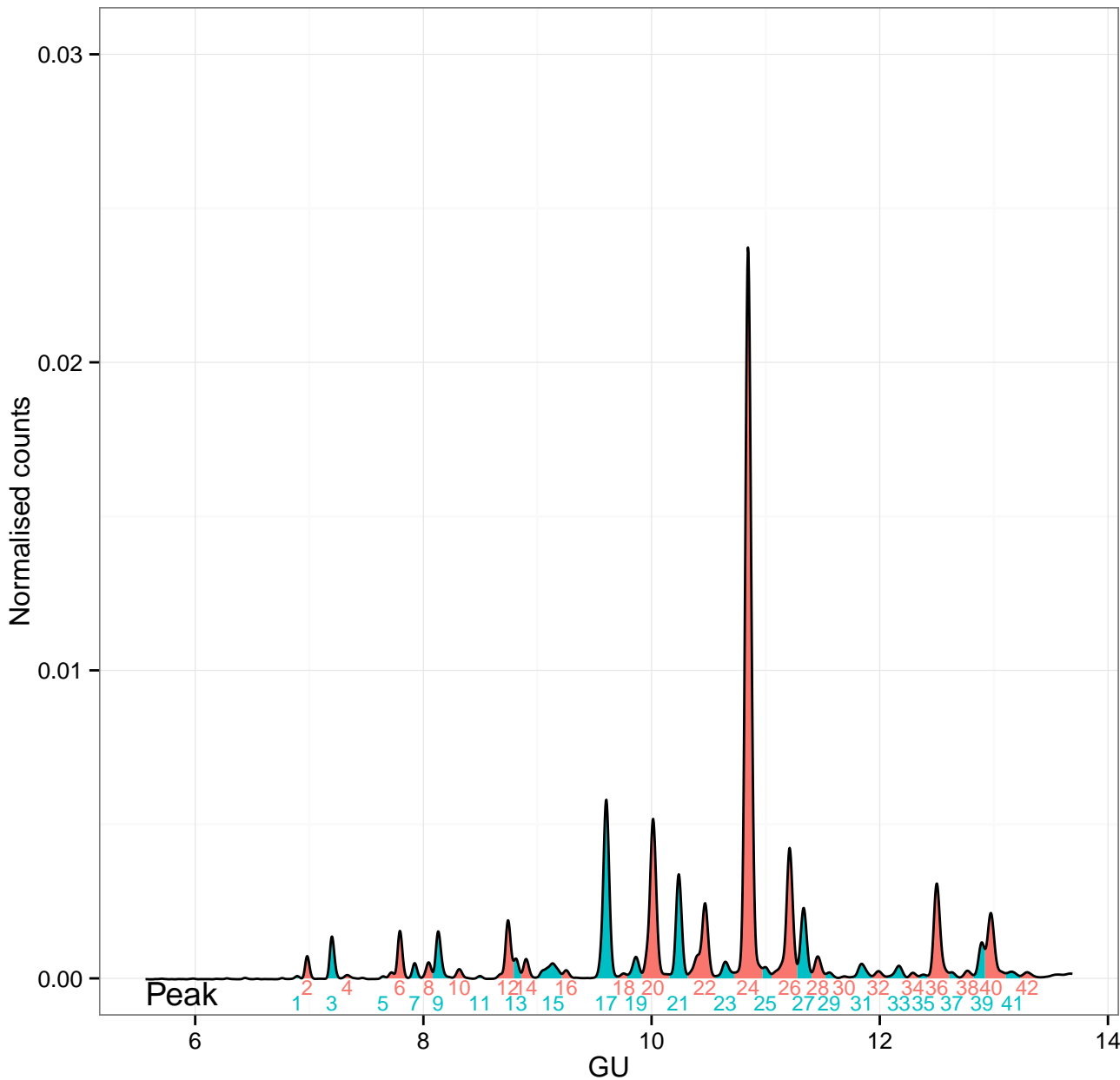

Q-1

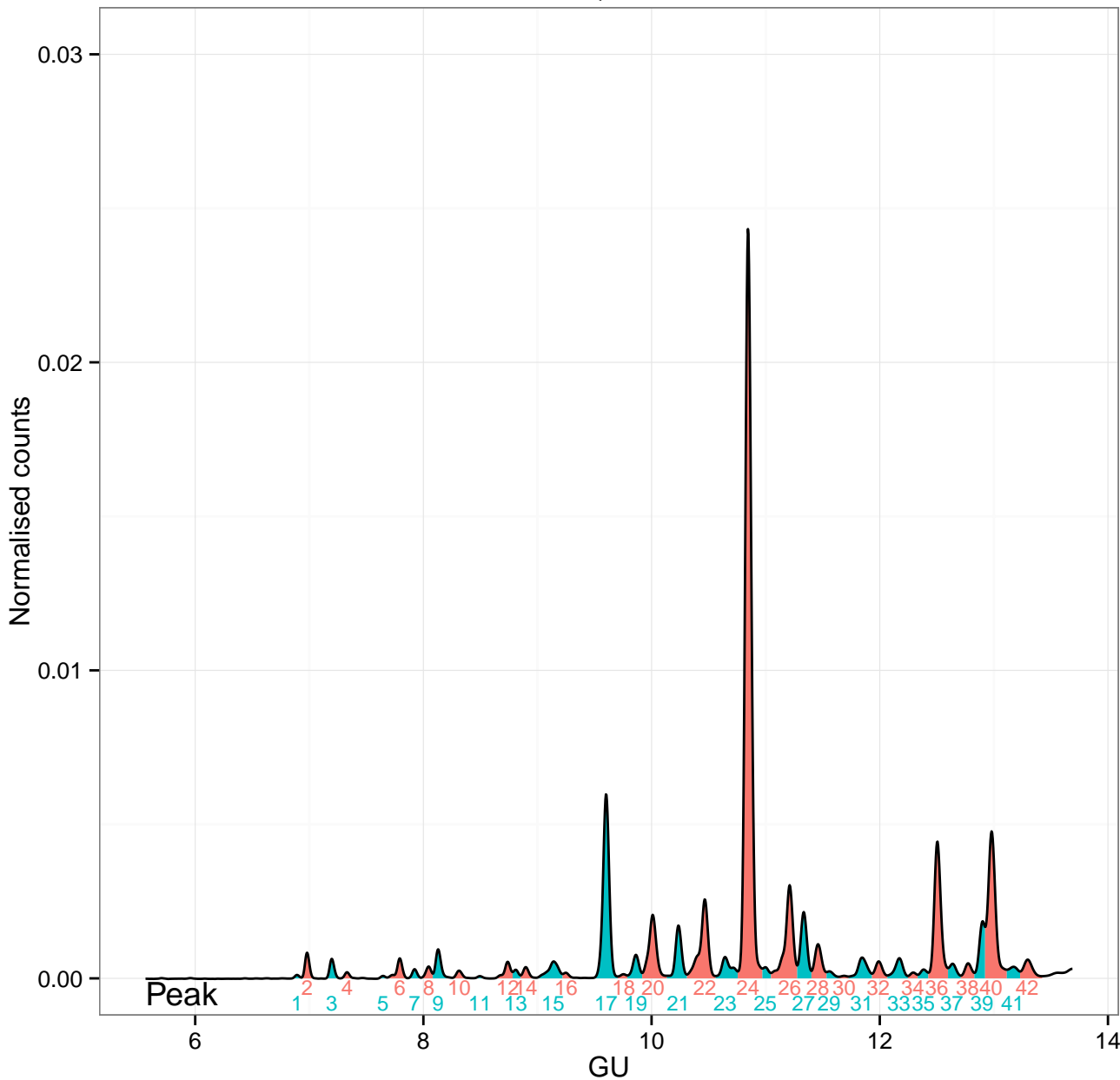

Q-2

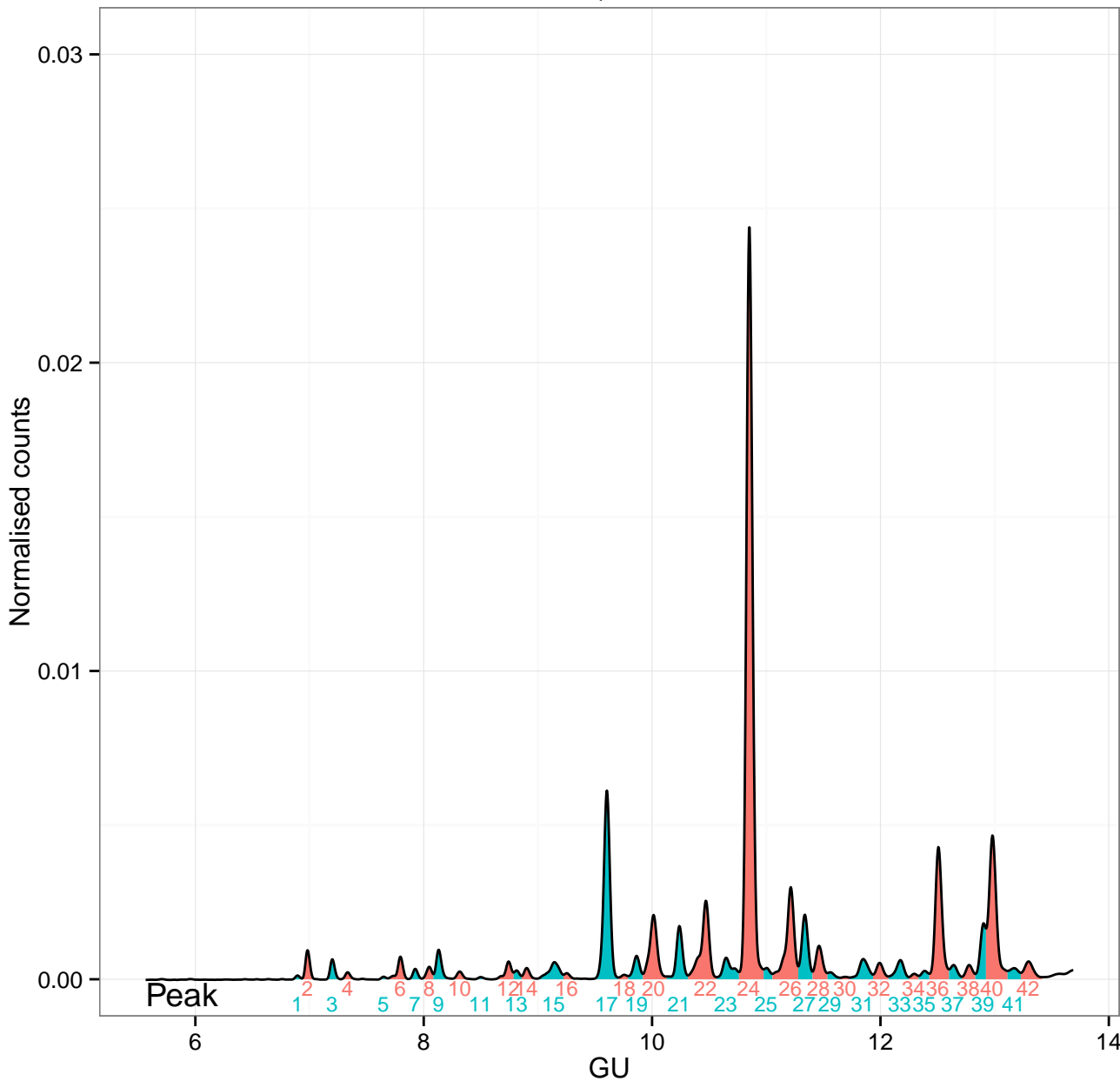

Q-3

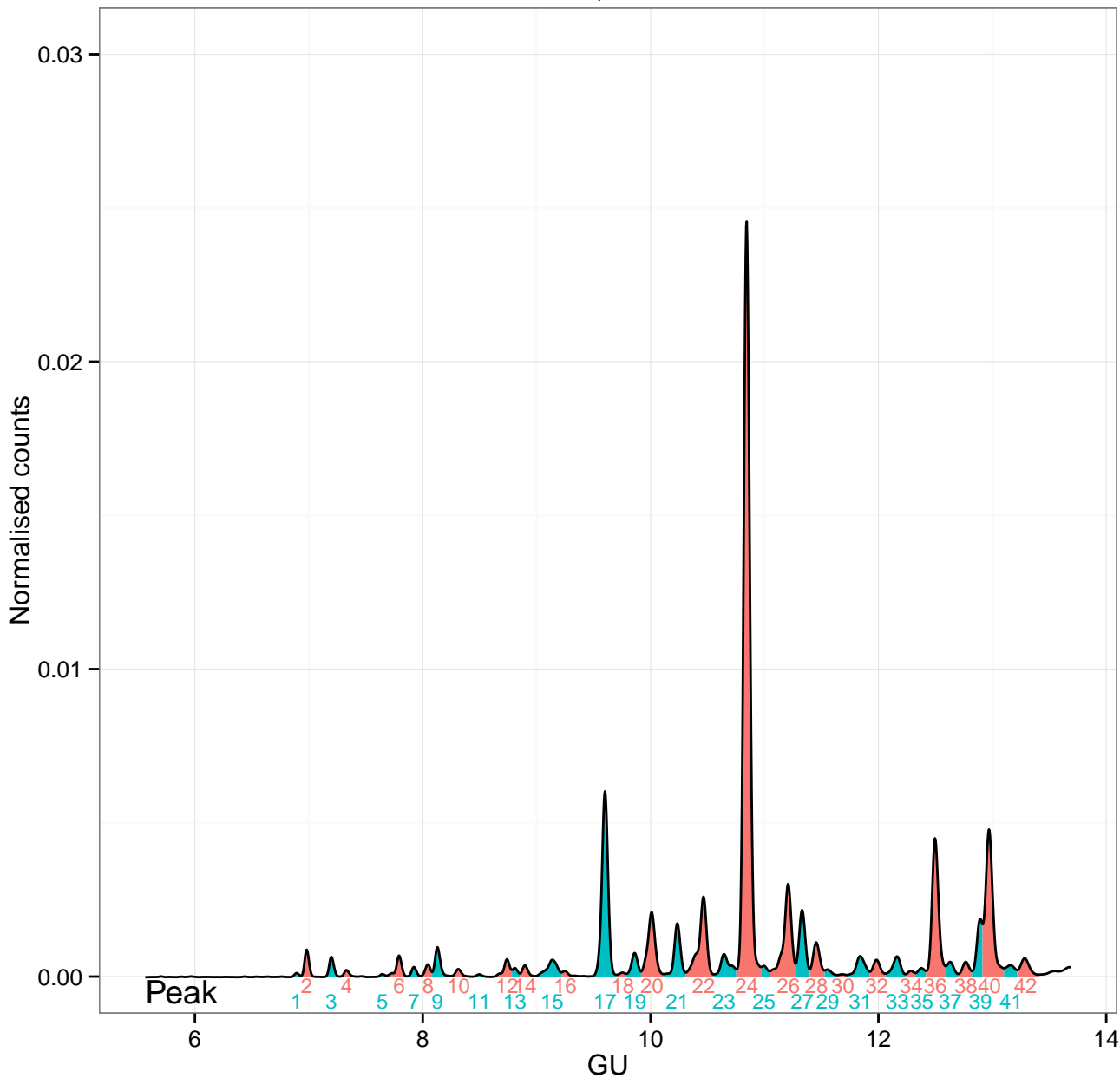

R-1

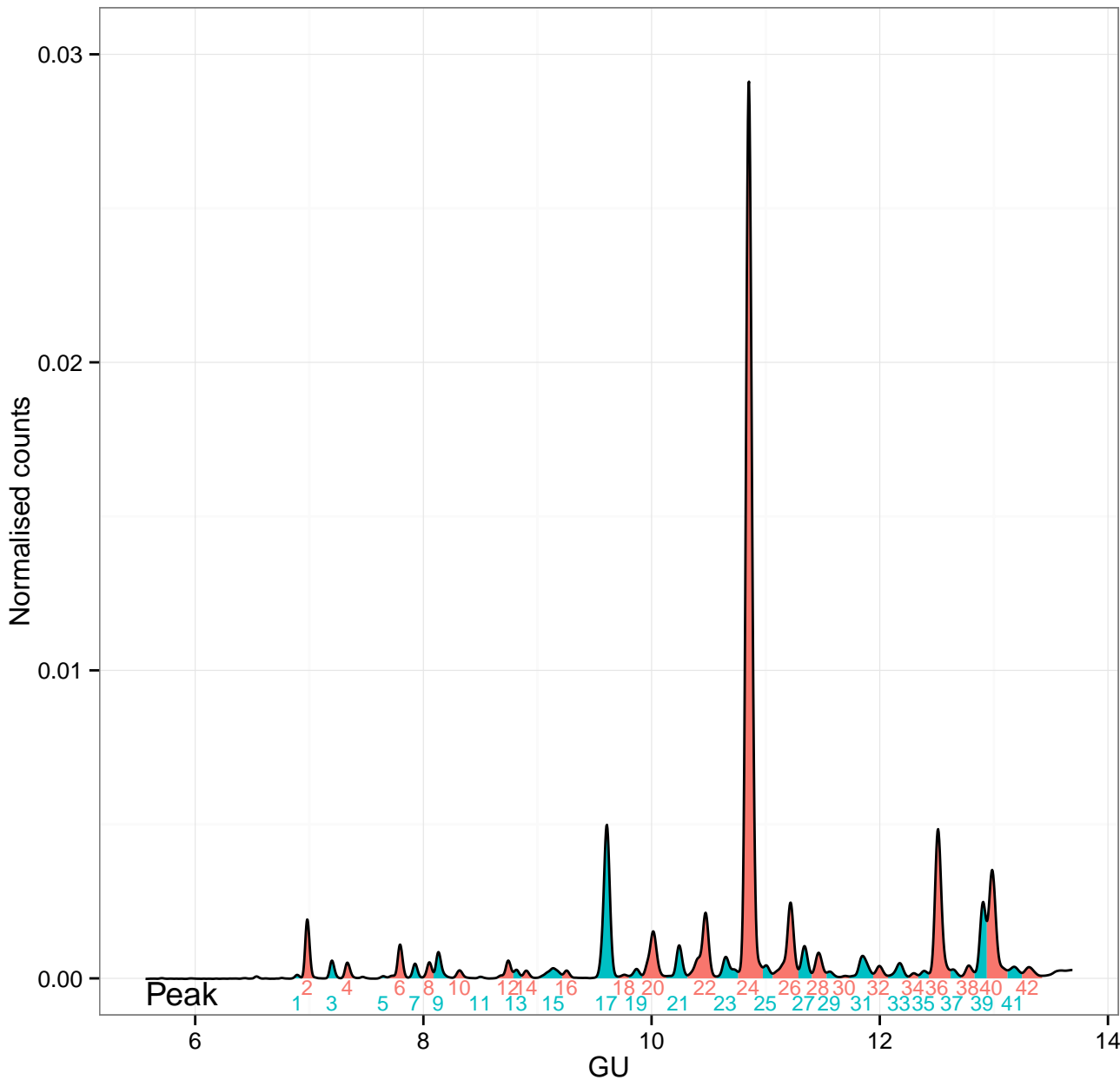

R-2

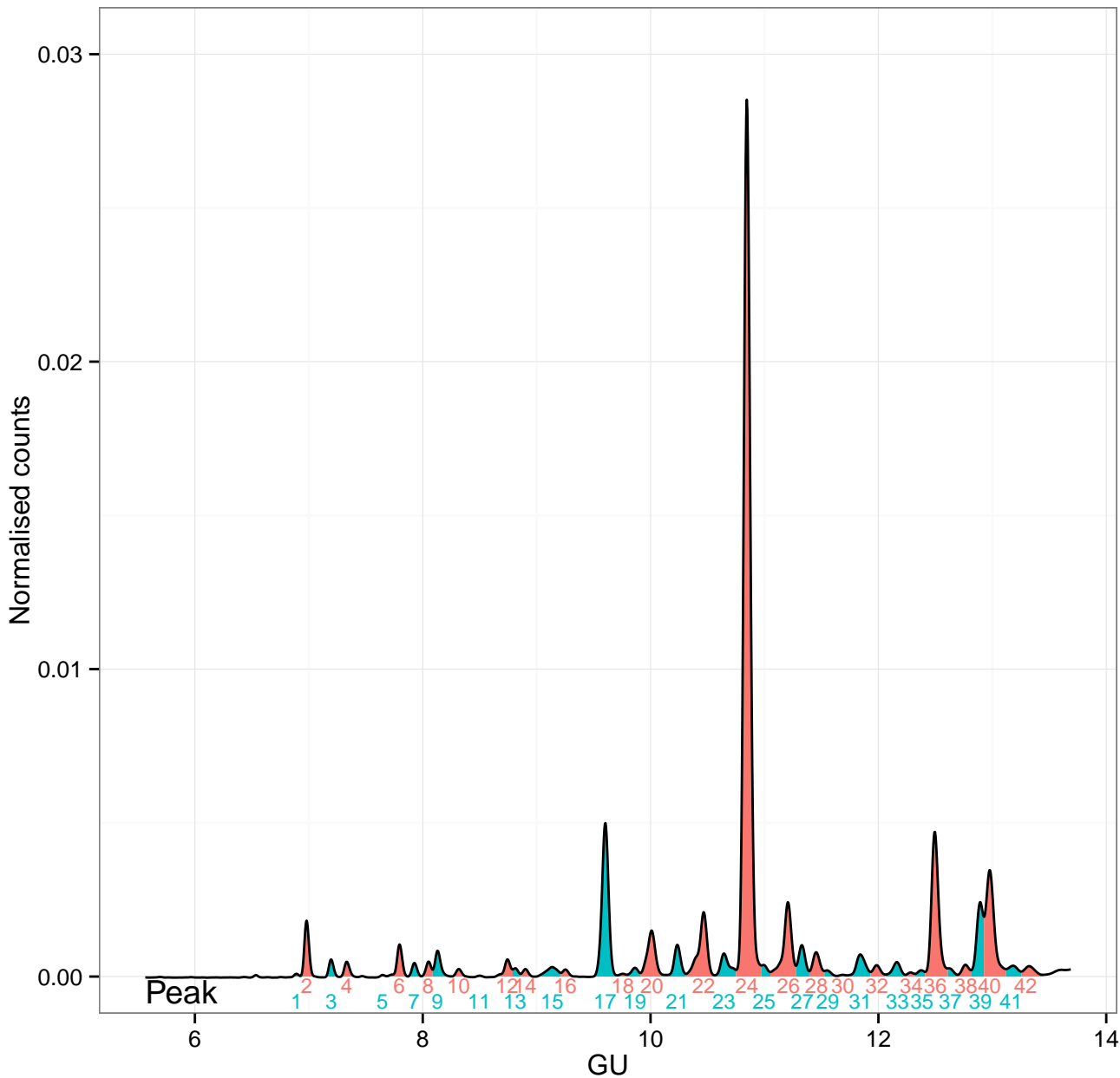

R-3

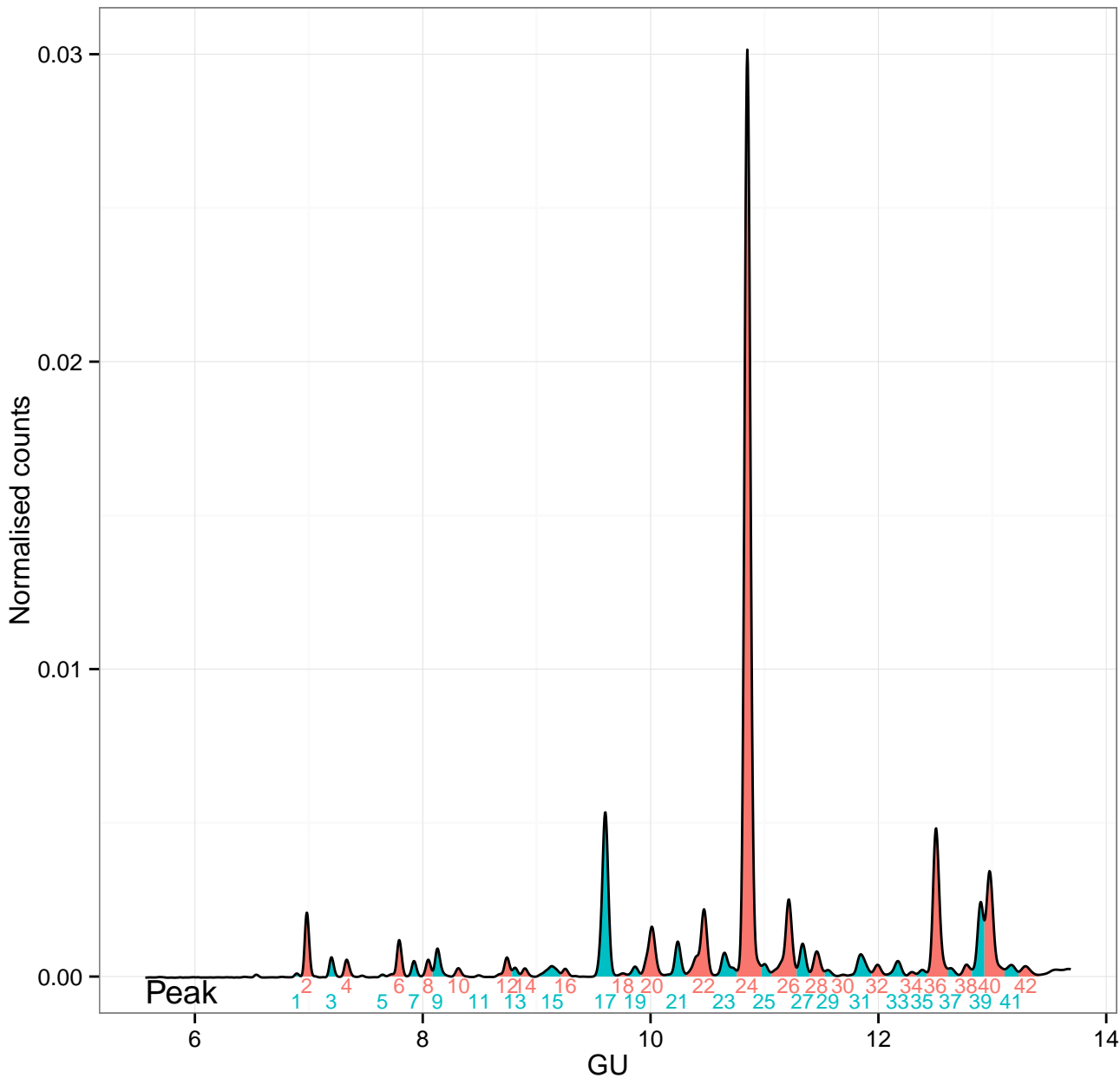

S-1

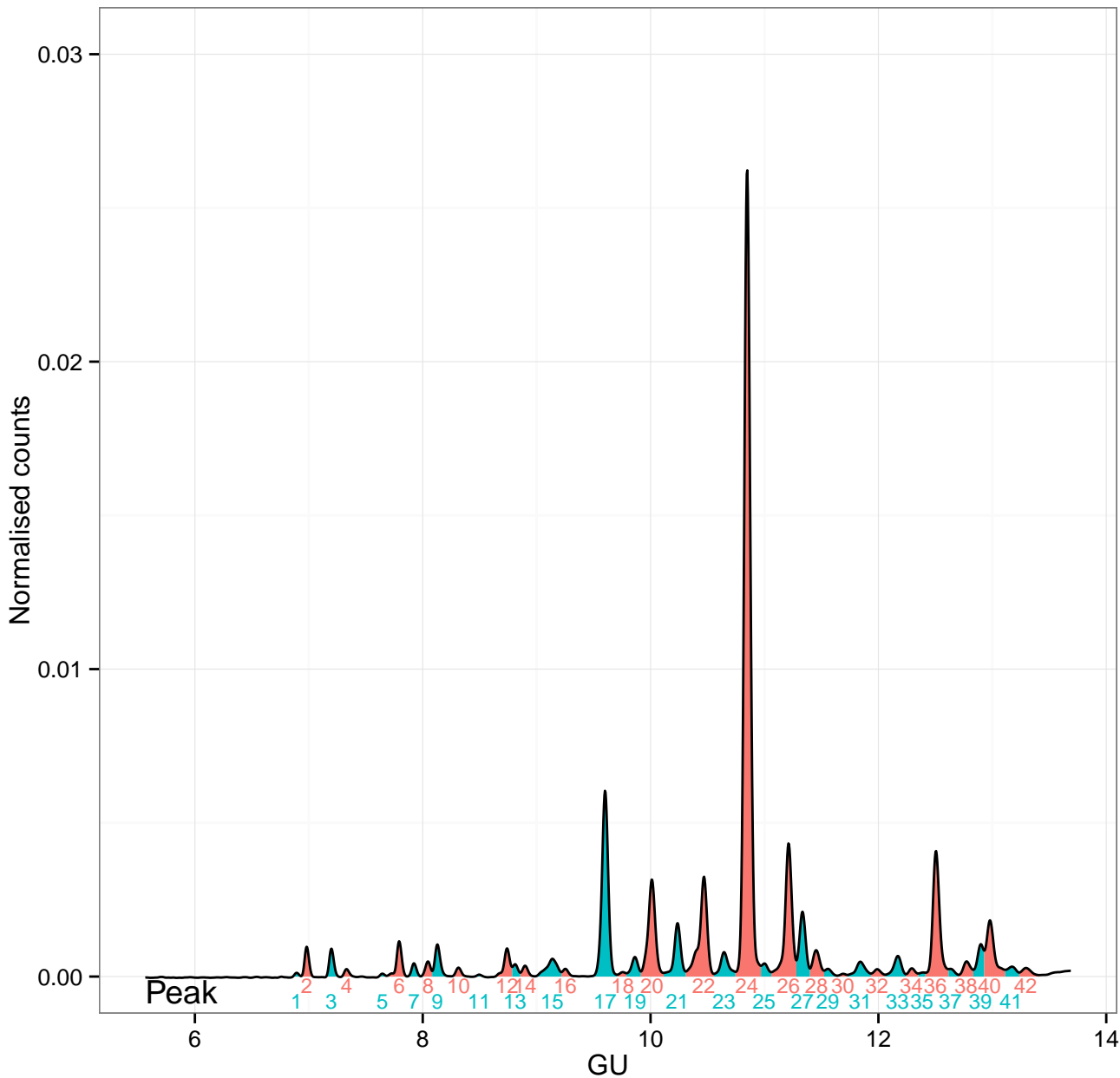

S-3

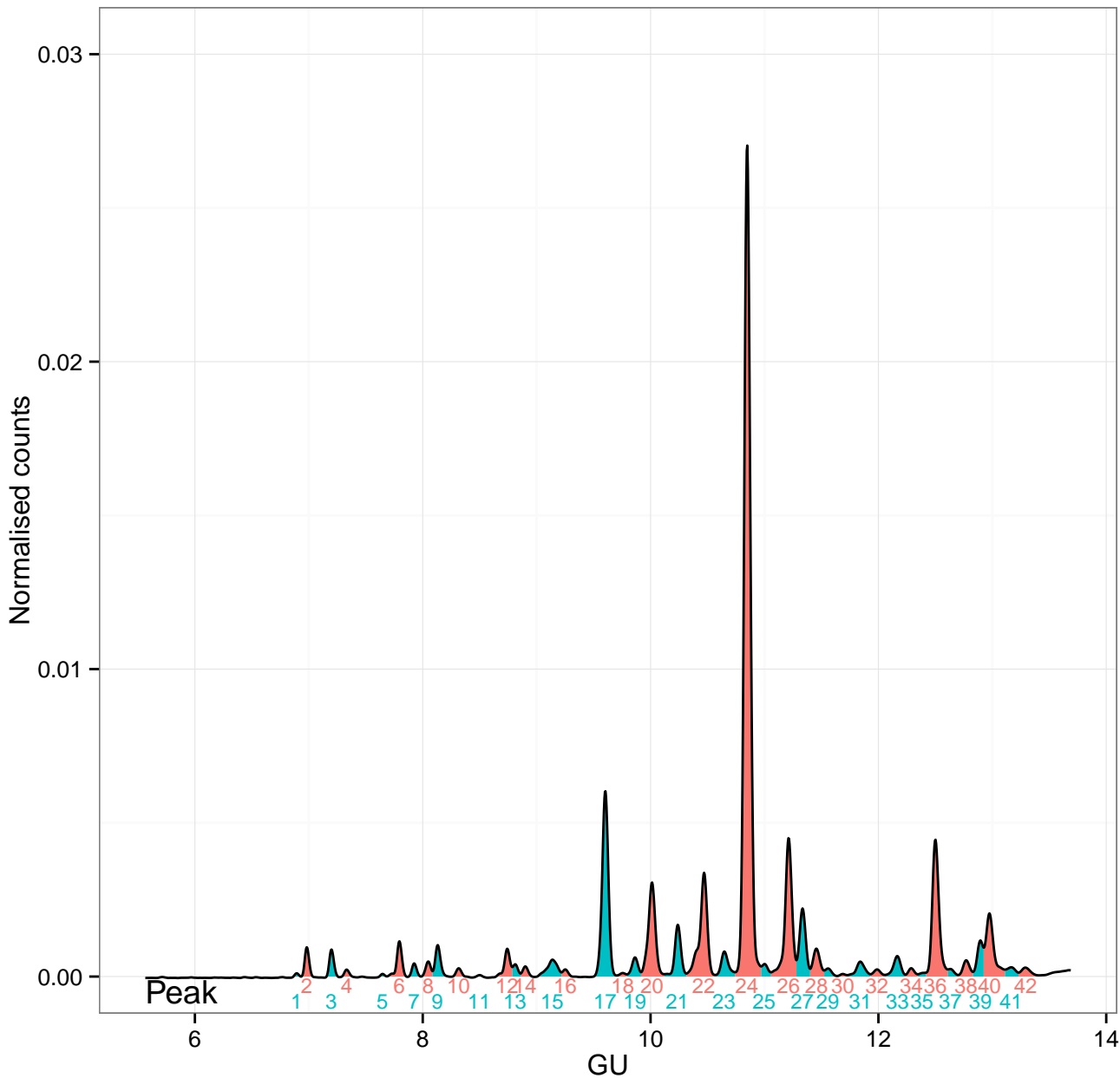

T-1

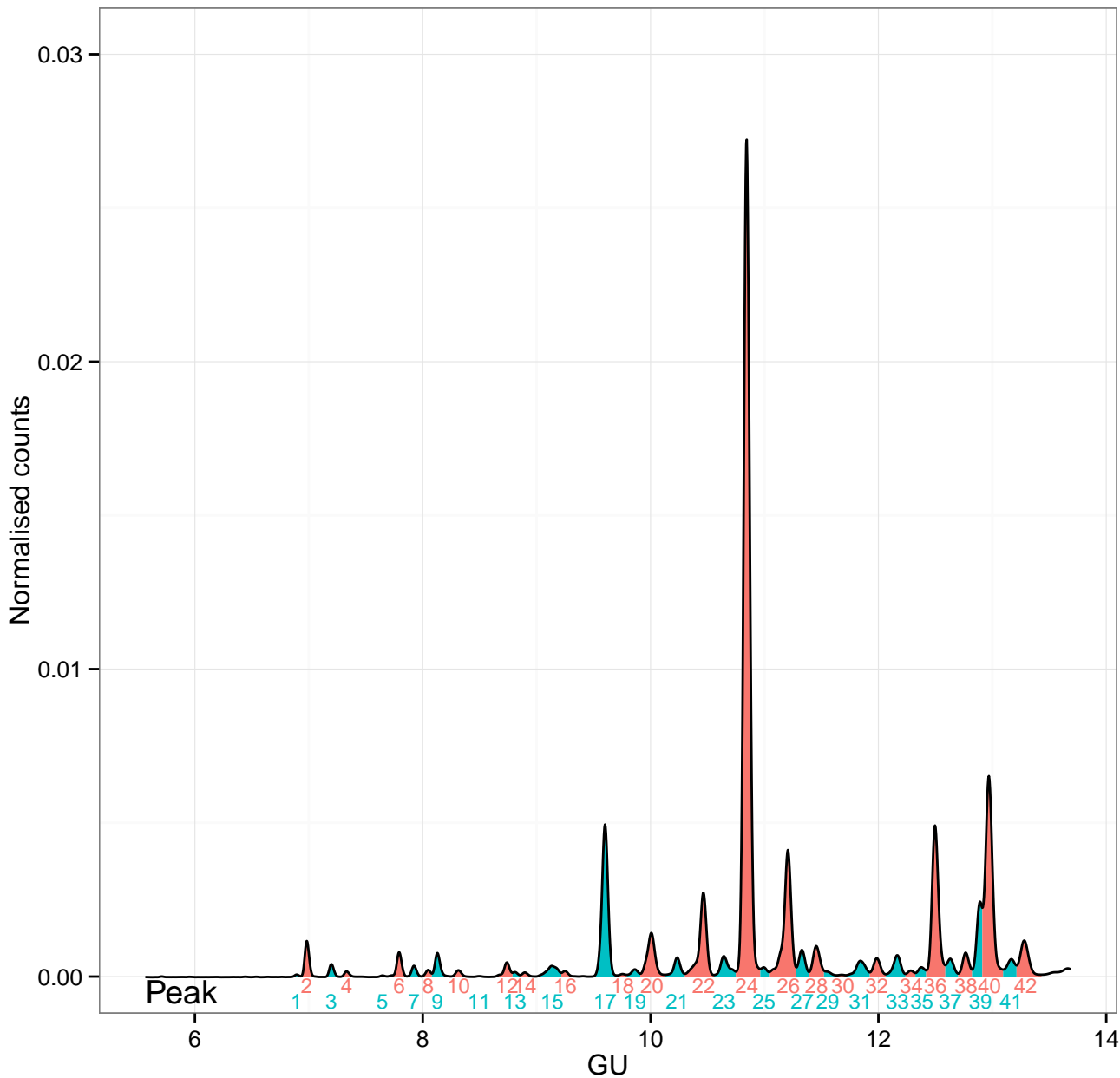

T-2

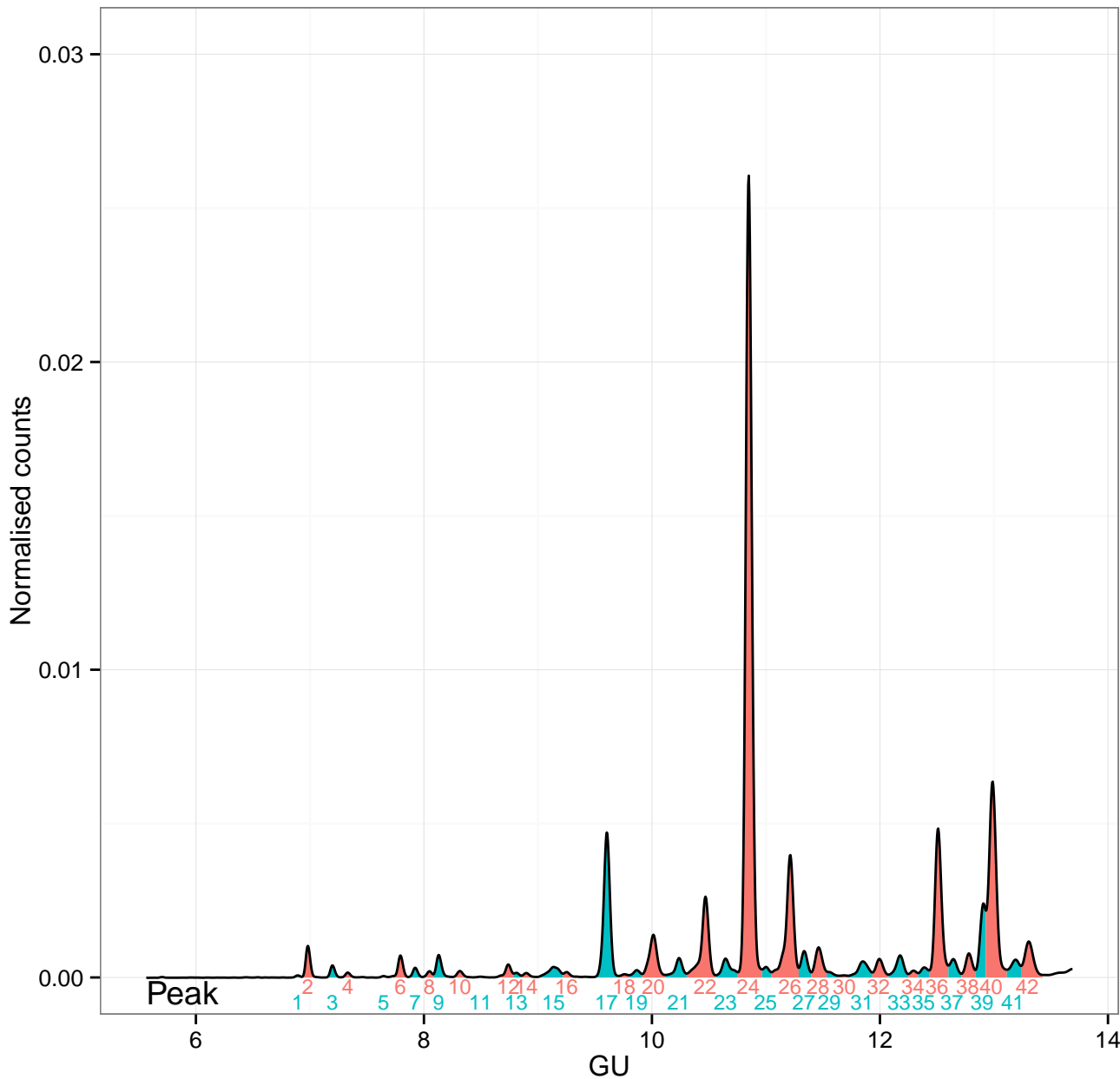

T-3

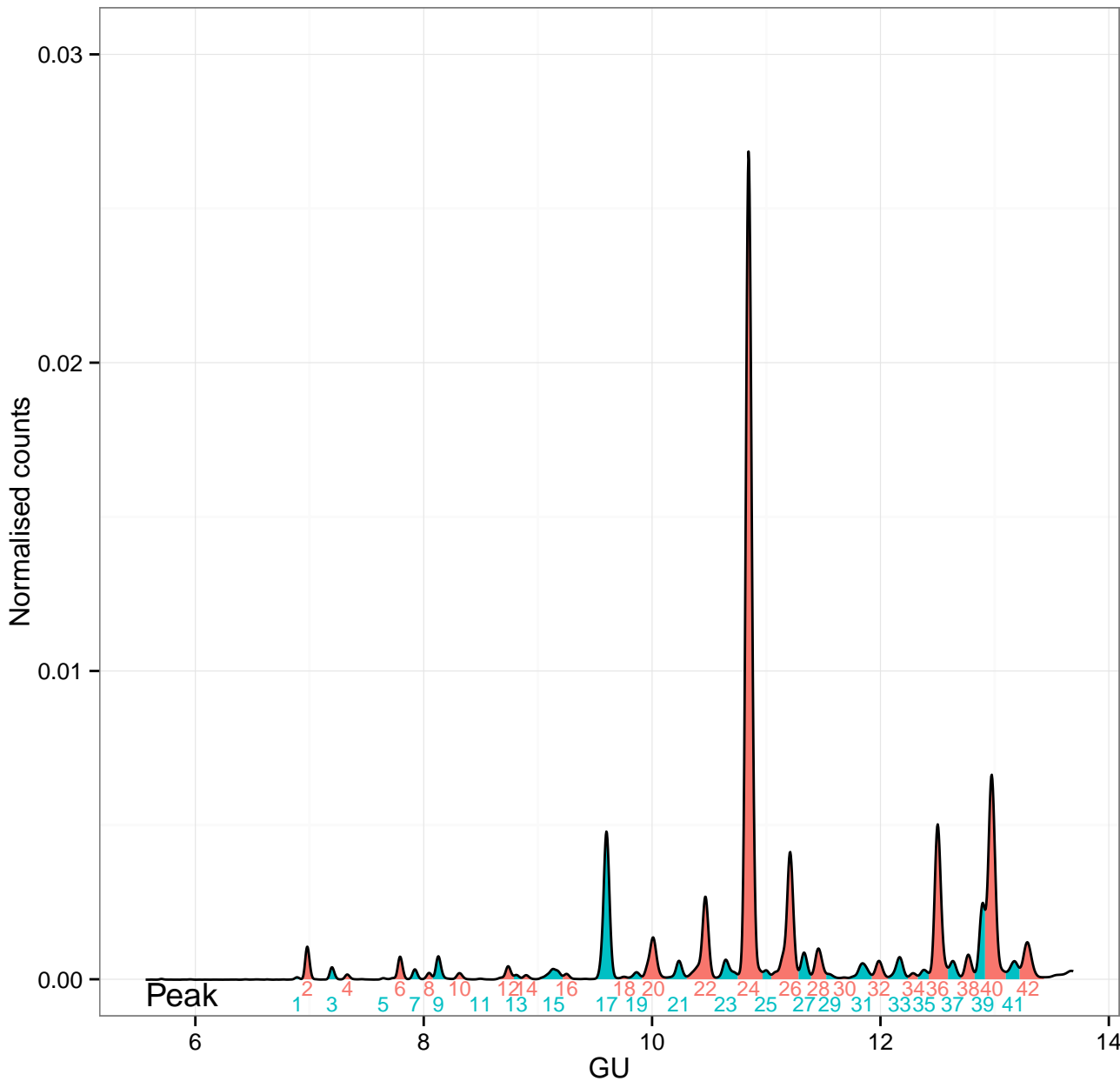

U-2

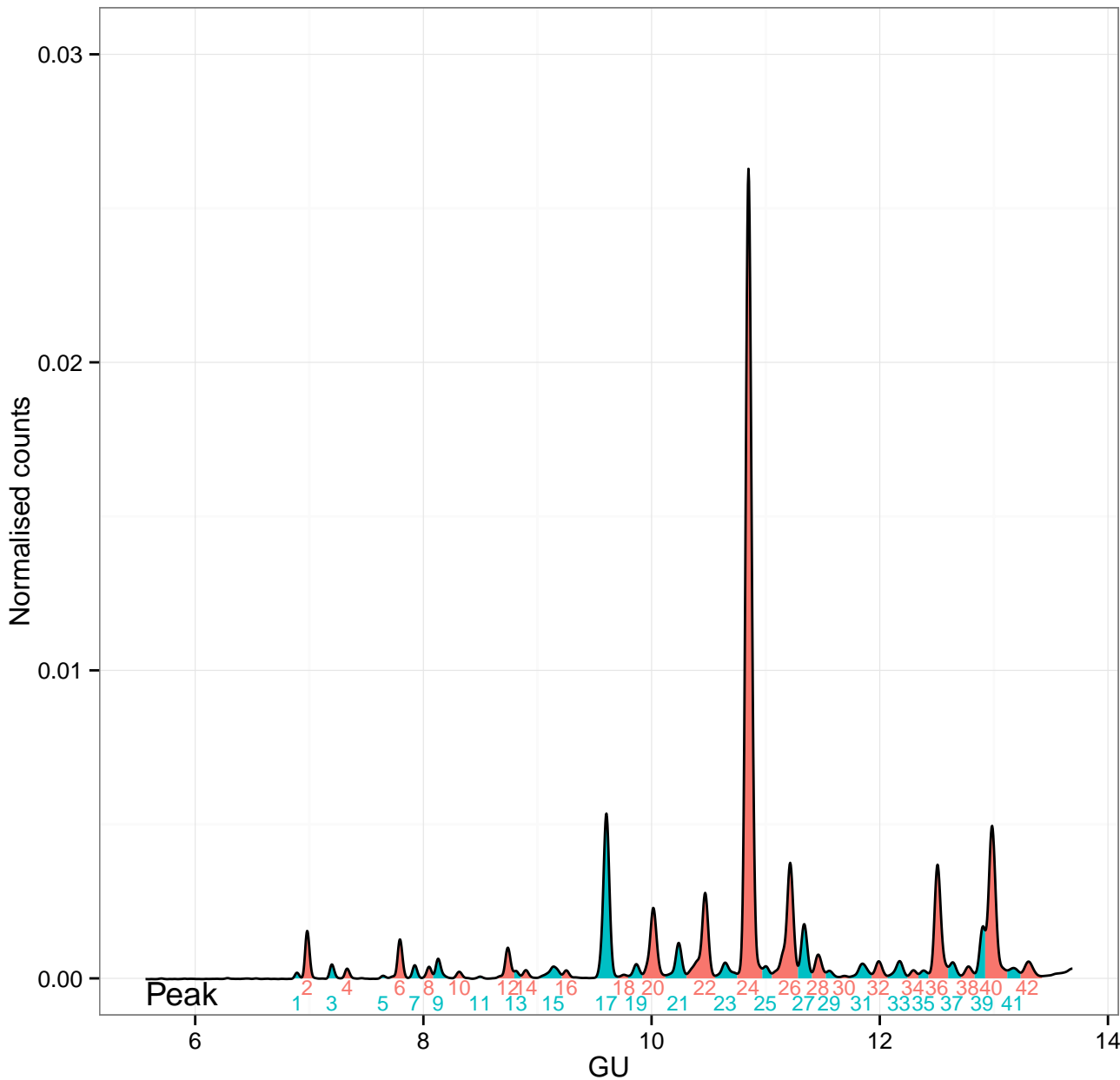

U-3

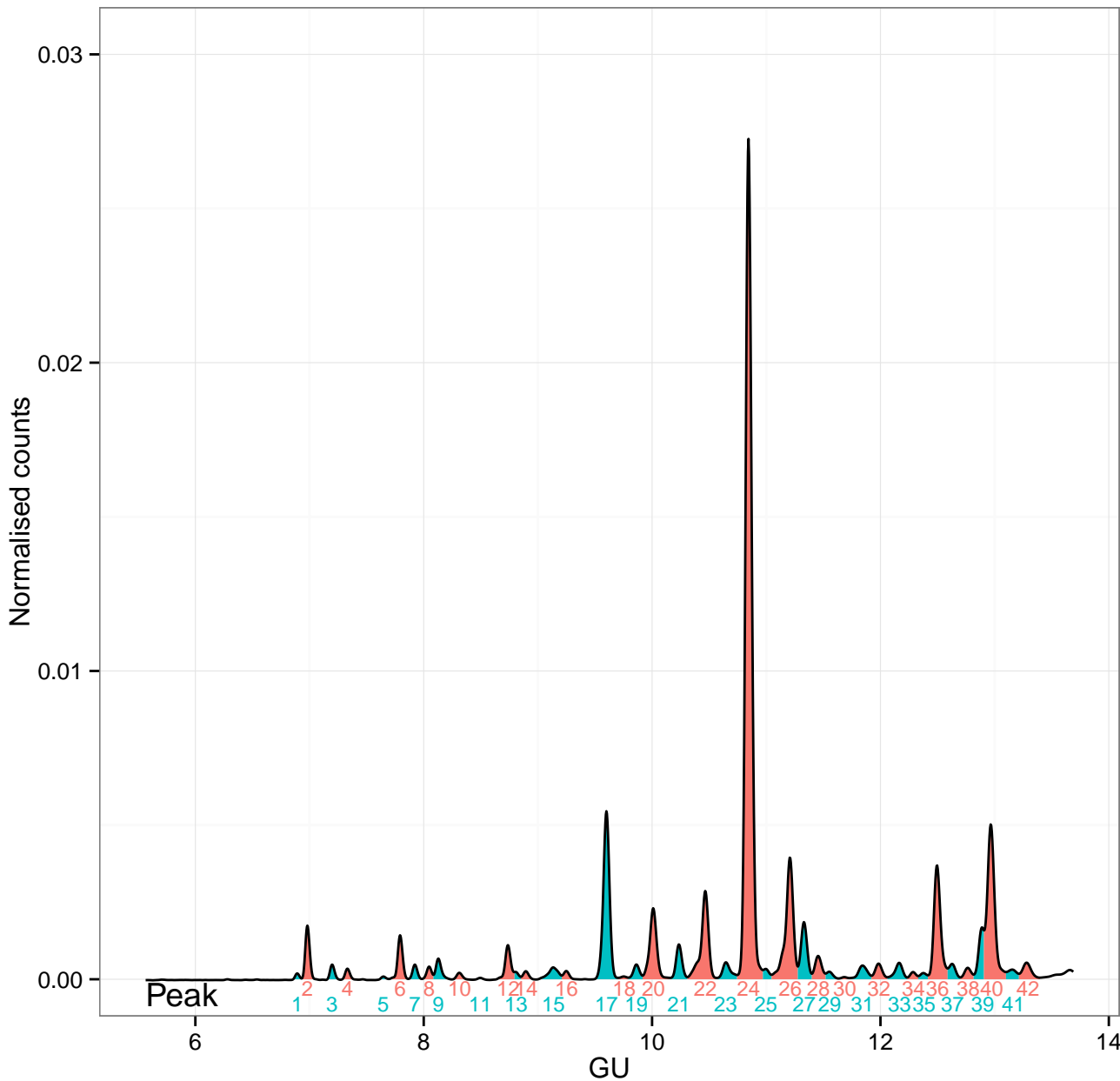

V-1

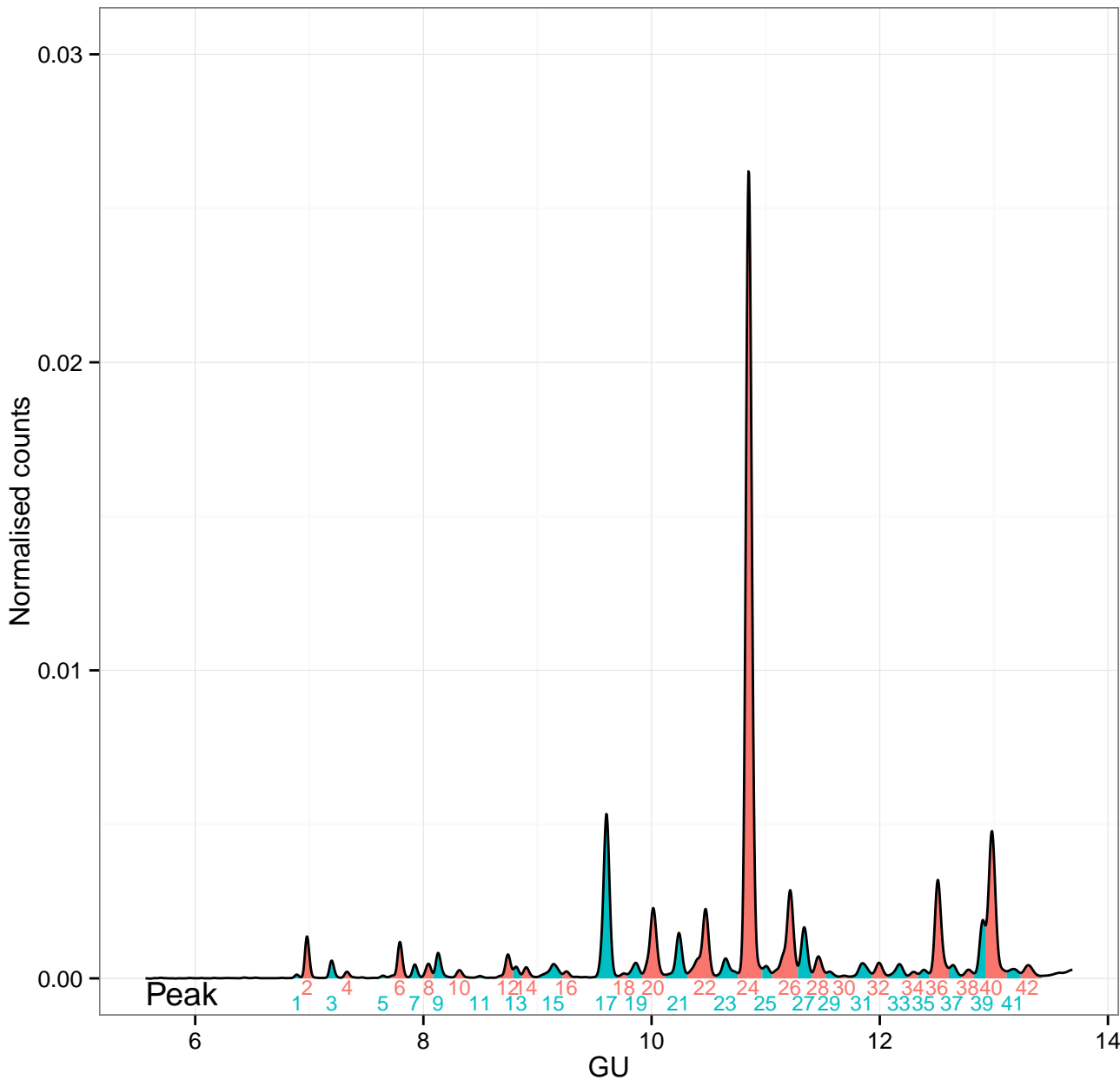

V-2

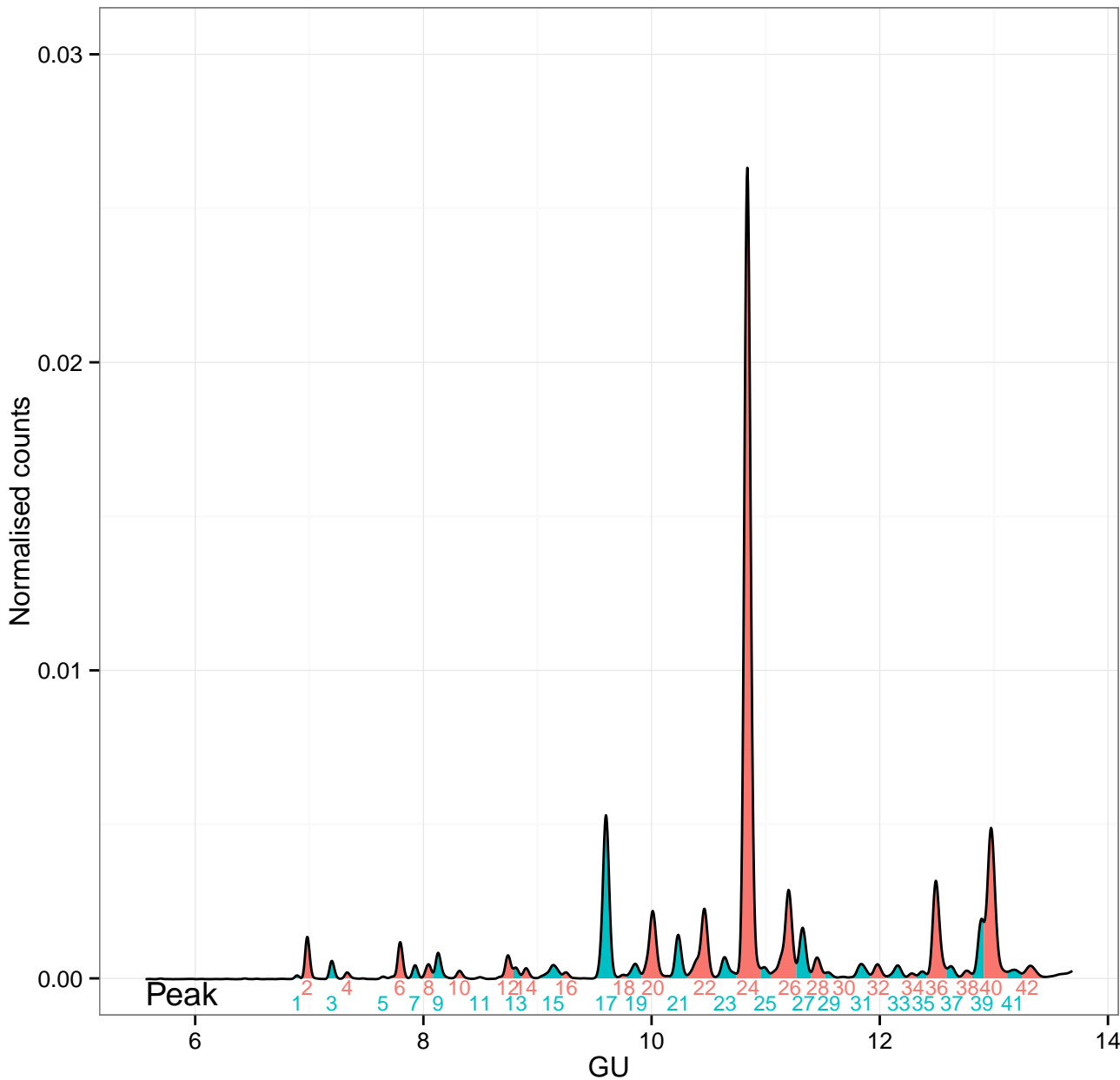

V-3

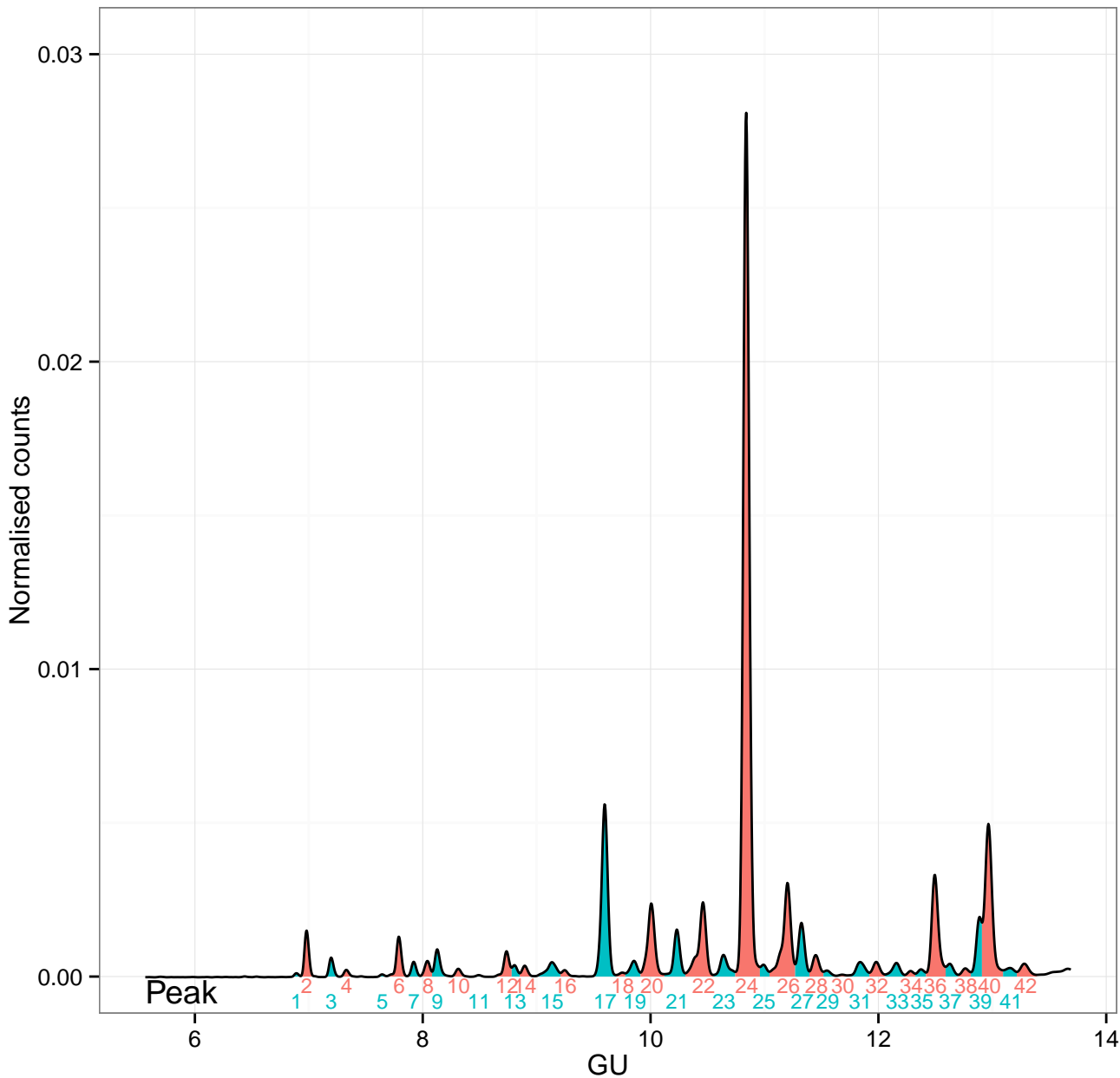

W-1

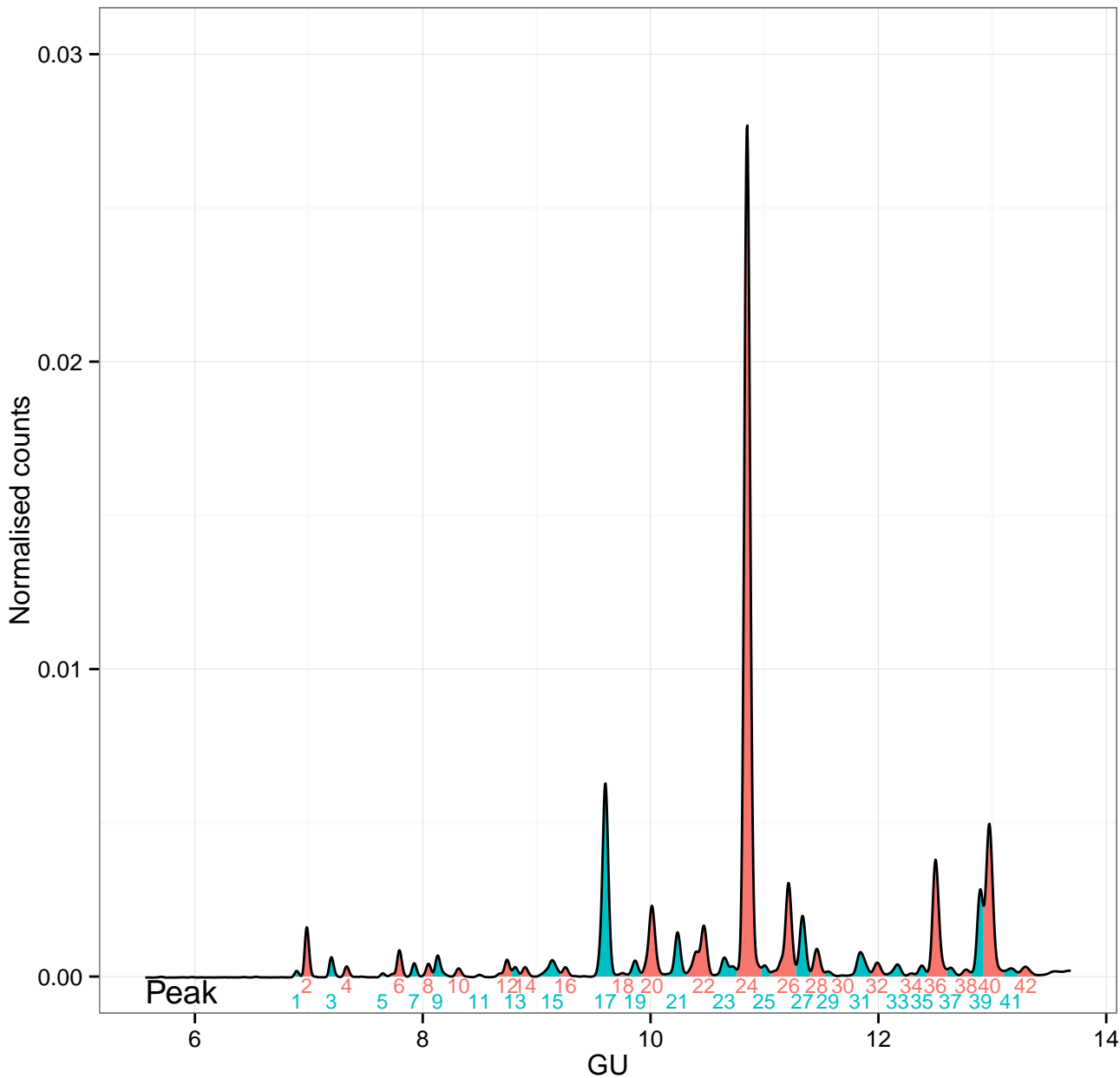

W-2

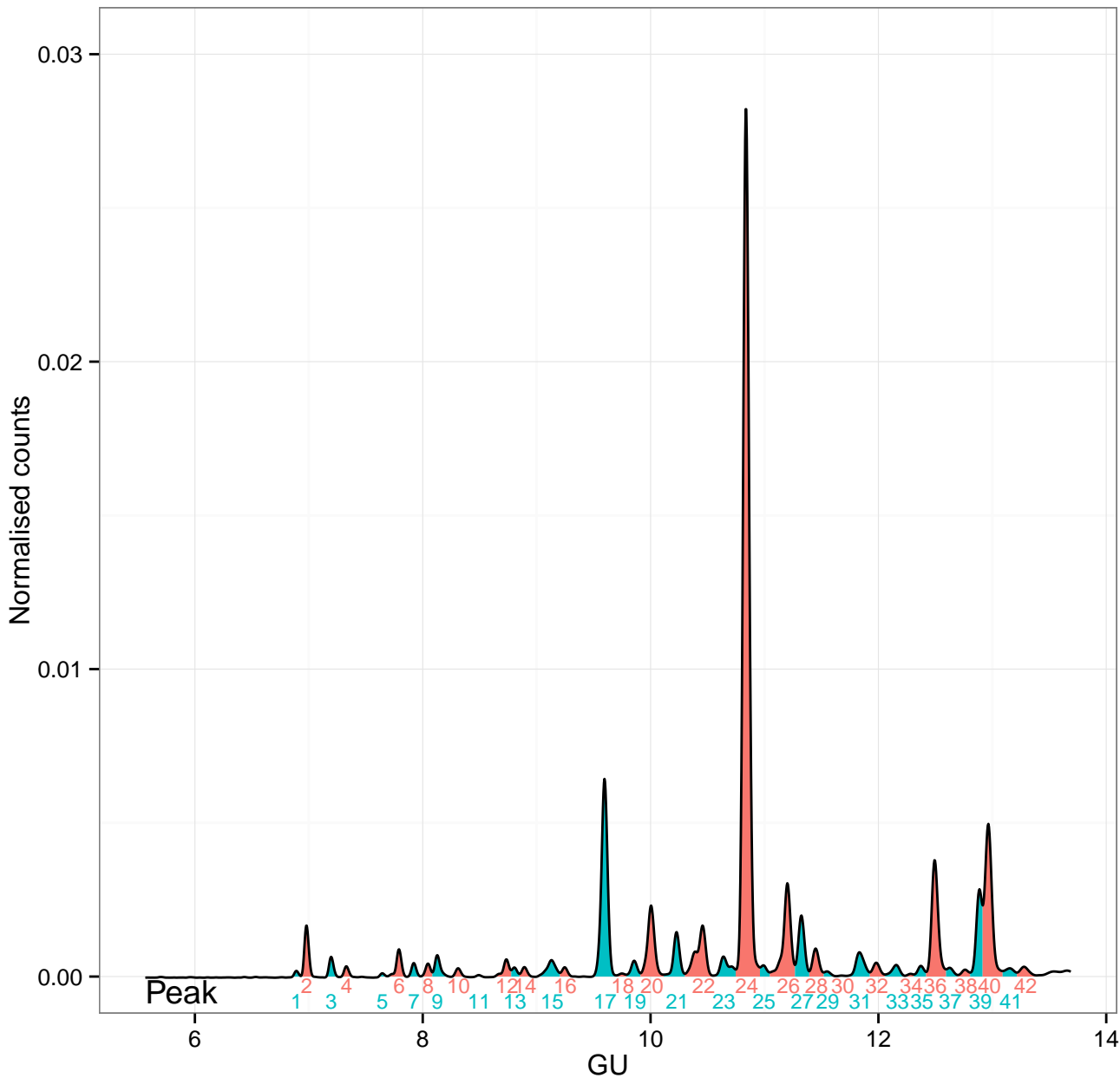

W-3

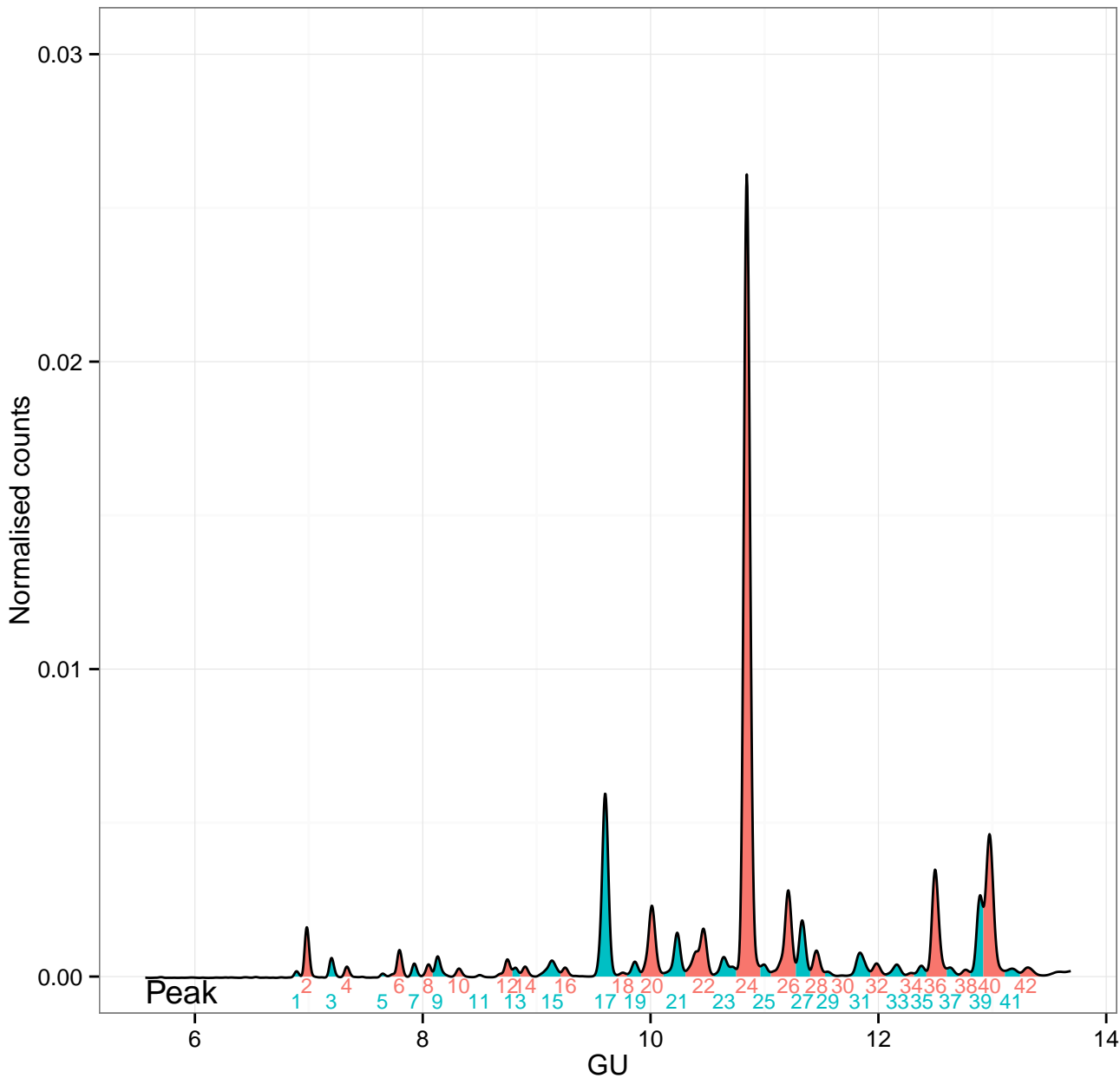

X-1

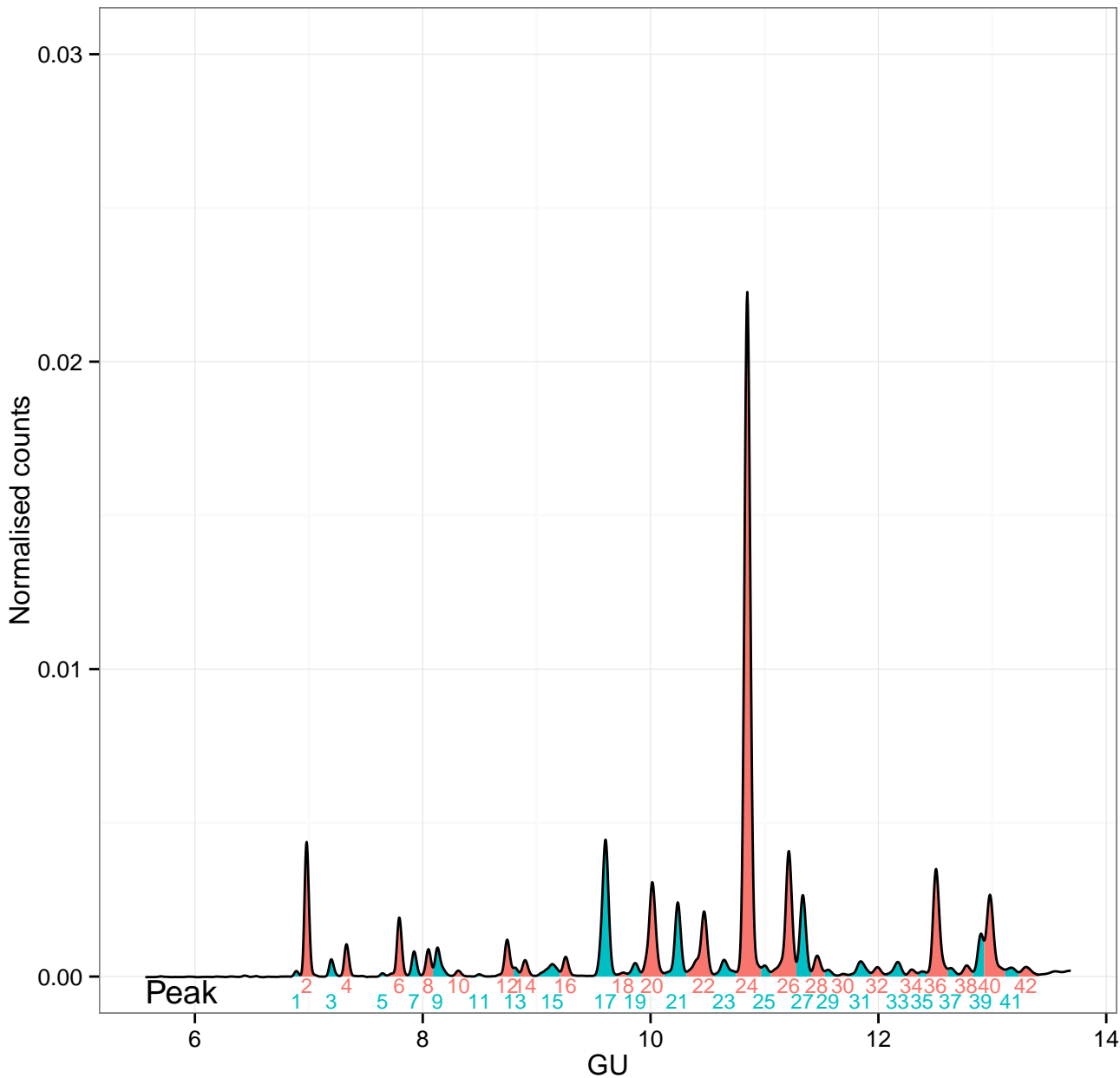

X-3

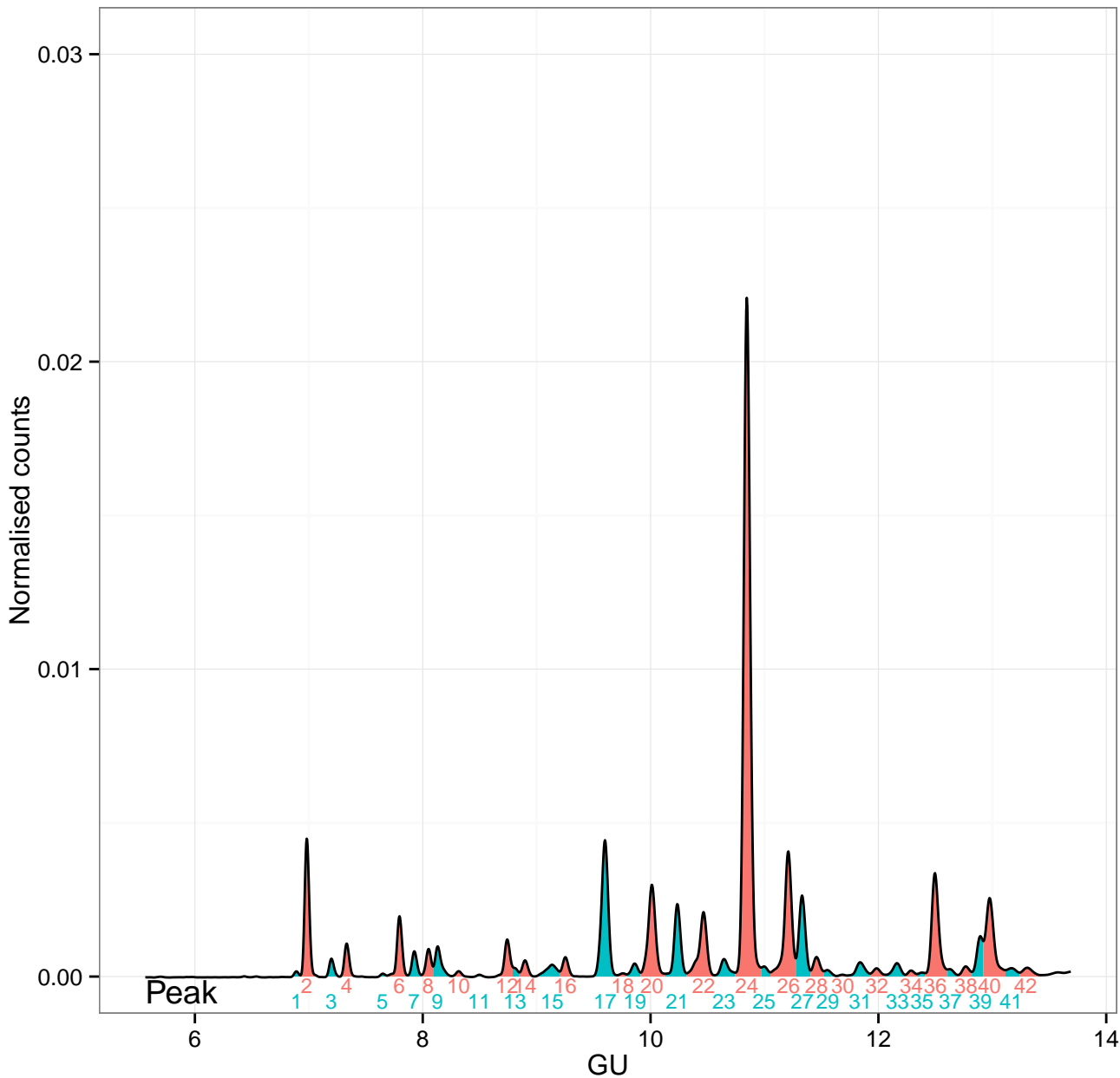

Supplement: S2 Fig — (PDF) [file pone.0123028.s004.pdf]

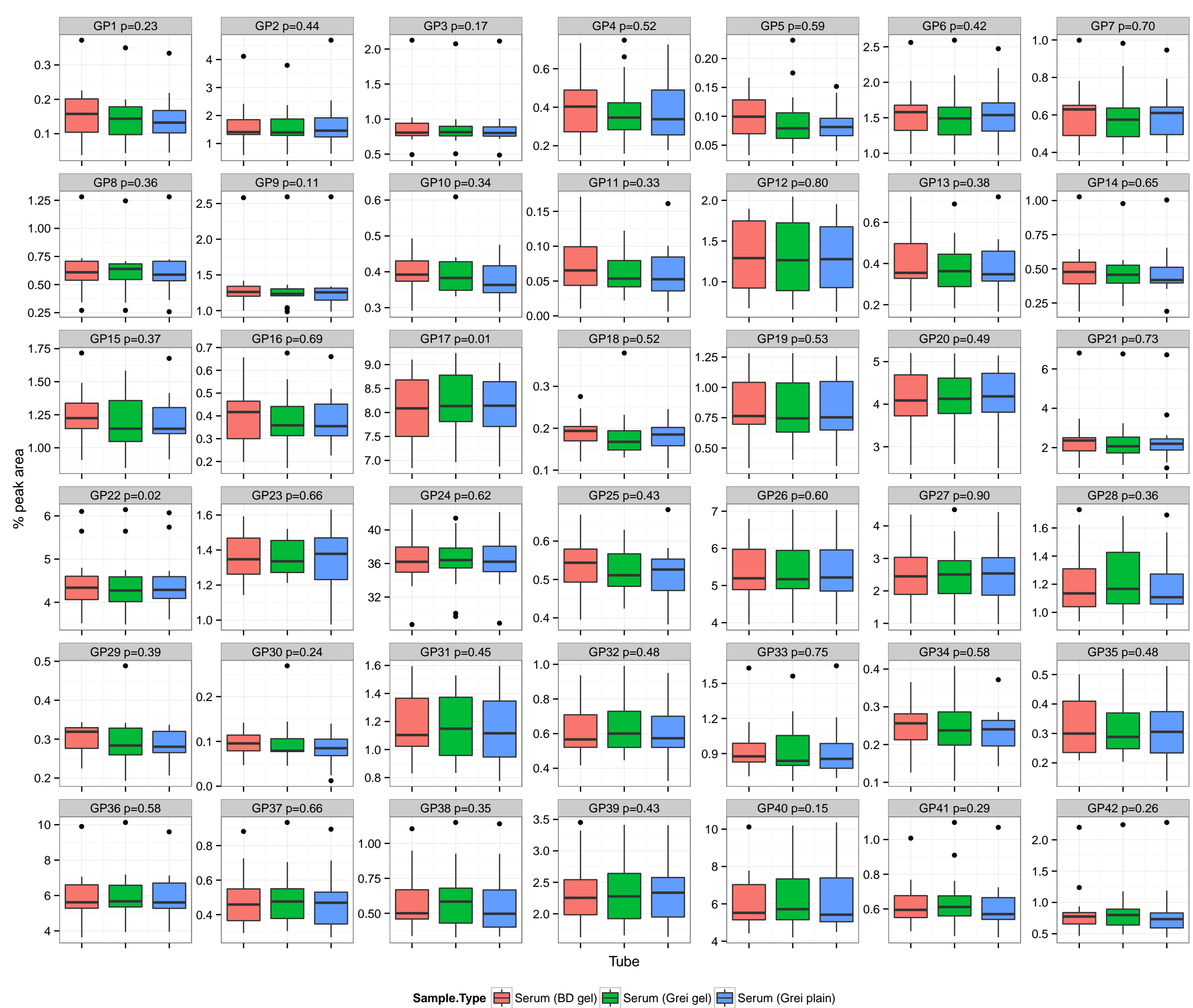

Supplement: S3 Fig — GP = Glycan peak. (PDF) [file pone.0123028.s005.pdf]
